# A conformationally adaptable tetrahedral cage with different guest encapsulation models†

**DOI:** 10.1039/d5sc01474c

**Published:** 2025-05-09

**Authors:** Hua Tang, Yuyang Lu, Yongwei Qian, Chenqi Ge, Jiyong Liu, Hongliang Chen, Hao Li

**Affiliations:** a Stoddart Institute of Molecular Science, Department of Chemistry, Zhejiang University Hangzhou 310058 China lihao2015@zju.edu.cn hongliang.chen@zju.edu.cn; b Zhejiang-Israel Joint Laboratory of Self-Assembling Functional Materials, ZJU-Hangzhou Global Scientific and Technological Innovation Center, Zhejiang University Hangzhou 311215 China

## Abstract

Through imine condensation, a tetrahedral cage-shaped molecule was obtained in a one-pot manner, which comprises four trisamino vertices and four trisformyl faces each containing three *meta*-methyl-pyridyl units. Within the cage framework, each of the twelve pyridines undergoes pivoting around the corresponding imine-pyridine-phenyl axle, endowing the cage with adaptable conformations. By modulating the number of pyridyl nitrogen atoms that point inwards, the cage can switch the effective volume of its cavity where a wide range of guest molecules including quaternary ammoniums, or different amounts of alkali cations, are encapsulated, by utilizing dipole–cation forces and/or hydrogen bonding. This behavior is reminiscent of biological enzymes that can adjust their conformations to optimize binding affinity.

## Abstract 2

A self-assembled tetrahedral cage will undergo a step conformational change when binding the cationic guests.

## Graphical abstract

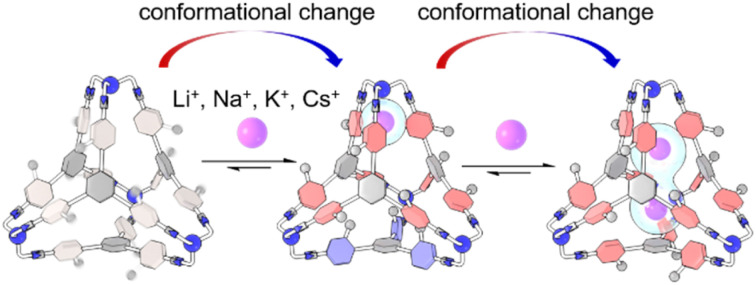

## Introduction

Biological acceptors can precisely modulate their conformations to optimize their binding affinity towards target substrates.^1^ A typical example of adapting conformations in regulation of guest upload and release is hemoglobin.^2^ This enzyme cooperatively accommodates four oxygen molecules in one conformation and switches to another that favors the release of all oxygens. This behavior enhances the ability of hemoglobin to deliver oxygen. In order to unravel the underlying mechanism of how these conformational change behaviors occur, one of the often-used approaches is to synthesize artificial hosts, whose conformations can be modulated precisely with different guest binding models.^3^ Various metal–ligand coordinative macrocycles^4,5^ and cages^6–12^ have been developed for this purpose. Their conformations can be adjusted upon recognition of different guests, and in some cases, multiple guests could be recognized within the host cavities in either a positive^13–16^ or negative^17,18^ cooperative manner. As a comparison, using purely covalent hosts^19–24^ to achieve conformationally adaptive guest binding has been much less successful. Purely covalent hosts self-assembled *via* dynamic covalent chemistry^25–30^ often comprise relatively rigid building blocks, in order to suppress entropy loss in self-assembly for the sake of high yields. The rigidity, on the other hand, would jeopardize the adaptability of conformations. Examples^31,32^ of purely covalent cages, which can incorporate the advantages of both high yielding synthesis and adaptability of conformation with different binding modes, are still rare.

Here, we obtained a tetrahedral molecule in high yield (*i.e.*, 88%), by condensing four equivalents of trisamine as vertices and four trisformyl residues as faces each bearing three *meta*-methyl-pyridyl units. The relatively high yield of the cage results from intramolecular forces including CH⋯π interactions. Within the cage framework, each of the twelve *meta*-methyl-pyridyl units undergoes free pivoting motion around the corresponding imine-pyridyl-phenyl axle, allowing the cage to adopt different conformations. In the absence of guests, on average, six of the twelve pyridyl nitrogen atoms point inwards while the others point out, implying that the “free” cage cavity is occupied by six methyl units. The cage can adapt its conformation to recognize a wide range of guests, by switching or decreasing the number of methyl units that are located inside the cavity. The pyridyl units afford the cage the ability to recognize Lewis acidic guests including quaternary ammoniums and alkali cations, by providing hydrogen bonding and/or cation–dipole interactions. For example, by adopting a conformation with nine pyridyl nitrogen atoms pointing inwards, the cage can encapsulate a single Na^+^ cation. When all twelve pyridyl nitrogen atoms are directed inward, the cage can accommodate two cations simultaneously.

## Results and discussion

**Fig. 1:**
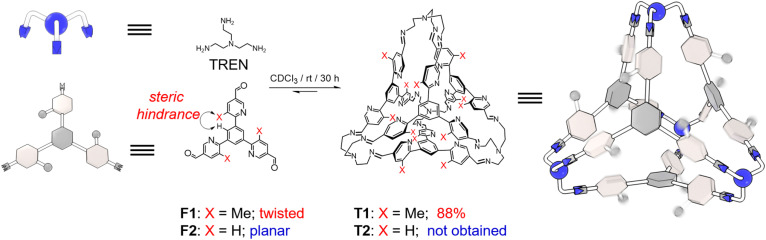
Structural formula and graphic representation of a tetrahedral cage T1 by condensing F1 and TREN in CDCl_3_. F1 adopts a twisted conformation due to the steric hindrance between the methyl unit and a central phenyl proton, which proved to be of importance in the cage self-assembly. A putative tetrahedron T2, however, was not obtained by using an analogous formyl precursor F2 and TREN, on account of the planar conformation of T2.

The triangular trisformyl compound F1 was synthesized *via* Suzuki coupling. In F1, each of the formyl units is grafted at the 5-position of a *meta*-methyl-pyridyl unit. F1 was combined with tris(2-aminoethyl)amine (TREN), a commercially available trisamine, in CDCl_3_. After placing the solution at room temperature for 30 h, the recorded ^1^H NMR spectrum revealed a new set of sharp resonances corresponding to a tetrahedral cage T1 (Fig. 1 and S14, ESI†). This tetrahedron is composed of four TREN residues as the vertices and four F1 residues as the faces. The self-assembly yield of T1 was determined to be 88%, by integrating and comparing the resonances corresponding to the formyl precursor F1 and the product T1 relative to an internal standard in the NMR sample, whose concentration remained constant before and after self-assembly (Fig. S14, ESI†). Such a high yield of T1 resulted from the dynamic nature of imine formation, during which error checking was allowed so that the system was able to search for its thermodynamic minimum. A pure solid-state sample of T1 was isolated in 57% yield *via* crystallization by diffusion of isopropyl ether into its chloroform solution. This solid was then re-dissolved in CDCl_3_ for the ^1^H NMR spectrum, in which T1 was the only observable product, indicating that no decomposition occurred during isolation. The formation of T1 was further confirmed by mass spectrometry (Fig. S13, ESI†) and ^1^H-DOSY NMR spectroscopy (Fig. S12, ESI†).

As a comparison, F2, an analogue of F1 whose Me units are replaced with protons, was also combined with TREN under the same conditions. However, the formation of the putative tetrahedral cage T2 was not observed (Fig. S16, ESI†). The self-assembly of T2 was not successful either, even when employing quaternary ammonium species as potential templating agents. It has been reported by us^33–36^ that the formation of tetrahedra was favored by the intramolecular CH⋯π interactions between the two adjacent phenylene units in each corner of the cage. In the case of T1, the occurrence of CH⋯π interactions requires pyridyl in each vertex to orientate in an edge-in manner. This twisted conformation indeed occurs in the precursor F1 before self-assembly, because of the steric hindrance between the methyl units in the pyridine and the corresponding protons in the central phenyl ring. As a comparison, F2 adopts a relatively planar conformation due to the absence of methyl units, making the formation of the putative cage T2 more energy demanding.

**Fig. 2:**
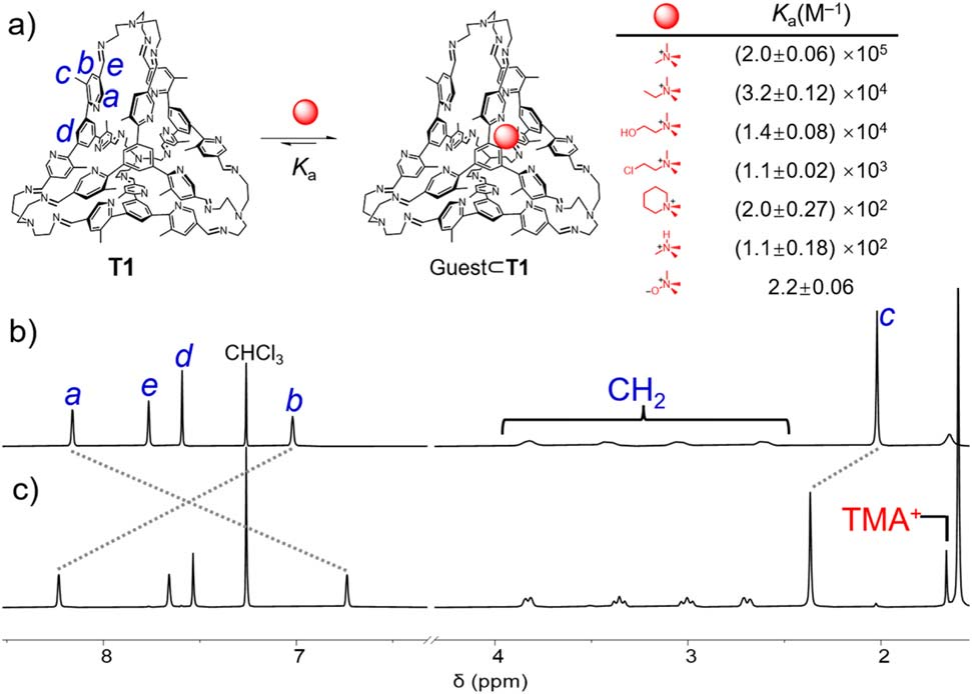
(a) Recognition of a guest within the cavity of the tetrahedron T1. The structures of the guests and the corresponding binding constants are listed. The partial ^1^H NMR spectrum of T1 (400 MHz, CDCl_3_, 298 K) (b) before and (c) after 1 equiv. of TMA^+^·Cl^−^ was added.

The presence of CH⋯π interactions within the framework of T1 was confirmed by its ^1^H NMR spectrum (Fig. 2b). The resonances corresponding to the two protons in the *ortho* positions relative to the imine bond, denoted as protons *a* and *b*, underwent remarkable upfield shifts by 0.81 and 1.06 ppm, respectively, compared to the precursor F1 (Fig. S3, ESI†). These shifts suggest that in the framework of T1, both *a* and *b* spend a substantial amount of time near the surface of an adjacent pyridyl unit, resulting in a shielded magnetic environment. Considering that only one set of sharp resonances was observed, it is thus reasonable to propose that each pyridyl underwent fast pivoting motion along the phenyl-pyridyl-imine axis on the timescale of ^1^H NMR spectroscopy. Compared to F1, the resonance corresponding to the methyl unit in the cage T1 shifted upfield by 0.52 ppm, further confirming our hypothesis that the methyl units spent substantial amounts of time within the cage cavity.

The nitrogen atom in a pyridyl unit represents a typical Lewis basic binding site, endowing the cage T1 with the ability to recognize Lewis acidic guests. Upon the addition of tetramethylammonium (TMA^+^) chloride into a solution of T1 in CDCl_3_, the resonances of the latter underwent remarkable shifts (Fig. 2c). A resonance corresponding to TMA^+^ encapsulated within the cage cavity was observed at 1.66 ppm, which shifted upfield by 1.88 ppm compared to the “free” TMA^+^. This observation confirmed the recognition of TMA^+^ within the cage cavity. Compared to the “free” cage T1, the resonance corresponding to the protons *a* in the *ortho* position of nitrogen shifted upfield by 1.42 ppm, while those corresponding to the methyl units and the protons *b* near the methyl units shifted downfield by 0.35 and 1.21 ppm, respectively. These observations clearly indicated that upon recognition of TMA^+^, all the methyl units in the cage framework moved out from the cage cavity, and simultaneously all the nitrogen atoms moved inside to form cation–dipole interactions with the guest and N⋯HC hydrogen bonds with the methyl protons in TMA^+^. The complex TMA^+^⊂T1·Cl^−^ underwent slow host–guest association/dissociation exchange on the timescale of ^1^H NMR spectroscopy, as inferred from the fact that the “free” cage and the host–guest complex exhibited two independent sets of resonances. By integrating the resonances corresponding to the “free” cage, “free” guest, and the host–guest complex, the binding constant (*K*_a_) of TMA^+^⊂T1·Cl^−^ was determined to be (2.0 ± 0.06) × 10^5^ M^−1^ (Fig. S25, ESI†). Using UV-vis absorption spectroscopy, *K*_a_ was measured to be (5.9 ± 0.4) × 10^4^ M^−1^ (Fig. S24, ESI†), which is in close agreement with the ^1^H NMR spectroscopic results. We also investigated the possibility of the cage to recognize other quaternary ammonium guests. After adding tetraethylammonium (TEA^+^) chloride, no changes were observed in the ^1^H NMR spectrum of T1, indicating that the size of TEA^+^ is too large to fit within the cavity of T1. In the case of trimethylethylammonium (TMEA^+^) with I^−^ counterions, as well as choline, chlormequat, mepiquat and trimethylamine hydrochloride (TMAH^+^·Cl^−^), whose counterions are all Cl^−^, the binding constants were measured to be (3.2 ± 0.12) × 10^4^ M^−1^ (Fig. S69, ESI†), (1.4 ± 0.08) × 10^4^ M^−1^ (Fig. S75, ESI†), (1.1 ± 0.02) × 10^3^ M^−1^ (Fig. S76, ESI†), (2.0 ± 0.27) × 10^2^ M^−1^ (Fig. S89, ESI†) and (1.1 ± 0.18) × 10^2^ M^−1^ (Fig. S94, ESI†), respectively. These binding constants are one or two orders of magnitude lower than that of TMA^+^. One explanation is that these guests have less complementary sizes relative to the cage cavity. We also attempted to use T1 to recognize neutral compounds with complementary sizes. However, only one zwitterion namely trimethylamine *N*-oxide was observed to be encapsulated within the cage cavity of T1. The binding constant was measured to be only 2.2 ± 0.06 M^−1^ by using the ^1^H NMR spectrum (Fig. S80, ESI†), which is lower by nearly four or five orders of magnitude compared to the cationic guest TMA^+^. This result indicated that the binding between T1 and the aforementioned quaternary ammonium guests mainly relied on dipole–cation interactions.

**Fig. 3:**
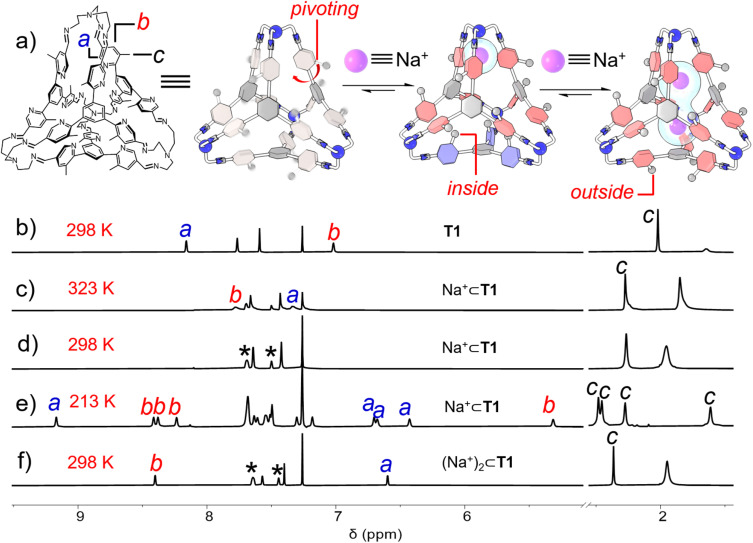
(a) Graphical representation of the allosteric regulated binding behaviors of cage T1 toward one of the two Na^+^. Partial ^1^H NMR spectra (400 MHz, CDCl_3_) of (b) T1 recorded at 298 K, Na^+^⊂T1·BArF^−^ recorded at (c) 323 K, (d) 298 K, and (e) 213 K, and (f) (Na^+^)_2_⊂T1·2BArF^−^ recorded at 298 K. The resonances corresponding to the protons *a*, *b*, and *c* in the cage framework were labelled with the corresponding letters in the spectra. The resonances corresponding to the BArF^−^ counterions were labelled with black stars.

The ability of the cage to recognize alkali cations was then investigated. Sodium tetrakis[3,5-bis(trifluoromethyl)phenyl]borate (Na^+^·BArF^−^) was added into a solution of T1 in CDCl_3_. The BArF^−^ counterion served to enhance the solubility of Na^+^ in CDCl_3_. Upon the addition of one equiv. of Na^+^, a remarkable change occurred to the ^1^H NMR spectrum of T1 (Fig. 3d). The resonances corresponding to the protons *e* in imine units and protons *d* in the central phenyl units only underwent modest shifts. As a comparison, the resonances corresponding to the protons *a* and *b* in the pyridyl units were observed to broaden out into the baseline and become almost unobservable. This observation indicated that after recognition of one Na^+^ cation within the cage cavity, the pivoting of pyridyl units was significantly slowed down compared to the “free” cavity, on account of cation–dipole interactions between Na^+^ and pyridyl nitrogen atoms. In fact, the resonances corresponding to the protons *a* and *b* gradually sharpened at elevated temperatures. These two resonances were observed at 7.33 and 7.78 ppm, respectively at 323 K (Fig. 3c), implying an upfield and a downfield shift by 0.83 and 0.75 ppm respectively relative to the “free” cage. This observation is not surprising, because after accommodation of a Na^+^ guest, more nitrogen atoms in the pyridyl units would point inwards, in order to enhance the cation–dipole interactions with the cationic guest. The ^1^H NMR spectrum of Na^+^⊂T1·BArF^−^ was also recorded at 213 K (Fig. 3e). At such a low temperature, each resonance of the cage observed at room temperature splits into four distinct peaks. For example, the resonances corresponding to the methyl units (protons *c*) were observed at 2.48, 2.45, 2.27, and 1.61 ppm respectively, while those corresponding to the protons *b* in the *ortho* positions of the methyl units were observed at 8.41, 8.38, 8.24, and 5.31 ppm, respectively. In both cases, one of the four resonances undergoes a remarkable upfield shift while the other three are located relatively downfield. The resonances corresponding to the protons *a* beside pyridyl nitrogen atoms have the opposite trend, *i.e.*, one was observed at a downfield position (*i.e.* 9.17 ppm), while the other three were located upfield (*i.e.*, 6.70, 6.68, and 6.43 ppm, respectively). Considering that the cage bears twelve pyridyl units, the appearance of four resonances indicated that the framework of the complex Na^+^⊂T1·BArF^−^ has a *C*_3_ axis of symmetry. The Na^+^ guest coordinates with the three pyridyl units in one corner of the tetrahedron, driving all three methyl in this corner to reside outside the cage cavity. In each of the other three corners where the cation is absent, one methyl points inwards and occupies the cavity while the other two reside outside, driven by intramolecular Me⋯N hydrogen bonding. At 213 K, the motion of Na^+^ between the four corners of the cage occurred at a relatively slow rate on the timescale of ^1^H NMR spectroscopy. However, raising temperature will speed up this motion, making the four corners of the cage chemically equivalent. This proposition is consistent with the observation that at room temperature or 323 K, only one set of resonances of Na^+^⊂T1 was observed.

Addition of another equiv. of Na^+^ into Na^+^⊂T1·BArF^−^ led to the appearance of a new set of resonances in the corresponding ^1^H NMR spectrum (Fig. 3f), corresponding to a ternary complex namely (Na^+^)_2_⊂T1·2BArF^−^. Compared to Na^+^⊂T1·BArF^−^, the resonances corresponding to the methyl units and protons *b* undergo downfield shifts by 0.09 and 0.62 ppm respectively, while that of the proton *a* shifted upfield by 0.73 ppm. These shifts indicate that after the addition of the second equiv. of Na^+^, all twelve pyridyl units point inward due to the occurrence of cation–dipole interactions and thus drive all twelve methyl units to reside outside the cage cavity. Unlike Na^+^⊂T1·BArF^−^ whose ^1^H NMR spectrum is temperature dependent, the ^1^H NMR spectrum of (Na^+^)_2_⊂T1·2BArF^−^ barely changed at all temperatures above 213 K (Fig. S39, ESI†), indicating that the two cationic guests underwent fast movement between the four corners, marking all twelve pyridyl units chemically equivalent. The addition of excess Na^+^ into the solution of (Na^+^)_2_⊂T1·2BArF^−^ resulted in no further changes in the ^1^H NMR spectrum, indicating that the accommodation of the third or more cations did not occur, due to coulombic repulsion.

The ability of T1 to accommodate other alkali cations such as Li^+^, K^+^ and Cs^+^ (Fig. S40–S59, ESI†), whose counterions are BF_4_^−^, BArF^−^ and NTf_2_^−^ respectively, was also investigated. The ^1^H NMR spectroscopy results indicated that these cations exhibited similar host–guest recognition behaviors with the cage to Na^+^. All the complexes underwent slow host–guest exchange on the timescale of ^1^H NMR spectroscopy. As a consequence, the corresponding binding constants (*K*_1_ and *K*_2_) were measured by integrating the relevant resonances (Table 1). In CDCl_3_, *K*_1_ values for Na^+^, K^+^ and Cs^+^ were measured to be (1.09 ± 0.02) × 10^4^ M^−1^, (2.96 ± 0.76) × 10^3^ M^−1^ and (2.94 ± 0.29) × 10^3^ M^−1^, respectively, while *K*_2_ values were measured to be (1.73 ± 0.47) × 10^3^ M^−1^, (7.3 ± 0.3) × 10^2^ M^−1^ and (4.4 ± 0.1) × 10^2^ M^−1^, respectively. For all the cations, *K*_1_ is larger than *K*_2_ by nearly one order of magnitude, indicating that the binding of two cations within the cage cavity exhibits negative cooperativity. This observation is attributed to coulombic repulsion, which makes the accommodation of the second cation more energetically demanding. Li^+^·BF_4_^−^ is poorly soluble in CDCl_3_. We thus measured its *K*_1_ and *K*_2_ in a 1 : 1 mixture in CDCl_3_ and CD_3_CN, which are (1.93 ± 0.2) × 10^5^ M^−1^ and (2.3 ± 0.1) × 10^2^ M^−1^, respectively. We also performed binding experiments for Na^+^ and K^+^ in this mixture solvent (Fig. S60, ESI†). In the case of K^+^, the cage exhibited little or no binding. In the case of Na^+^, T1 can only accommodate one equiv. of Na^+^, even after the addition of an excess amount of Na^+^ cation. *K*_1_ of Na^+^⊂T1 was measured to be (2.74 ± 0.17) × 10^3^ M^−1^ (Fig. S61, ESI†) in 1 : 1 CDCl_3_ and CD_3_CN, which is significantly lower than that in pure CDCl_3_ by one order of magnitude. This observation indicated that the presence of CD_3_CN suppressed the ability of the cage to accommodate cationic guests, probably because CD_3_CN acts as a competitive ligand to coordinate with the cationic guests. In addition, smaller alkali cations such as Li^+^ and Na^+^ have larger binding affinity with the cage T1 compared to their larger counterparts such as K^+^ and Cs^+^. This is not surprising, given that smaller cations have larger electron density and represent better Lewis acids. The ability of the cage to recognize transition metal cations such as Cu^+^, Ag^+^, Pd^2+^, Zn^2+^ and Cd^2+^ and T1 was also investigated (Fig. S95, ESI†). Unfortunately, the addition of these metal ions resulted in quick precipitation of cage, probably because of the formation of polymeric products on account of the coordination of the metal and pyridyl units in the cage framework.

**Table 1:** The binding constants of the cage T1 to recognize different cations

Cation	Anion	Solvent	*K*_1_ (M^−1^)	*K*_2_ (M^−1^)
Li^+^	BF_4_^−^	CDCl_3_/CD_3_CN (1 : 1 v/v)	(1.9 ± 0.2) × 10^5^	(2.3 ± 0.1) × 10^2^
Na^+^	BArF^−^	CDCl_3_	(1.1 ± 0.02) × 10^4^	(1.7 ± 0.5) × 10^3^
Na^+^	BF_4_^−^	CDCl_3_/CD_3_CN (1 : 1 v/v)	(2.7 ± 0.2) × 10^3^	Not bound
K^+^	BArF^−^	CDCl_3_	(3.0 ± 0.8) × 10^3^	(7.3 ± 0.3) × 10^2^
K^+^	BF_4_^−^	CDCl_3_/CD_3_CN (1 : 1 v/v)	Not bound	Not bound
Cs^+^	NTf_2_^−^	CDCl_3_	(2.9 ± 0.3) × 10^3^	(4.4 ± 0.1) × 10^2^

**Fig. 4:**
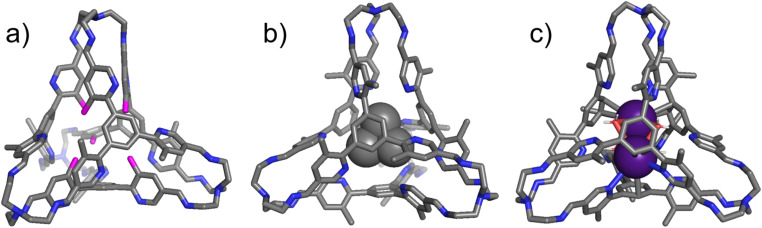
Side views of the core structures of (a) T1, (b) TMA^+^⊂T1·Cl^−^, and (c) (Cs^+^)_2_⊂T1·2NTf_2_^−^ from single crystal X-ray diffraction analysis. H white, C grey, N blue, O red, and Cs purple. Disordered solvent molecules, counterions namely Cl^−^ and NTf_2_^−^ and hydrogen atoms which are not involved in hydrogen bonding interactions are omitted for the sake of clarity. In figure (a), there are six methyl units pointing inside the cage cavity, which were labelled with pink color.

Single crystals of the “free” cage T1 (Fig. 4a), the complex TMA^+^⊂T1·Cl^−^ (Fig. 4b), and the ternary complex (Cs^+^)_2_⊂T1·2NTf_2_^−^ (Fig. 4c) were obtained by slow vapor diffusion of isopropyl ether into the corresponding solutions in chloroform. In the solid state structure of the “free” T1, six of its twelve methyl units reside inside the cage cavity and are labeled with pink color in Fig. 4a. Among these three methyl units, three are located in one corner and one resides in each of the other three corners. Close contacts were observed between the protons in these methyl units and either the adjacent pyridyl nitrogen atoms or the π-electron moieties, indicating the occurrence of either hydrogen bonding or CH⋯π interactions. In both TMA^+^⊂T1·Cl^−^ and (Cs^+^)_2_⊂T1·2NTf_2_^−^, all twelve methyl units in the cage framework were observed to locate outside the cage cavity. As a consequence, all the pyridyl nitrogen atoms point inwards, forming either hydrogen bonds with the protons of TMA^+^, or cation–dipole interactions with the Cs^+^ cations. These results in solid state are consistent with the ^1^H NMR spectroscopic results recorded in solution, namely that accommodating either a quaternary ammonium guest or two alkali cations drove all the methyl units to reside outside. The cavity of the cage in (Cs^+^)_2_⊂T1·2NTf_2_^−^ also encapsulates four water molecules. Each of the four water molecules forms one O–Cs bond with each of the two Cs^+^ cations simultaneously. These water molecules act as glues between the two cationic guests, helping the latter suppress Coulombic repulsion. Attempts to obtain single crystals of the binary complexes such as Na^+^⊂T1·BArF^−^ were unsuccessful.

## Conclusions

To sum up, we successfully self-assembled a tetrahedral cage *via* imine condensation. The methyl units in the trisformyl building blocks play an important role in the cage formation, by introducing steric hindrance to afford a twisted conformation that favors the occurrence of intramolecular CH⋯π driving forces. Within the framework, each of the twelve *meta*-methyl-pyridyl units undergoes pivoting motion, affording the cage multiple conformations with a different number of methyl units pointing inwards/outwards. The “free” cage adopts a conformation where six methyl units point inward on average, as indicated by crystallography in solid state and ^1^H NMR spectroscopy in solution. In the presence of a guest, the cage can switch its conformation to increase the effective volume of its cavity, by moving more methyl units outside and placing more pyridyl nitrogen atoms inside. The latter units, which are Lewis basic binding sites, endow the cage with the ability to accommodate Lewis acidic guests. On average, after accommodating one alkali cation such as Na^+^, nine of the twelve nitrogen atoms pointed inwards to form cation–dipole interactions with the guest. The effective volume of the cage cavity can be further increased, by adopting a conformation in which all twelve nitrogen atoms point inwards. This conformation allows the cage to accommodate either one quaternary ammonium guest or two alkali cations simultaneously. This cage modulating its conformation to optimize guest binding improves our understanding of the mechanism of how biological hosts employ allosteric effects to accomplish a variety of tasks, such as cargo loading and release within cells.

## Data availability

The data supporting this article have been included as part of the ESI.† The X-ray crystallographic coordinates for structures reported in this study have been deposited at the Cambridge Crystallographic Data Centre (CCDC), under deposition numbers 2395426, 2441591 and 2396911. These data can be obtained free of charge from The Cambridge Crystallographic Data Centre *via*https://www.ccdc.cam.ac.uk/structures.

## Author contributions

H. L. and H. T. conceived the concept. H. T. and Y. L. performed the experiments and analyzed the data. Y. Q. offered a variety of alkali cations. J. L. tested and refined the single crystals. H. C. provided beautiful image models. The manuscript was written through contributions of all authors. All authors have given approval to the final version of the manuscript.

## Conflicts of interest

There are no conflicts to declare.

## Supplementary Material

SC-OLF-D5SC01474C-s001

SC-OLF-D5SC01474C-s002
