# Supplementary material for: A conformationally adaptable tetrahedral cage with different guest encapsulation models
Source: Chem Sci. 2025 May 9;16(24):10867–73. doi: 10.1039/d5sc01474c (PMC12082569; doi:10.1039/d5sc01474c)
Supplement: SC-016-D5SC01474C-s001 [file SC-016-D5SC01474C-s001.pdf]

Supplementary Information (SI) for Chemical Science.
This journal is © The Royal Society of Chemistry 2025

Supplementary Information

A Conformationally Adaptable Tetrahedral Cage with Different Guest Encapsulation Models

Hua Tang,[a] Yuyang Lu,[a] Yongwei Qian,[a] Chenqi Ge,[a] Jiyong Liu,[a] Hongliang Chen,*[a,b] and Hao Li*[a,b]

Supplementary Information Contents

Supplementary Information Contents 2
1. Materials and general methods S1
2. Synthesis of **F1** and Cage **T1**...S2
3. NMR & MS Spectroscopic Characterization S4
4. Synthesis of **F2** and triangular prism **2p**..S10
5. **T1** for cations recognition S13
6. X-ray crystallography S61
7. References...S67

1. Materials and general methods

All reagents and solvents were purchased from commercial sources and used without further purification. Manipulations were performed under a normal atmosphere unless otherwise indicated. Nuclear magnetic resonance (NMR) spectra were recorded at ambient temperature using Bruker AVANCE III 400/500 and Agilent DD2 600 spectrometers, with working frequencies of 400/500/600 and 100/125/150 MHz for ^{1}H and ^{13}C, respectively. Chemical shifts are reported in ppm relative to the residual internal non-deuterated solvent signals (DMSO: δ = 2.50 ppm, $CDCl_3$: δ = 7.26 ppm, CD_3CN δ = 1.94 ppm). High-resolution mass spectra (HRMS) were performed on a Quadruple-Time-Of-flight Q-TOF mass spectrometer. X-ray crystallographic data were collected on a Bruker D8 Venture diffractometer. Ultraviolet and Visible (UV-vis) spectra were recorded on SHIMADAZU UV-2600. All the reactions that require heating used oil bath. Structural assignments were made with additional information from gCOSY, gHSQC, gHMBC, NOESY experiments.

2. Synthesis of F1 and Cage T1

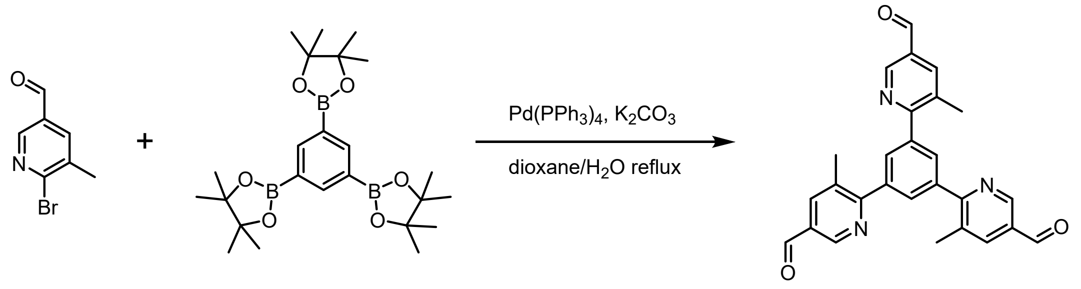

Fig. S1. Synthesis of the **F1**.

Precursors **F1**: 6-bromo-5-methylnicotinaldehyde (414.6 mg, 2.1 mmol), 1,3,5-Tris(4,4,5,5-tetramethyl-1,3,2-dioxaborolan-2-yl)benzene (236.7 mg, 0.5 mmol) and K_2CO_3 (1.40 g, 10.1 mmol) were dissolved in a solution containing dioxane (10 mL) and water (2 mL). The reaction mixture was degassed, after which $Pd(PPh_3)_4$ (55.4 mg, 0.05 mmol) was added. The reaction mixture was stirred at 90 °C under the protection of nitrogen for 48 h. After cooling to room temperature, the residue was poured into saturated brine, following by extracting with EtOAc for three times. The combined organic layers were dried over anhydrous Na_2SO_4. The solvent was removed under reduced pressure and the residue was purified by column chromatography (DCM/MeOH (100:1 to 40:1); silica gel, 200-300 mesh), which yielded pure **F1** (210.6mg; yield = 93.2%). **^{1}H NMR** (400 MHz, $CDCl_3$): δ 10.14 (s, 3H), 8.99 (d, J = 2.0 Hz, 3H), 8.10 (dd, J = 2.0, 0.9 Hz, 3H), 7.92 (s, 3H), 2.56 (s, 9H). **^{13}C NMR** (100 MHz, $CDCl_3$): δ 190.8, 162.6, 149.5, 139.9, 138.4, 132.0, 130.3, 130.3, 20.4. **HRMS-ESI**: m/z calcd for $[M+H]^+$ $C_{27}H_{22}N_3O_3^+$, 436.1661; found 436.1657.

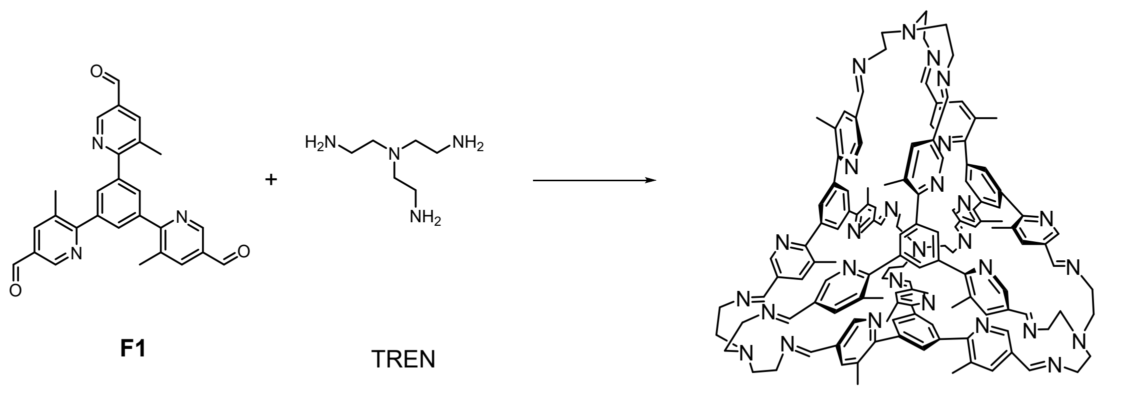

Fig. S2. Synthesis of the **T1**.

Cage **T1**: The tetrahedral cage **T1** was prepared by condensing **F1** (65.8 mg, 0.15 mmol, 1 eq.) and tris(2-aminoethyl)amine (TREN) (22.1 mg, 0.15 mmol, 1 eq.) in $CHCl_3$ or $CDCl_3$ (10 mL). The mixture was stirred at room temperature for 30 h to obtain **T1** as the major product, accompanied with oligomeric and polymeric byproducts obtained as minor producs in the ^{1}H NMR spectrum. The assembly yield was calculated to be 88% by integrating and comparing the corresponding resonances corresponding to the precursr and cage product relative to an internal standard whose concentration remained constant before and after the self-assembly. Purer sample of **T1** was obtained by diffusion of isopropyl ether into the solution of **T1** in $CHCl_3$, leading to precipitation of the oligomeric and polymeric byproducts, which were removed via filtration. The filtrate was collected, which underwent another round of diffusion of isopropyl ether, leading to precipitation of **T1**. The latter was then washed with MeCN and dried in vaccum, yielding pure solid-state **T1** (45.3 mg, yield = 56.8%). **^{1}H NMR** (400 MHz, $CDCl_3$): δ 8.16 (d, J = 2.0 Hz, 1H), 7.77 (s, 1H), 7.59 (s, 1H), 7.02 (s, 1H), 3.82 (s, 1H), 3.42 (s, 1H), 3.05 (s, 1H), 2.61 (s, 1H), 2.02 (s, 3H). **^{13}C NMR** (100 MHz, $CDCl_3$):δ 160.5, 159.1, 146.6, 140.1, 136.7, 131.8, 130.3, 129.4, 59.9, 55.7, 19.7. **HRMS-ESI**: m/z calcd for $[M+Na]^+$ $C_{132}H_{132}N_{28}Na^+$, 2132.1087; found 2132.1084.

3. NMR & MS Spectroscopic Characterization

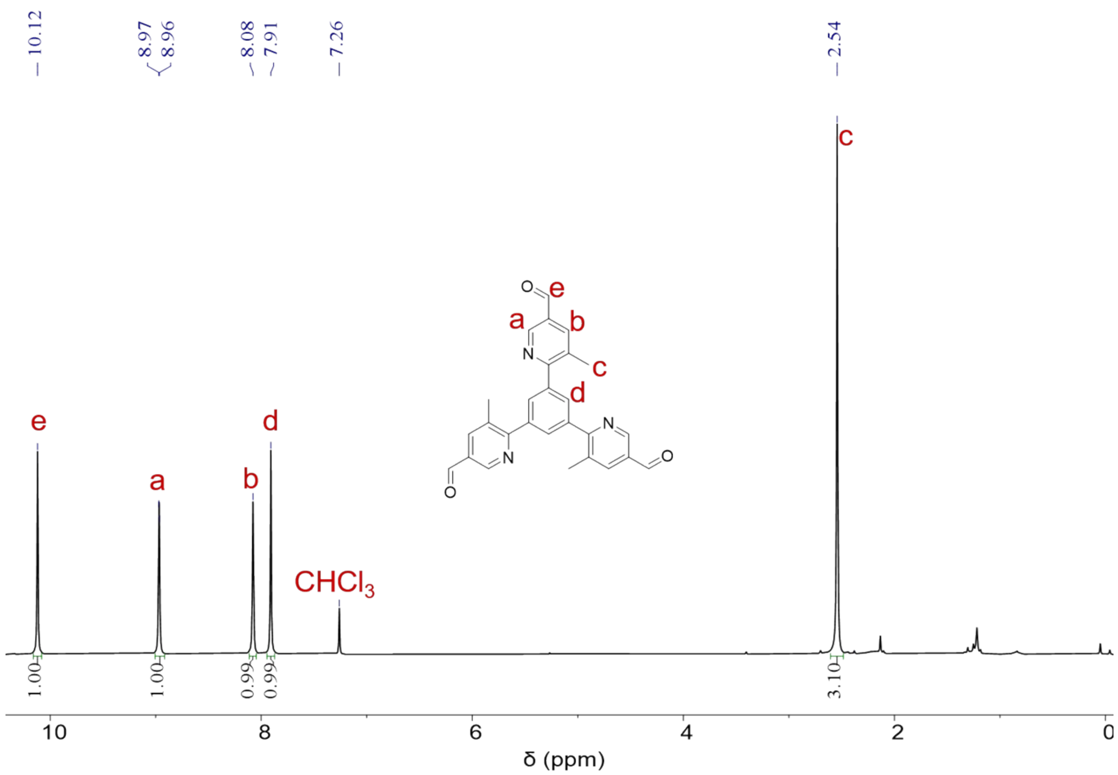

Fig. S3. ^{1}H NMR spectrum of **F1** (400 MHz, $CDCl_3$, 298 K).

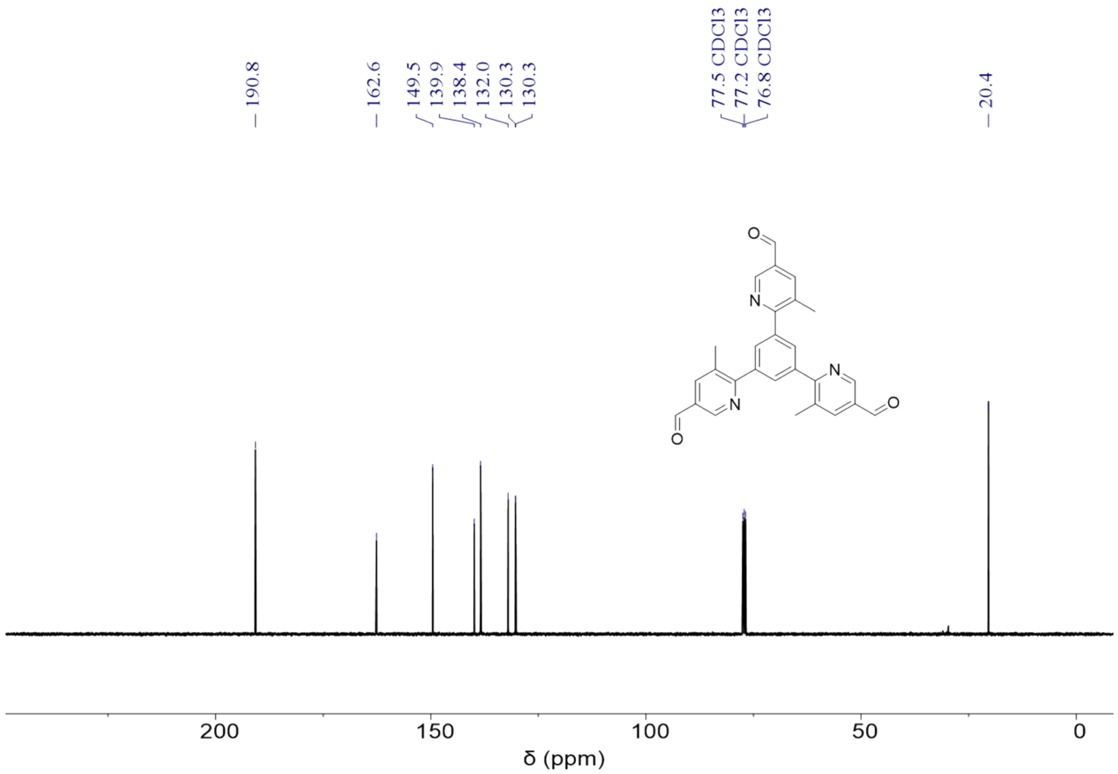

Fig. S4. ^{13}C NMR spectrum of **F1** (100 MHz, $CDCl_3$, 298 K).

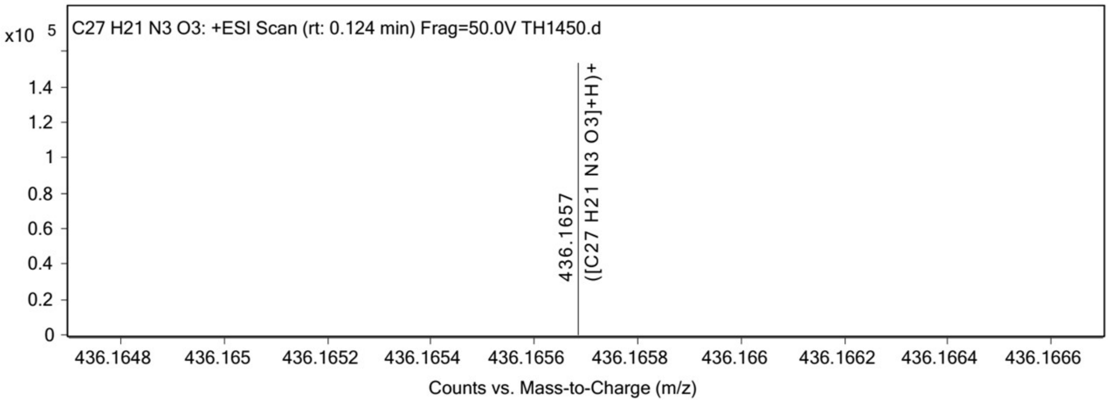

Fig. S5. ESI-HRMS spectrum of **F1**.

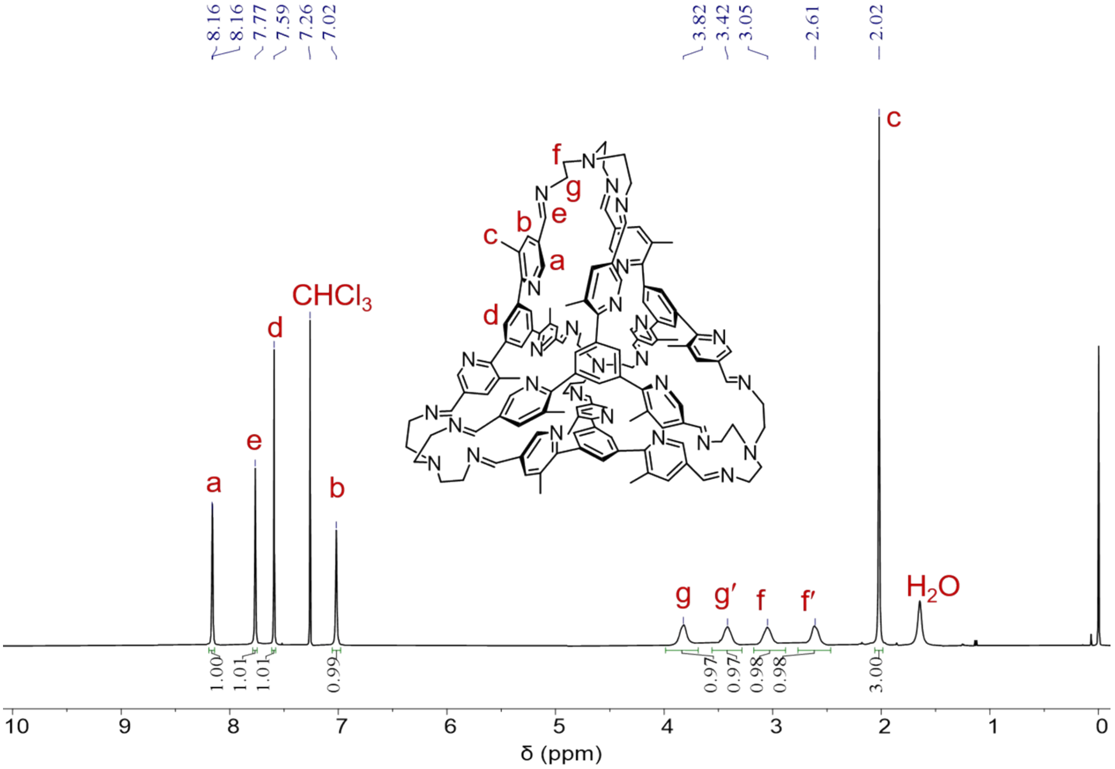

Fig. S6. ^{1}H NMR spectrum of **T1** (400 MHz, $CDCl_3$, 298 K).

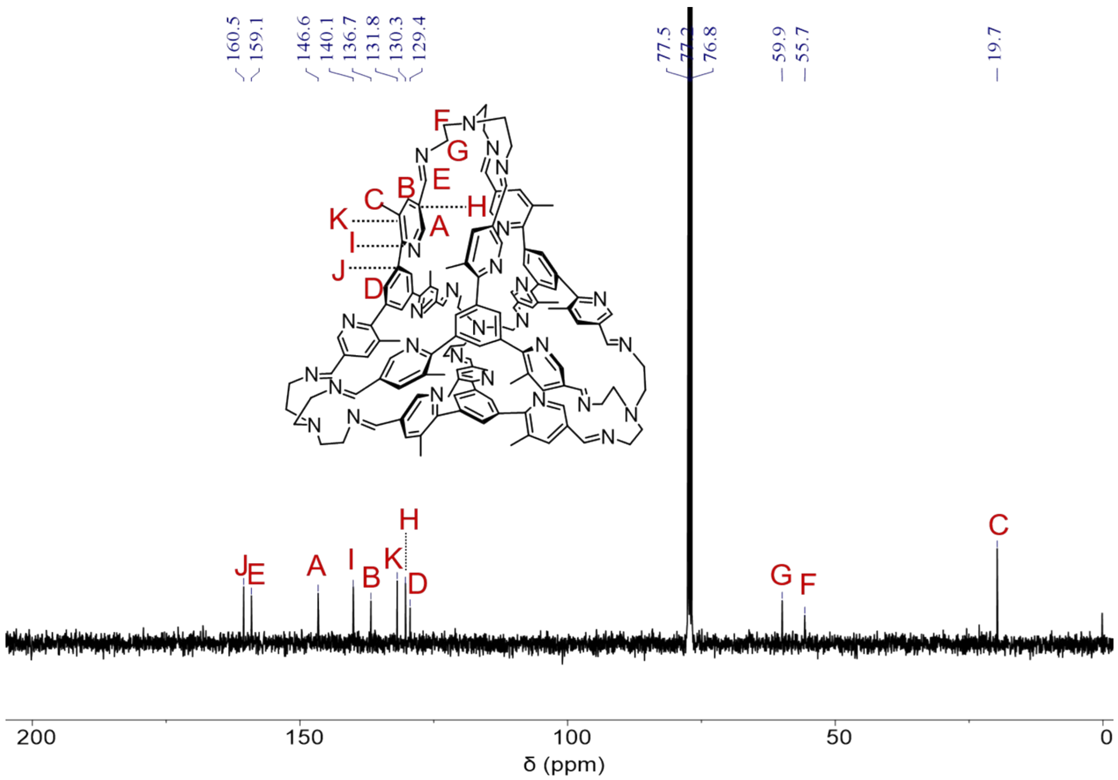

Fig. S7. ^{13}C NMR spectrum of **T1** (100 MHz, $CDCl_3$, 298 K).

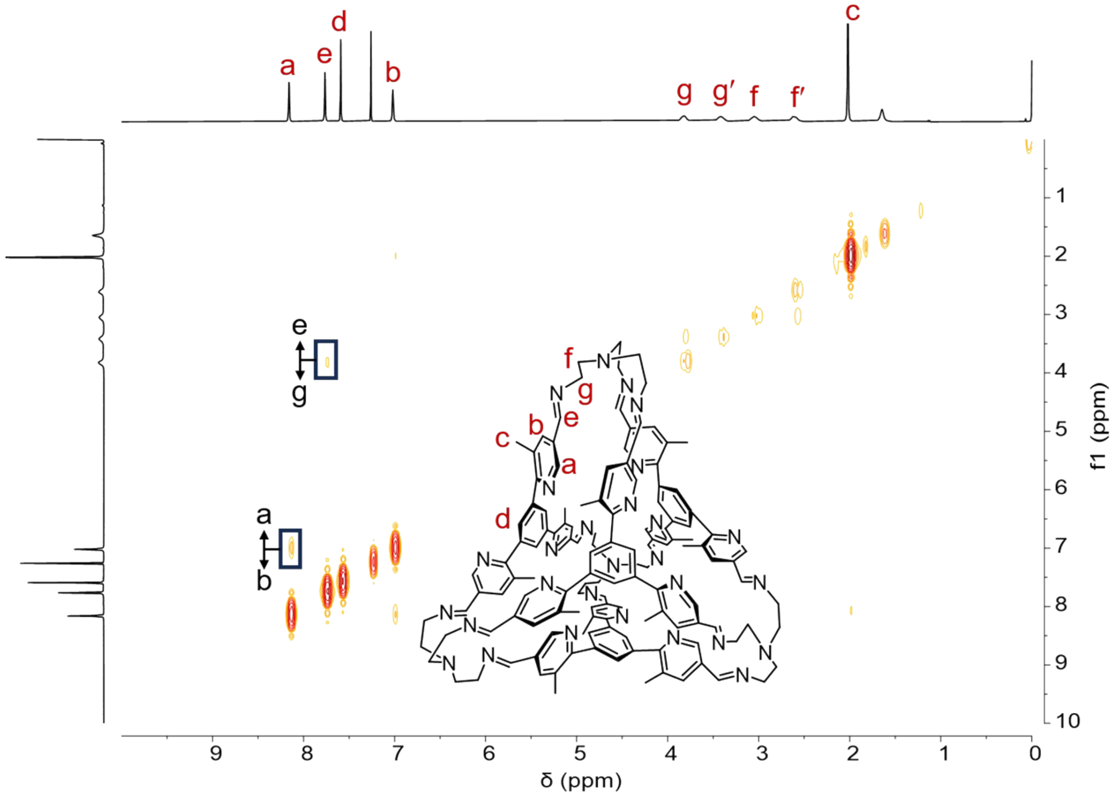

Fig. S8. ^1H-^1H COSY NMR spectrum of **T1** (400 MHz, $CDCl_3$, 298 K). Key correlation peaks are labeled in the spectrum.

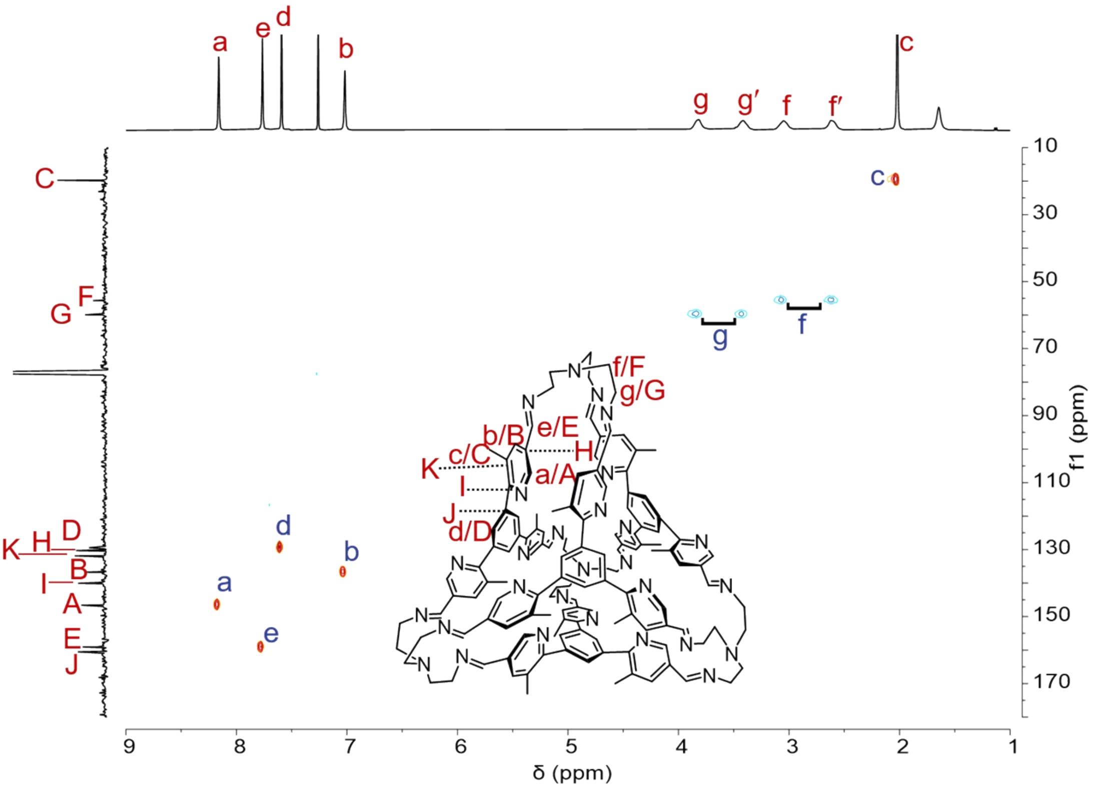

Fig. S9. ^{1}H-^{13}C HSQC NMR spectrum of **T1** (400 MHz, $CDCl_3$, 298 K). Key correlation peaks are labeled in the spectrum.

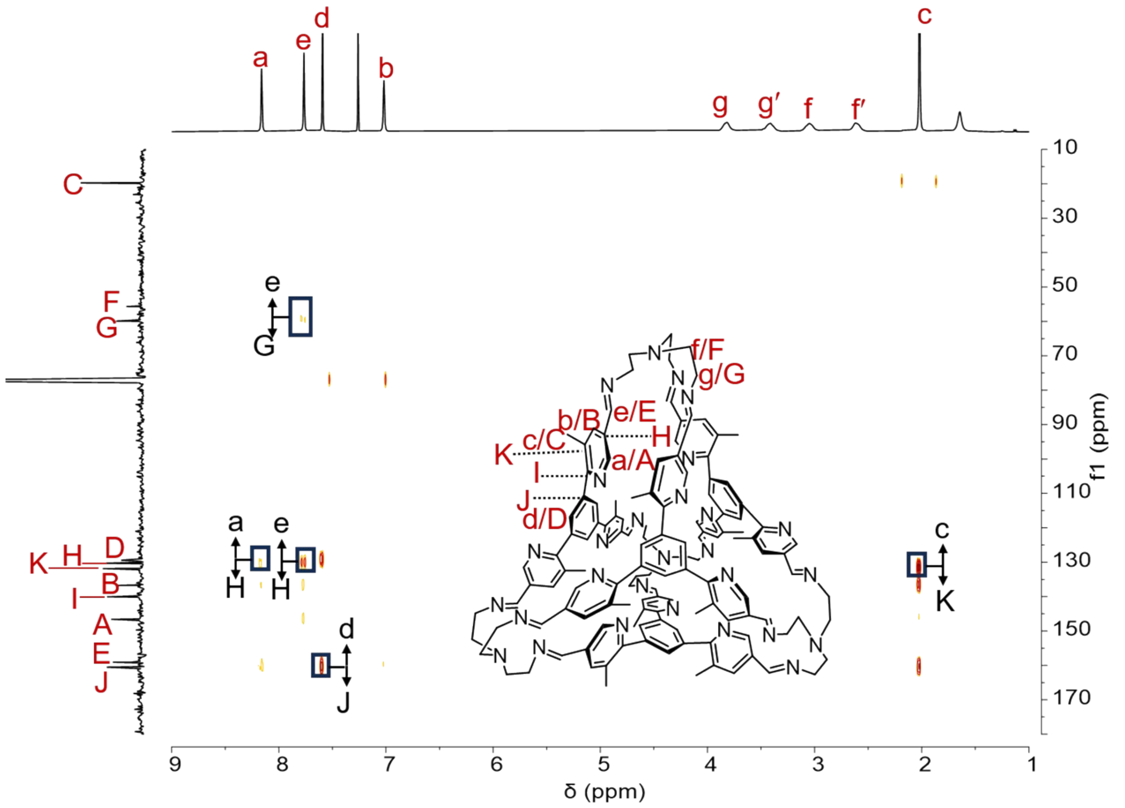

Fig. S10. ^{1}H-^{13}C HMBC NMR spectrum of **T1** (400 MHz, $CDCl_3$, 298 K). Key correlation peaks are labeled in the spectrum.

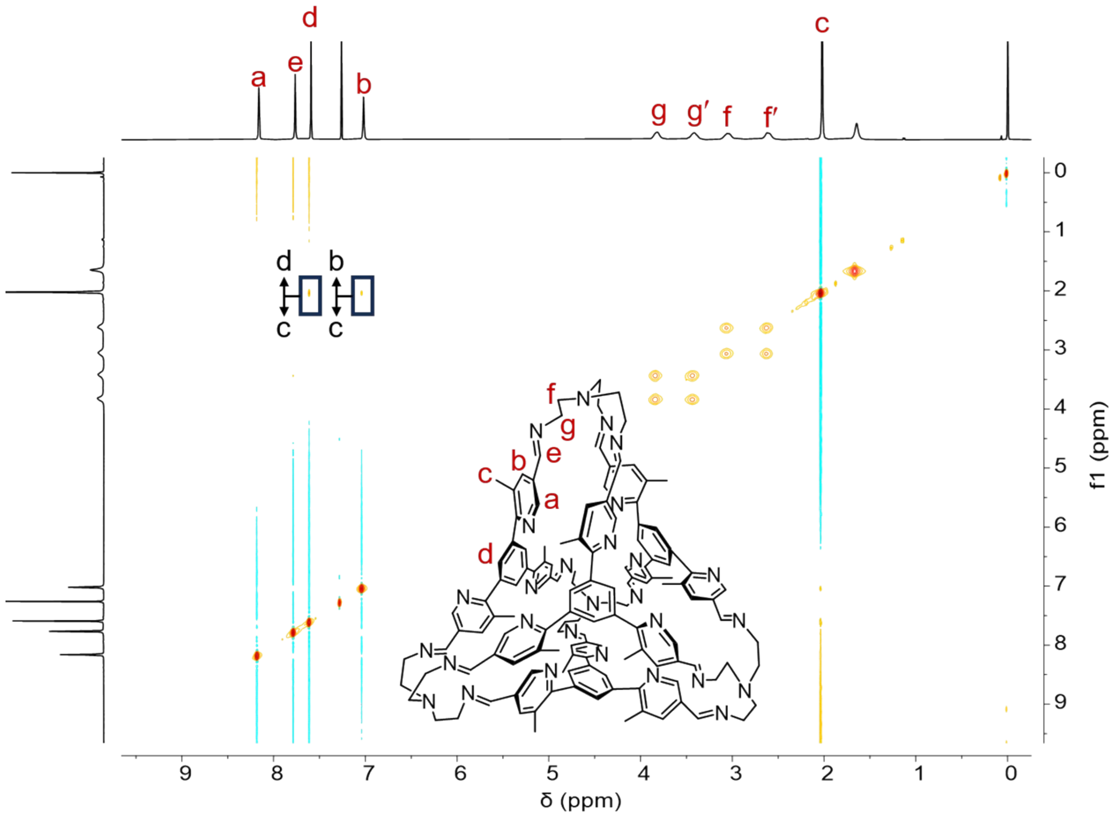

Fig. S11. ^{1}H-^{1}H NOESY spectrum of **T1** (400 MHz, $CDCl_3$, 298 K). Key correlation peaks are labeled in the spectrum.

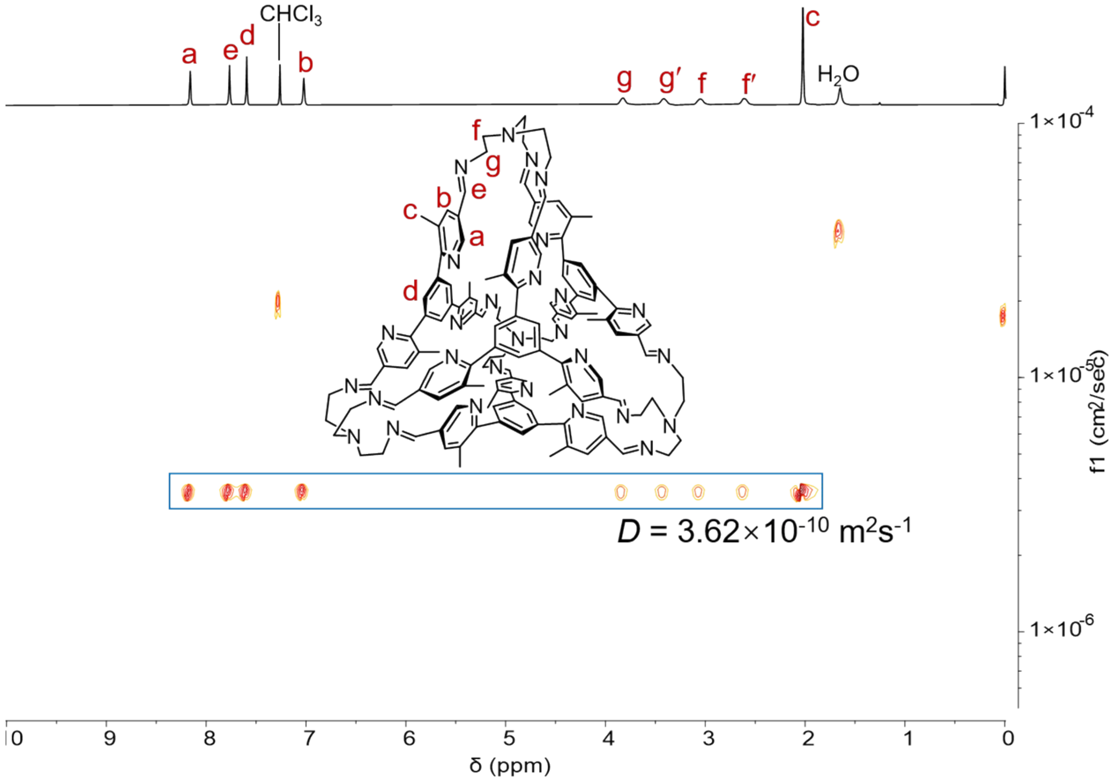

Fig. S12. ^{1}H-^{1}H DOSY spectrum of **T1** (500 MHz, $CDCl_3$, 298 K).

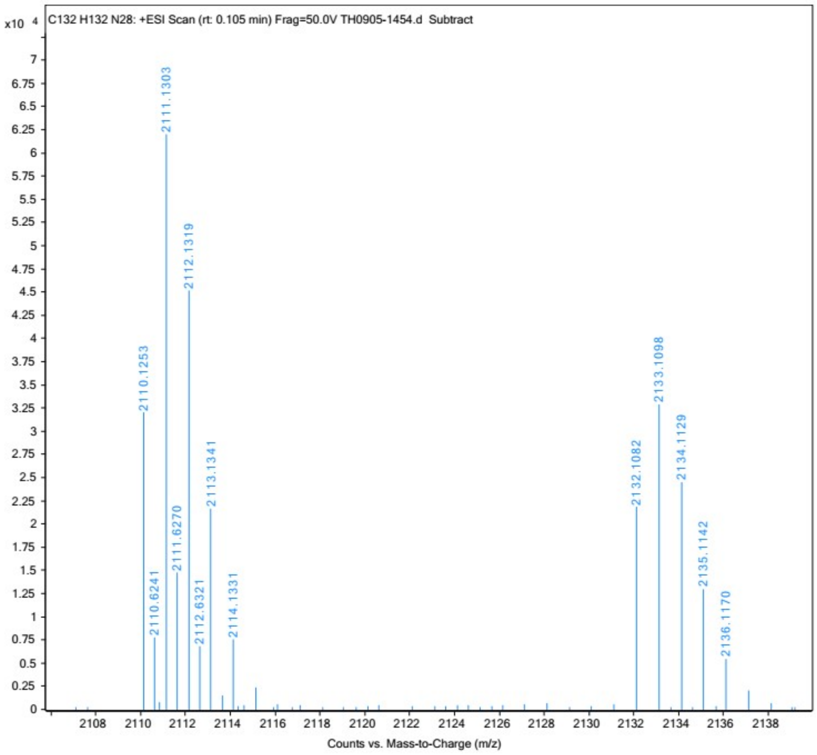

Fig. S13. ESI-HRMS of **T1**. The signals labelled in the spectra correspond to molecular cations bearing one H^+ or Na^+ respectively. ESI-HRMS *m/z* calcd for $[M+H]^+$ $C_{132}H_{133}N_{28}^+$, 2110.1263; found 2110.1253; *m/z* calcd for $[M+Na]^+$ $C_{132}H_{132}N_{28}Na^+$, 2132.1082; found 2132.1082.

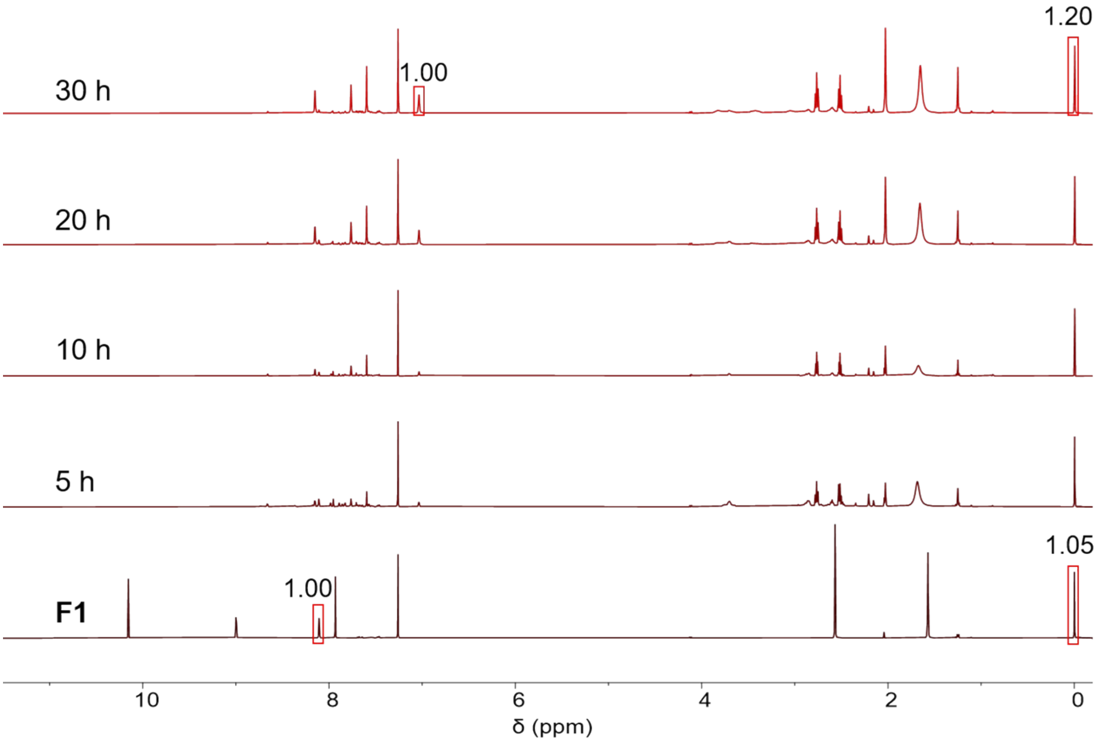

Fig. S14. ^1H NMR spectrum (400 MHz, $CDCl_3$, 298 K) of **F1** before (bottom) and after adding TREN for at 5 h, 10 h, 20 h and 30 h (top). By using the resonance corresponding to TMS as the internal standard, the yield of **T1** was calculated to be 88% by integrating and comparing the resonances corresponding to the precursor and product.

4. Synthesis of F2 and triangular prism 2p

The tris-pyridylaledhyde **F2** was synthesized via a similar precedure as **F1**, except that 2-bromopyridine-5-carbaldehyde was used as one of the precursors. After suzuki reaction, **F2** was precipitated from the solution and obtained via filtration.

By condensing **F2** and TREN in $CDCl_3$, a triangular prism **2p** was self-assembled after stirring the reaction mixture at room temperature for 12h, instead of a putative tetrahedral cage. Because **F2** has an extremely low solubility, **2p** was self-assembled in a very low concentrated solution. As a result, we could not obtain its ^{13}C NMR spectrum.

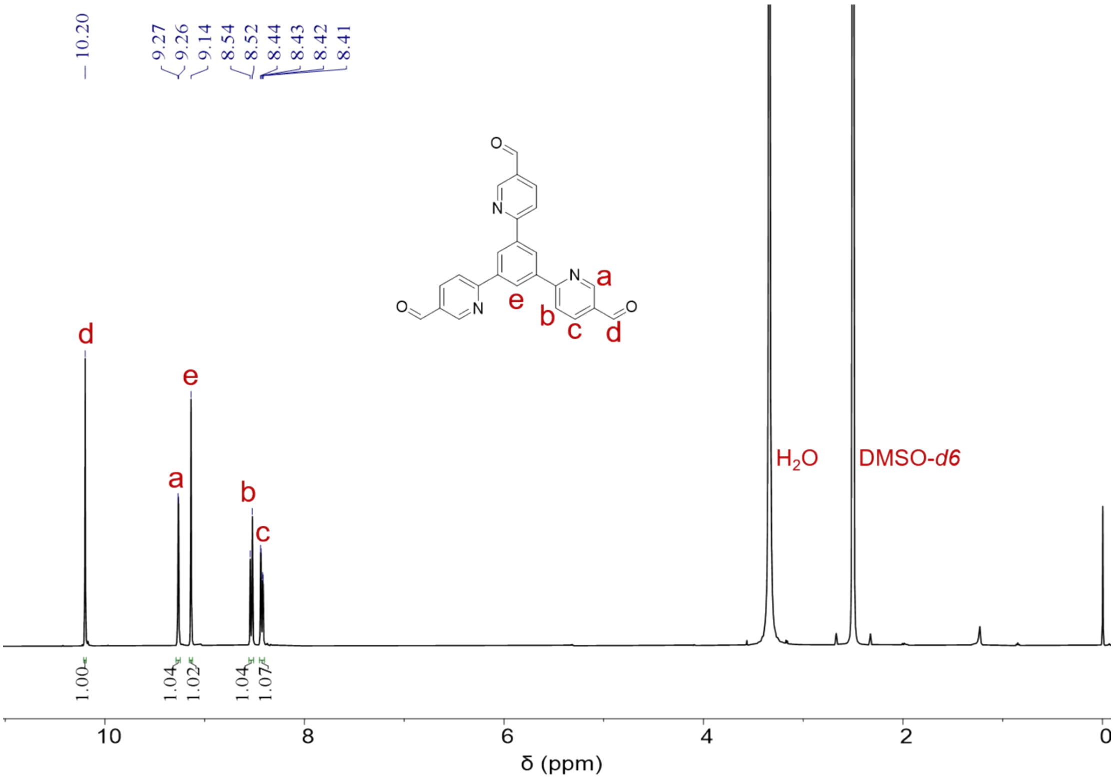

Fig. S15. ^{1}H NMR spectrum of **F2** (400 MHz, DMSO-d_6, 298 K).

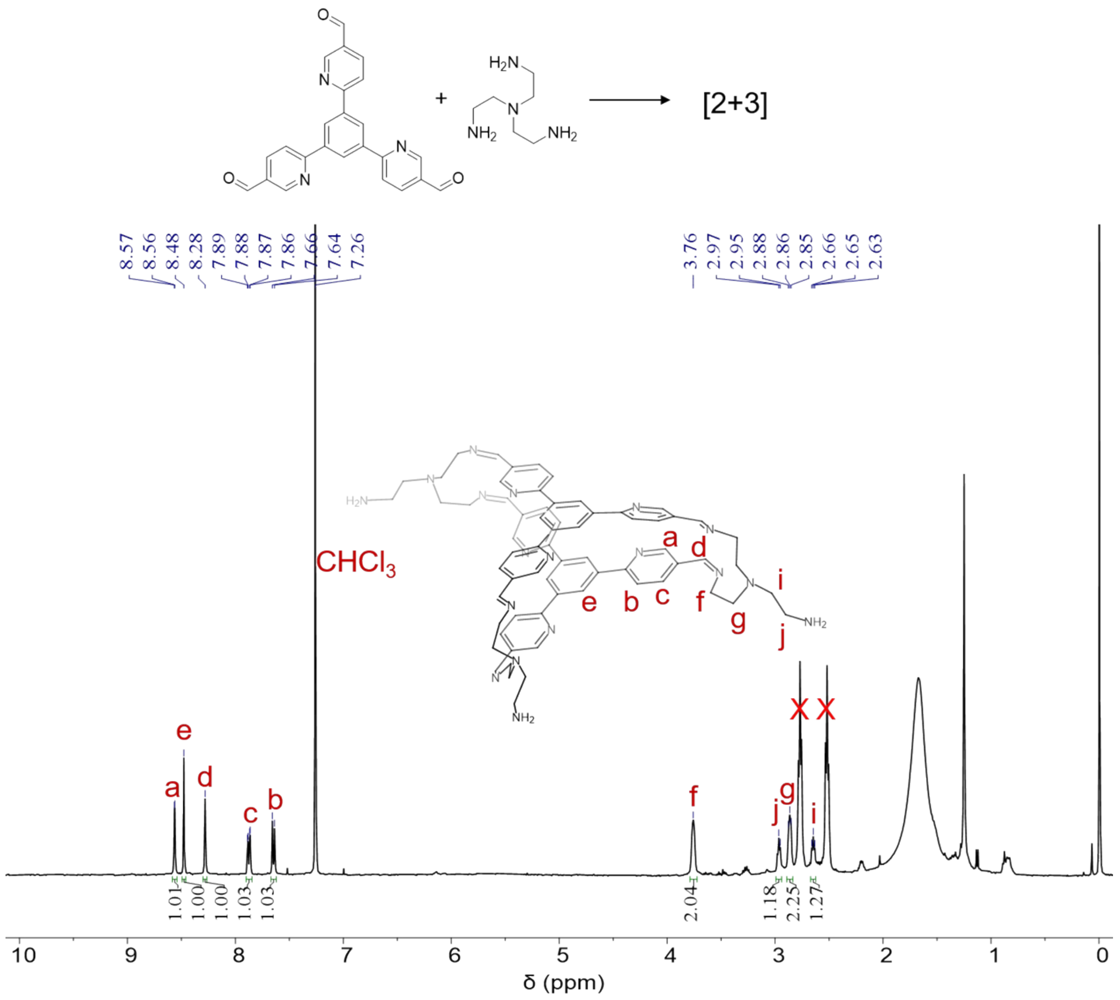

Fig. S16. ^{1}H NMR spectrum of **2p** (400 MHz, $CDCl_3$, 298 K).

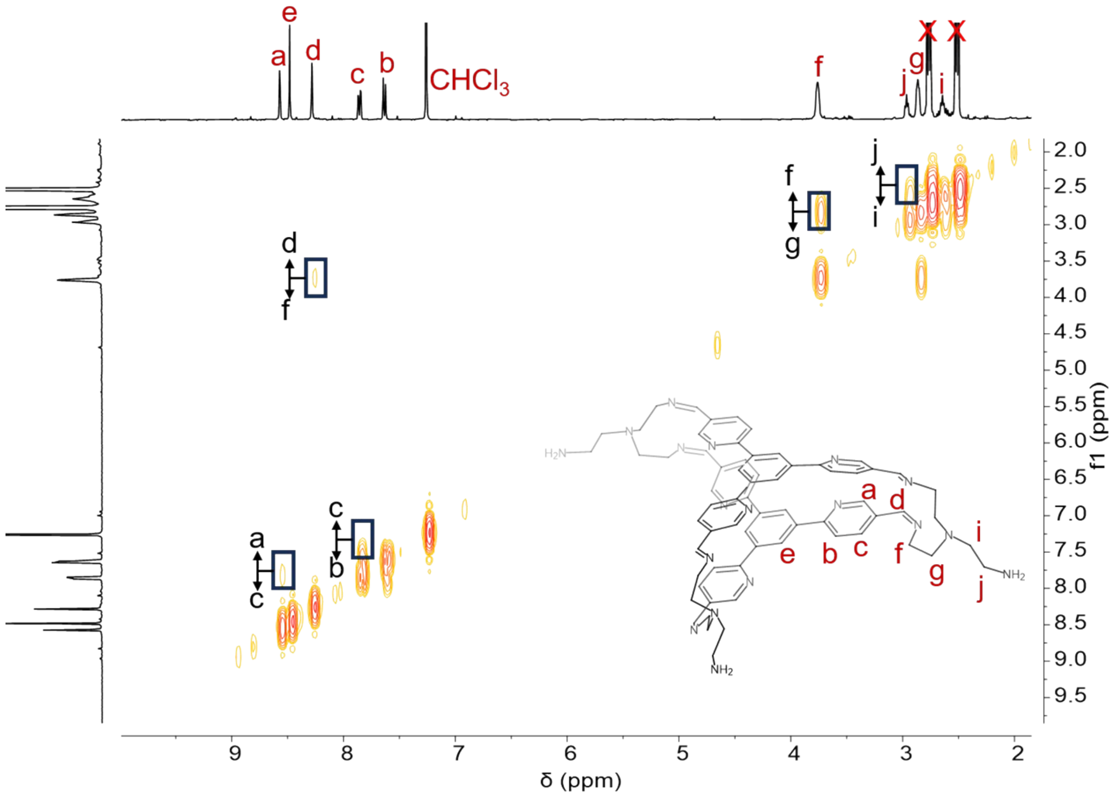

Fig. S17. ^{1}H-^{1}H COSY NMR spectrum of **2p** (400 MHz, $CDCl_3$, 298 K). Key correlation peaks are labeled in the spectrum.

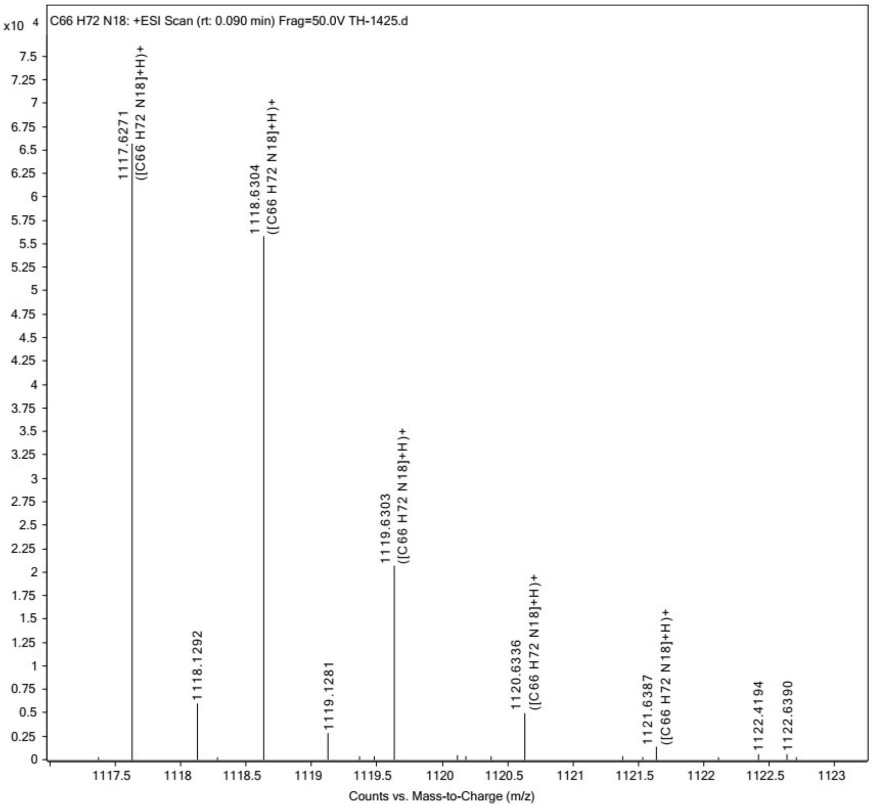

Fig. S18. ESI-HRMS of **2p**. ESI-HRMS *m*/*z* calcd for $[M+H]^+$ $C_{66}H_{73}N_{18}^+$, 1117.6260; found 1117.6271.

5. Cage T1 for cations recognition

(1) TMA$^+$ in cage **T1**

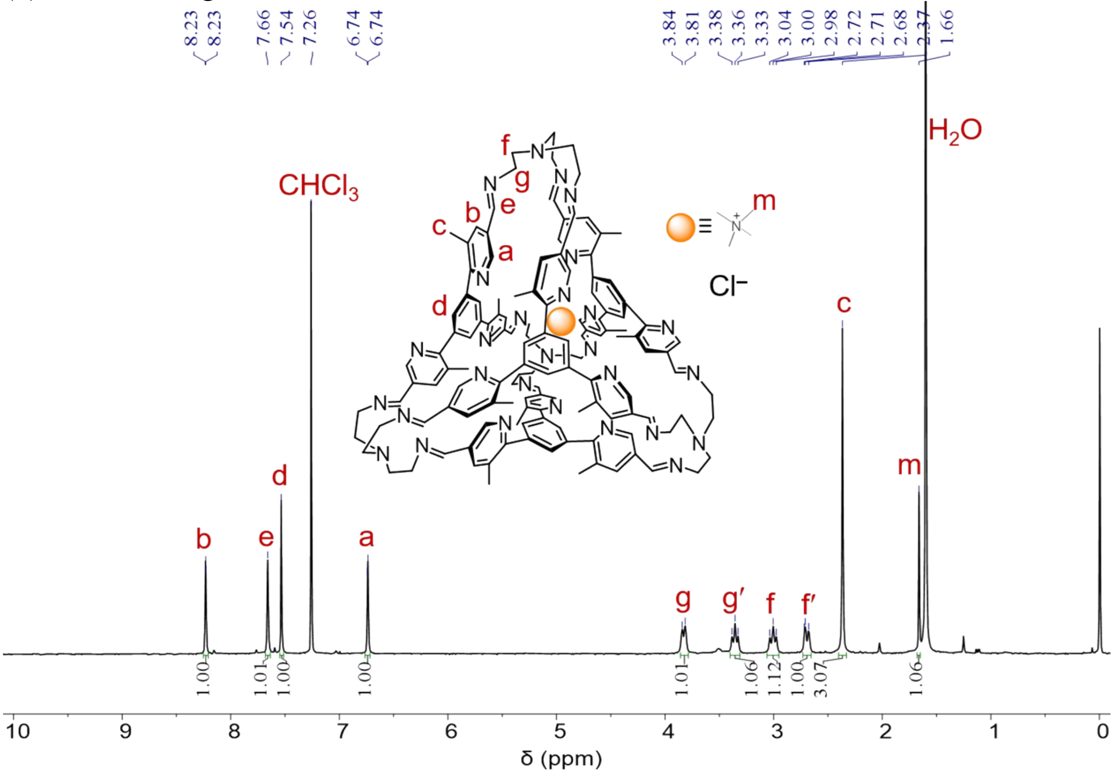

Fig. S19. ^{1}H NMR spectrum of TMA$^+$⊂**T1**•Cl$^-$ (400 MHz, CDCl$_3$, 298 K).

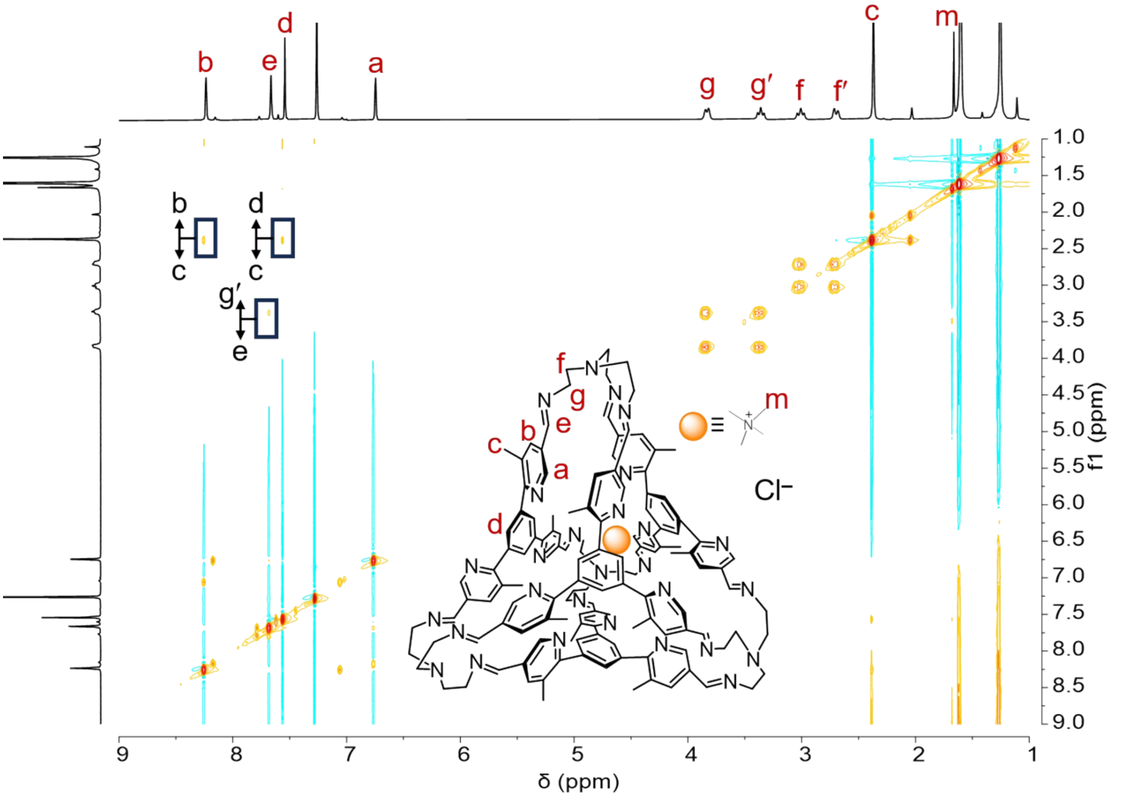

Fig. S20. ^{1}H-^{1}H NOESY spectrum of TMA$^+$⊂**T1**•Cl$^-$ (400 MHz, CDCl$_3$, 298 K). Key correlation peaks are labeled in the spectrum.

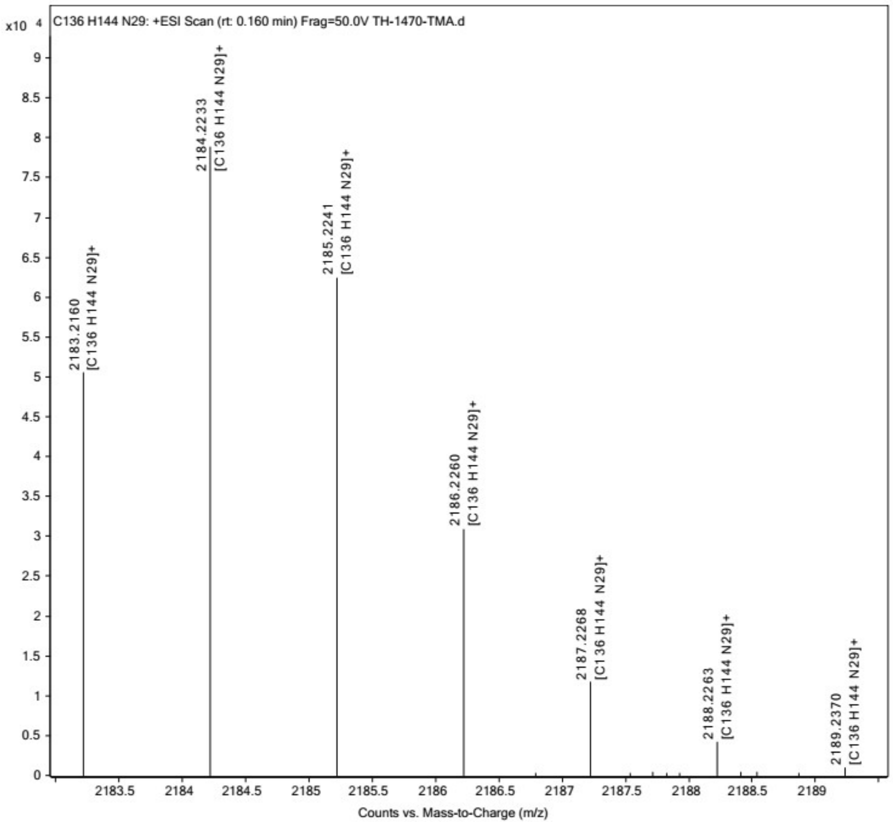

Fig. S21. ESI-HRMS of TMA$^+$⊂**T1**•Cl$^-$. ESI-HRMS *m/z* calcd for [**T1**+TMA]$^+$ $C_{136}H_{144}N_{29}^+$, 2183.2154; found 2183.2160.

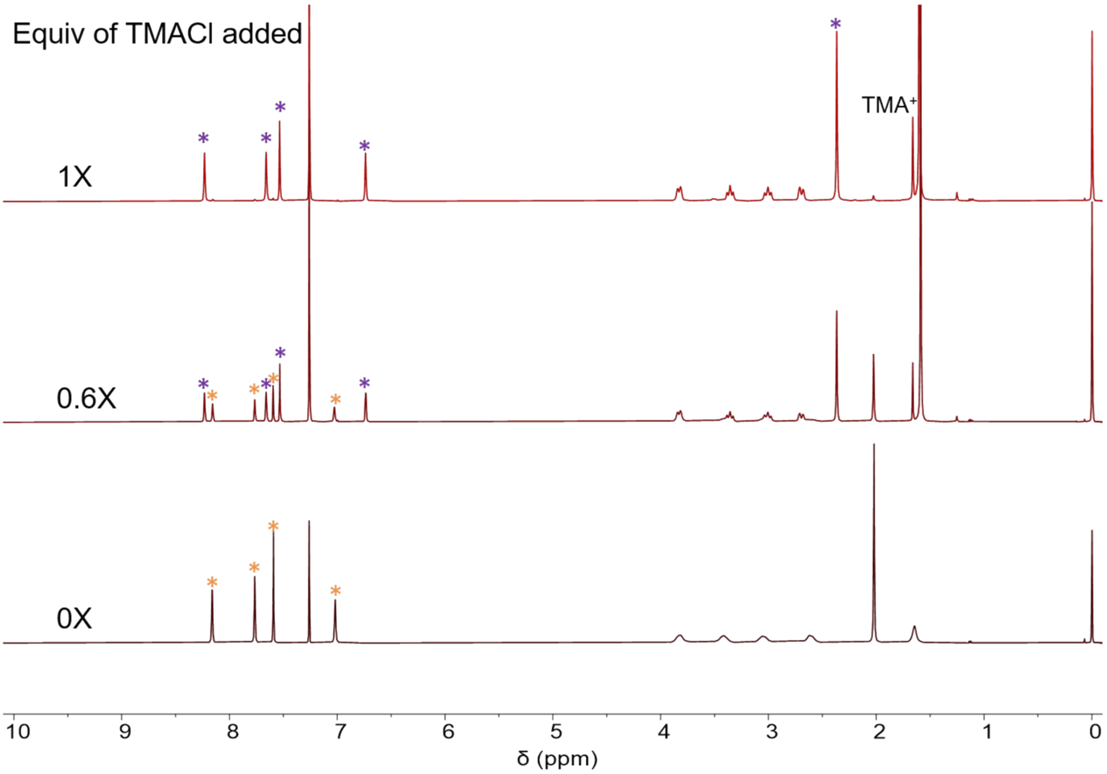

Fig. S22. ^{1}H NMR spectra of **T1** (400 MHz, $CDCl_3$, 298 K) in the presence of 0 (bottom), 0.6 (middle) and 1 (top) equiv. of TMA$^+$•Cl$^-$. In the spectra, the resonances labeled with orange and violet stars correspond to the “empty” cage and the complex, respectively.

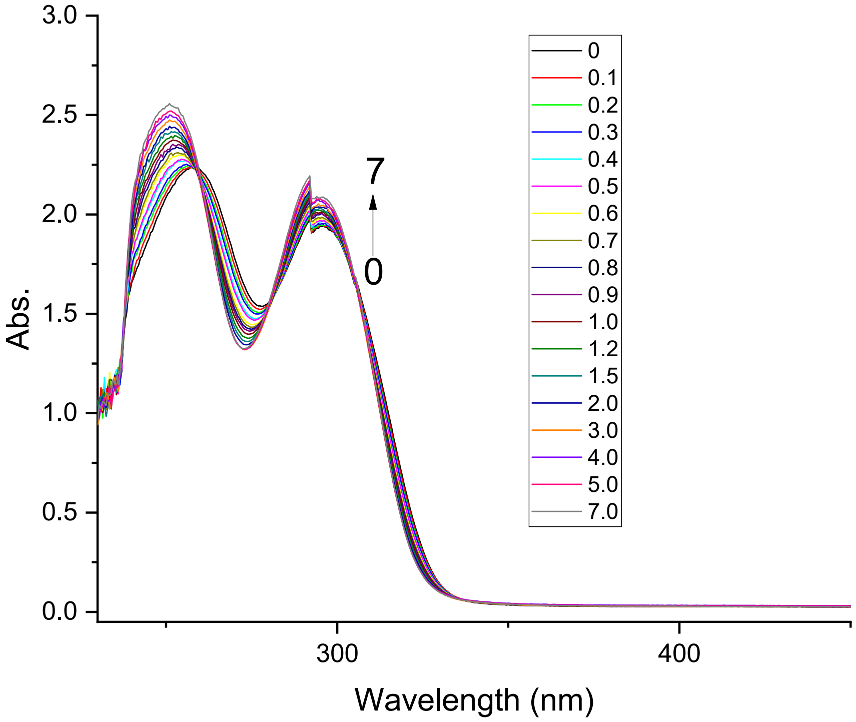

Fig. S23. UV-vis spectra of **T1** (1.61×10^{-5} M; 25 °C) after adding 0-7 equiv. of TMA^{+}•Cl^{-} in $CHCl_3$.

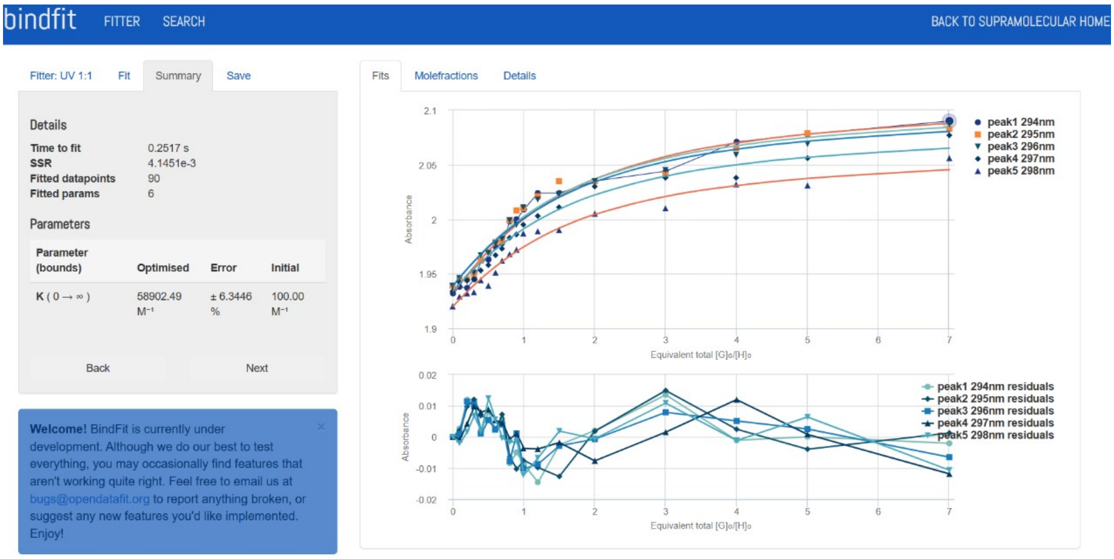

Fig. S24. Plots of absorption at 294 nm, 295nm, 296nm, 297nm, 298nm versus [TMA^{+}•Cl^{-}]/[**T1**]. The data were fitted to a 1:1 binding model, giving K_a= $(5.9\pm0.4)\times10^{4}$ M^{-1}. All plots were obtained from non-linear curve-fitting to a 1:1 binding model using the www.supramolecular.org web applet[1].

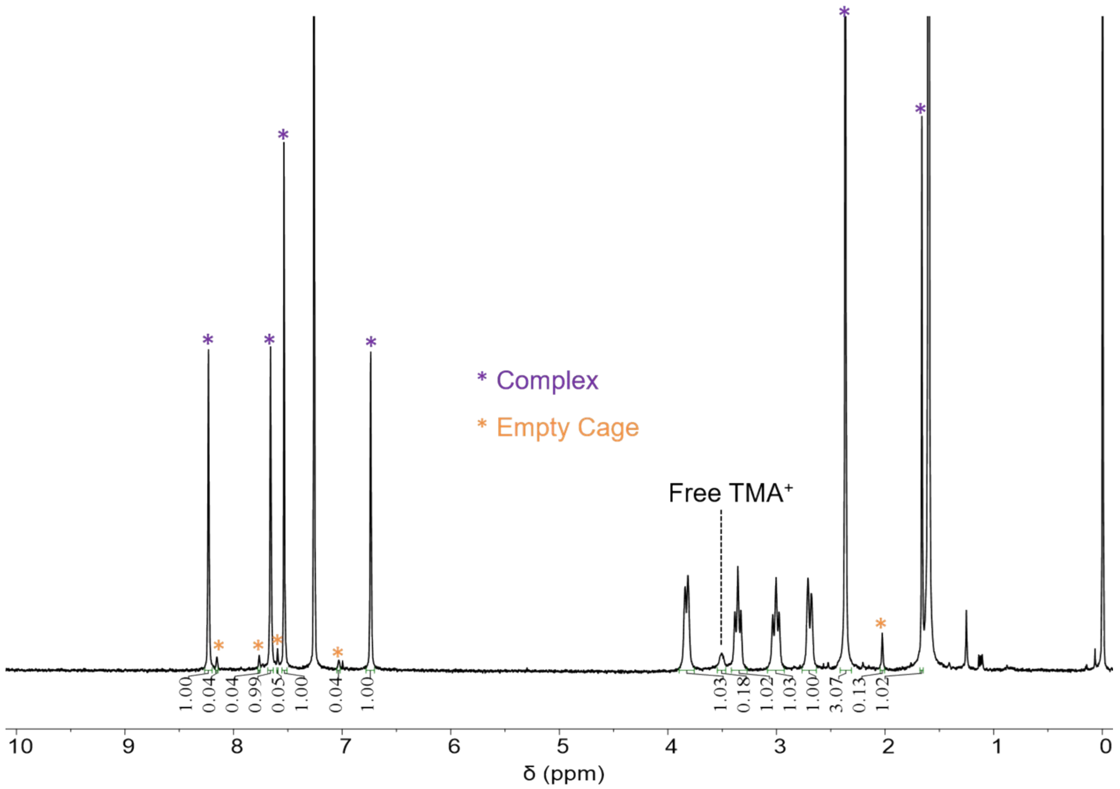

Fig. S25. ^{1}H NMR spectrum of **T1** in the presence of 1.1 equiv. of TMA^{+}•Cl^{-}. The resonances labelled with orange and violet stars corresponding the the empty cage and complex, respectively. The initial concentration of **T1** is 7.2 $\times 10^{-4}$ M. The binding constant *K*a is calculated to be $(2.0\pm0.06)\times10^{5}$ M^{-1} , by integrating and comparing the resonances corresponding to the complex, free guest and empty cage.

(2) Na^+ in cage **T1**

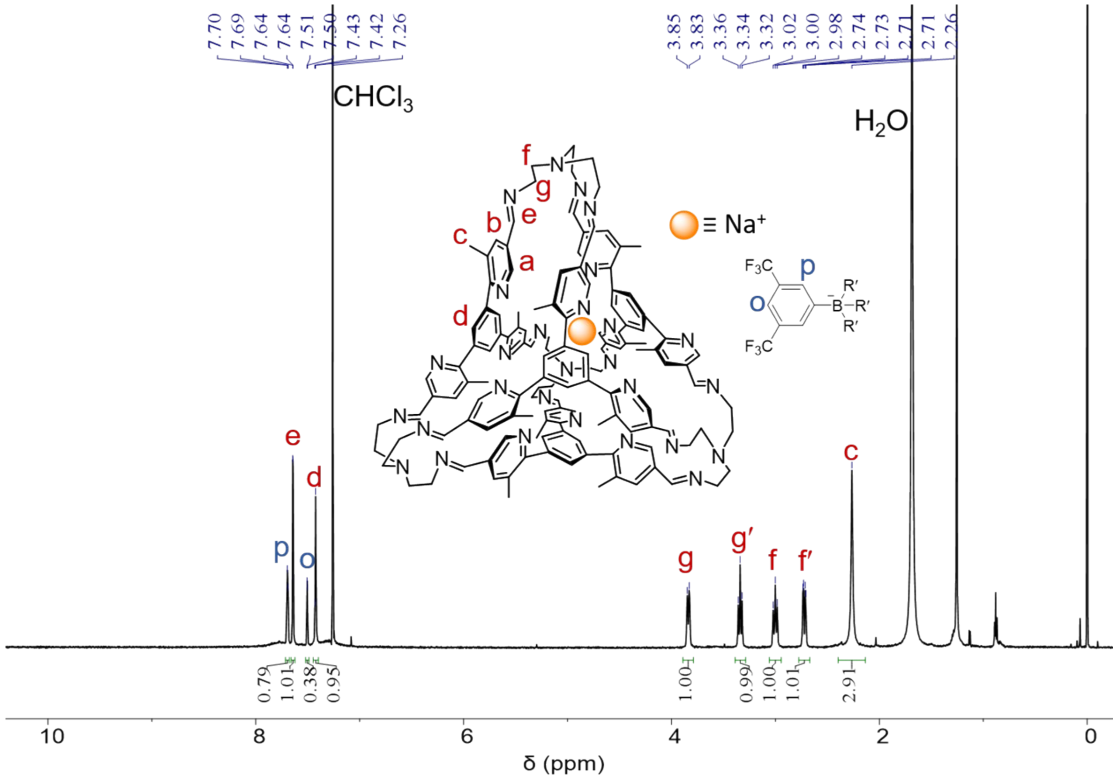

Fig. S26. ^{1}H NMR spectrum of $Na^+ \subset$**T1**•BArF⁻ (400 MHz, $CDCl_3$, 298 K). The resonances labelled with blue letters *p* and *o* correspond to the BArF⁻ counterion of Na^+.

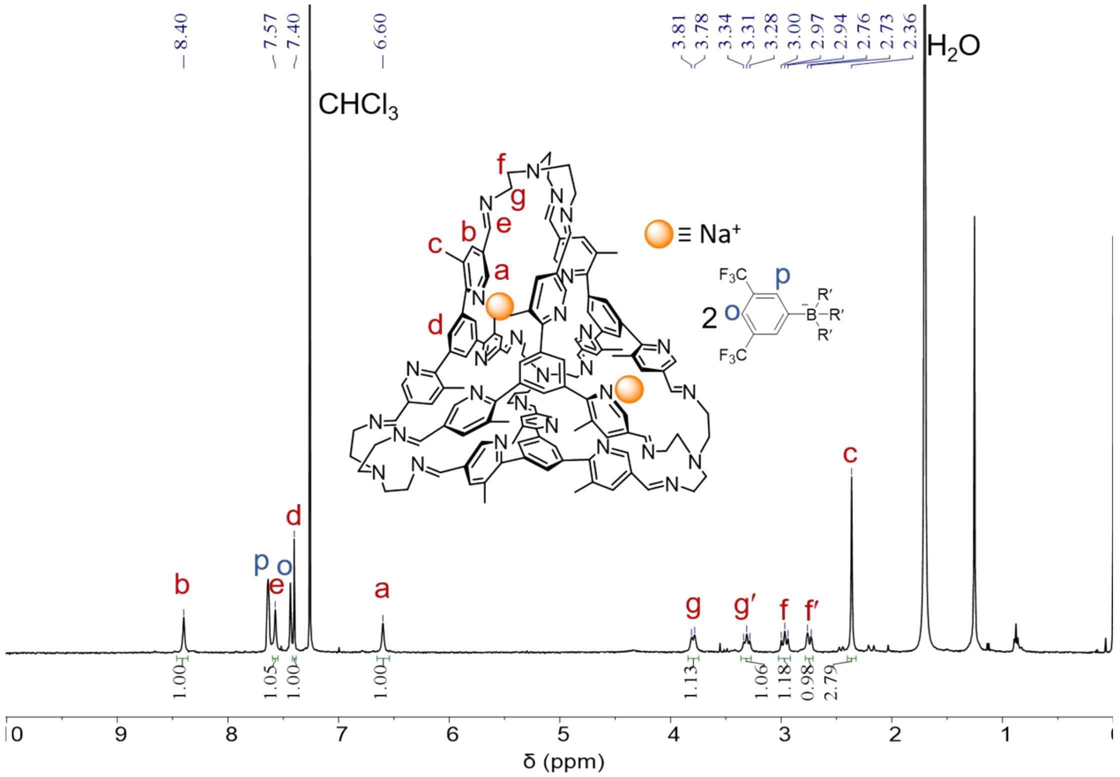

Fig. S27. ^{1}H NMR spectrum of $(Na^+)_2 \subset$**T1**•2BArF⁻ (400 MHz, $CDCl_3$, 298 K). The resonances labelled with blue letters *p* and *o* correspond to the BArF⁻ counterion of Na^+.

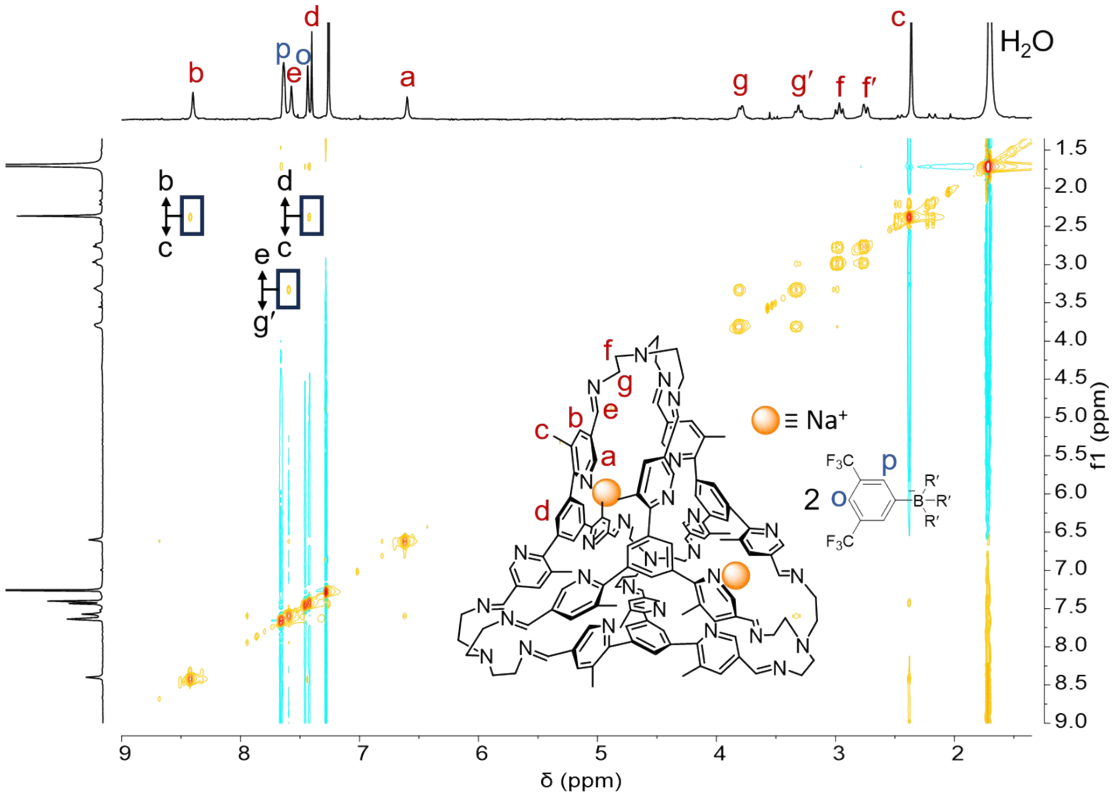

Fig. S28. ^{1}H-^{1}H NOESY spectrum of $(Na^+)_2{\subset}$**T1**•2BArF$^-$ (400 MHz, $CDCl_3$, 298 K). Key correlation peaks are labeled in the spectrum.

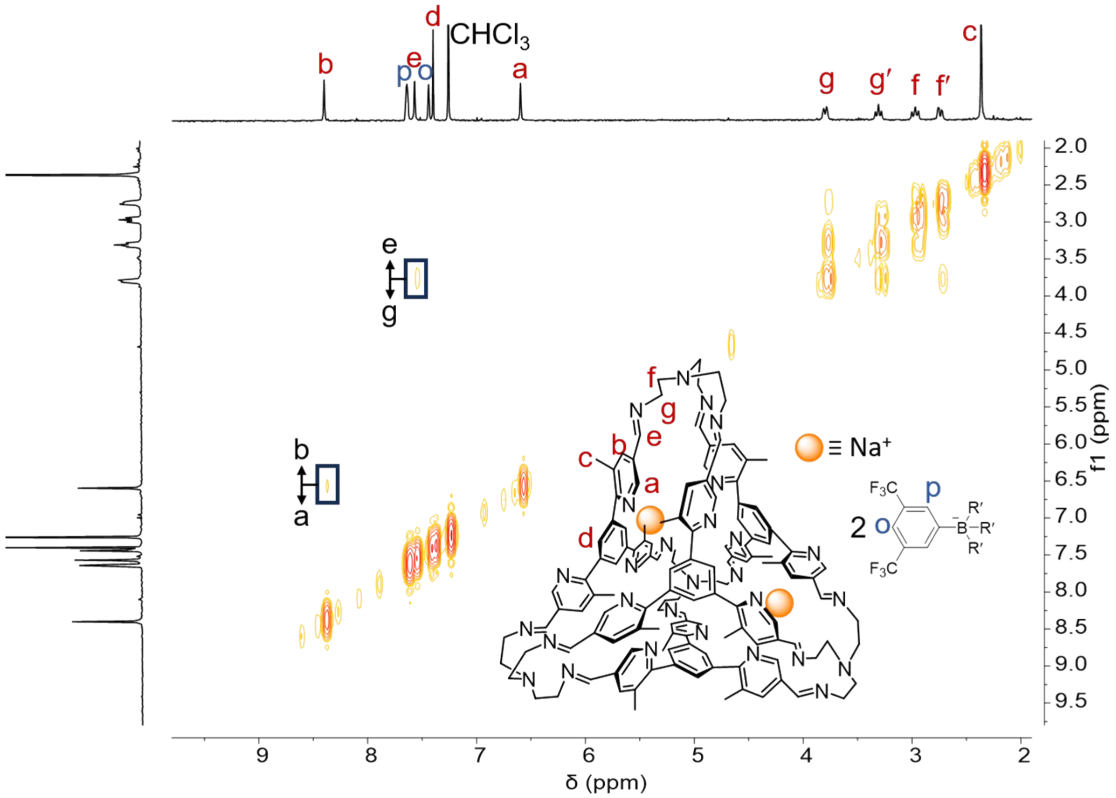

Fig. S29. ^{1}H-^{1}H COSY spectrum of $(Na^+)_2{\subset}$**T1**•2BArF$^-$ (400 MHz, $CDCl_3$, 298 K). Key correlation peaks are labeled in the spectrum.

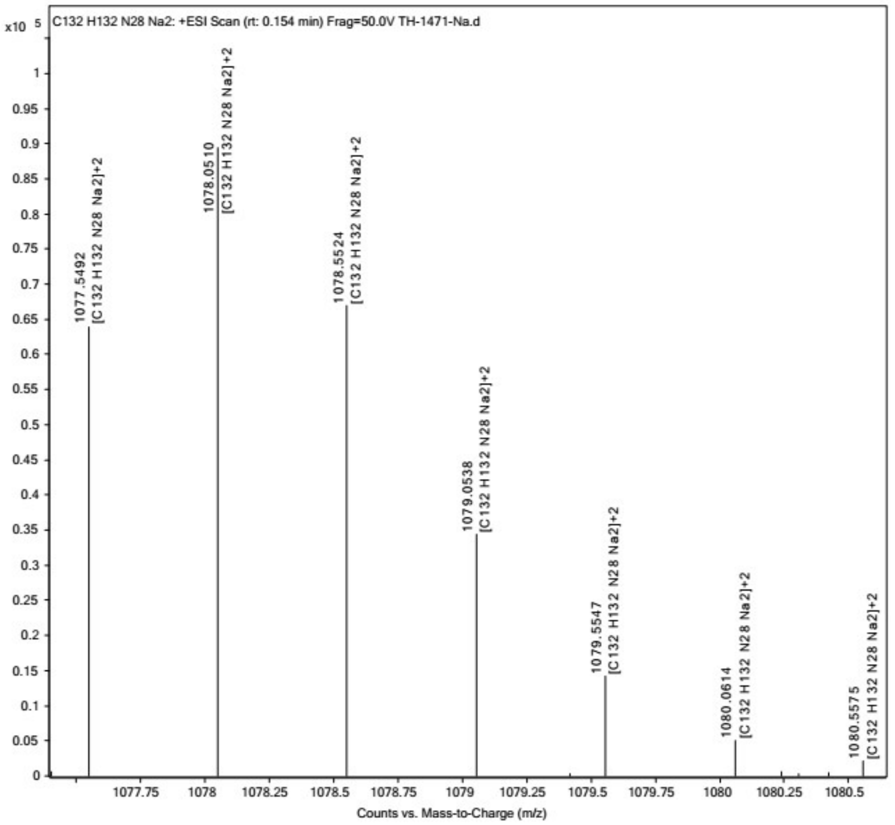

Fig. S30. ESI-HRMS of $(Na^{+})_2$⊂**T1**•2BArF⁻. ESI-HRMS *m*/*z* calcd for [**T1**+2Na]$^{+2}$ $C_{132}H_{132}N_{28}Na_2^{+2}$, 1077.5487; found 1077.5492.

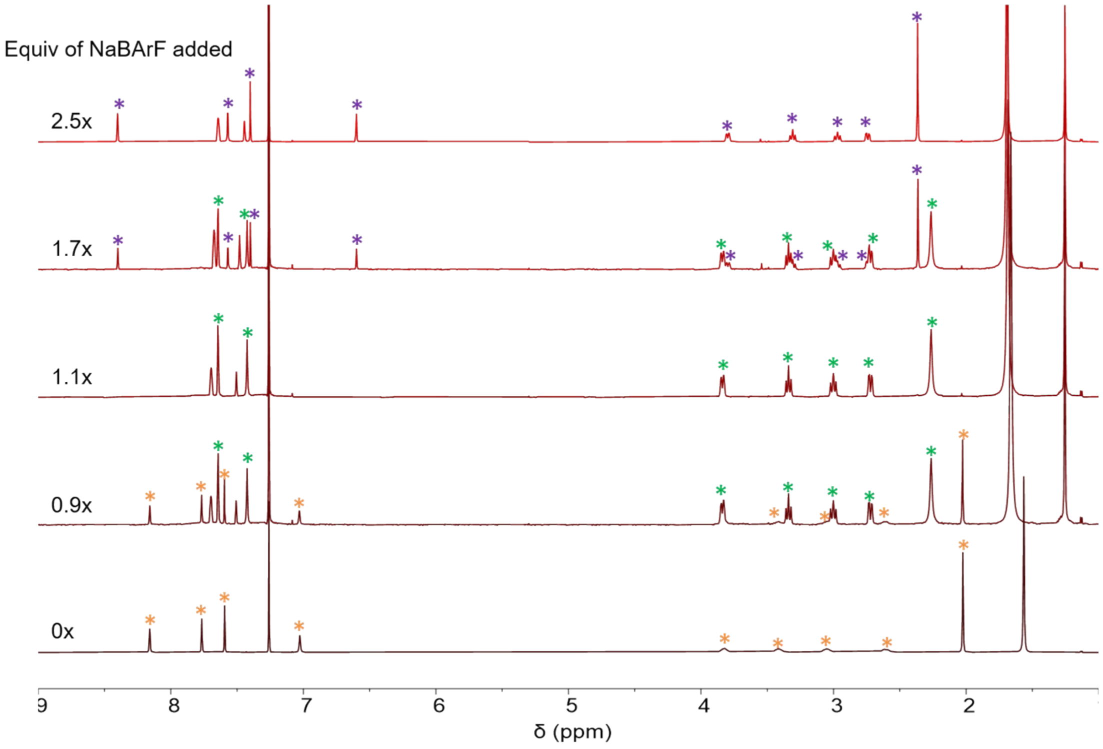

Fig. S31. ^{1}H NMR spectra of **T1** (600 MHz, $CDCl_3$, 298 K) after additing different amount of Na^{+}•BArF⁻. The resonances that are labelled with orange, green and violet stars correspond the **T1**, Na^{+}⊂**T1**•BArF⁻ and $(Na^{+})_2$⊂**T1**•2BArF⁻ respectively. The concentration of the **T1** is 6.54×10^{-4} M for all the NMR samples.

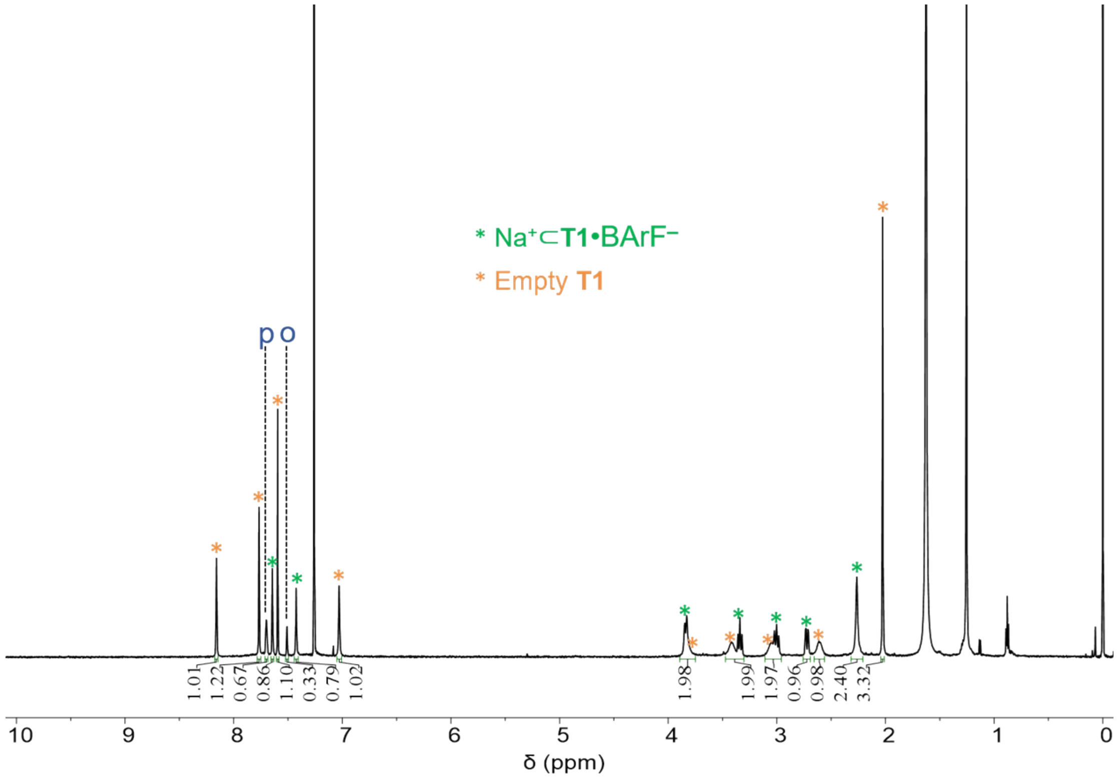

Fig. S32. ^{1}H NMR spectrum of **T1** (6.54×10^{-4} M, $CDCl_3$, 298 K) after adding 0.55 equiv. of Na^{+}•BArF⁻. The resonances labelled with orange and green stars correspond to **T1** and $Na^{+}\subset$**T1**•BArF⁻ respetively. The binding constant K_1 was calculated to be $(1.09\pm0.02)\times10^{4}$ M^{-1}, by integrating and comparing the resonances corresponding to **T1** and $Na^{+}\subset$**T1**•BArF⁻. The concentration of "free" Na^{+} was calculated by substracting the concentration of the complex from that of Na^{+}•BArF⁻ added into the system.

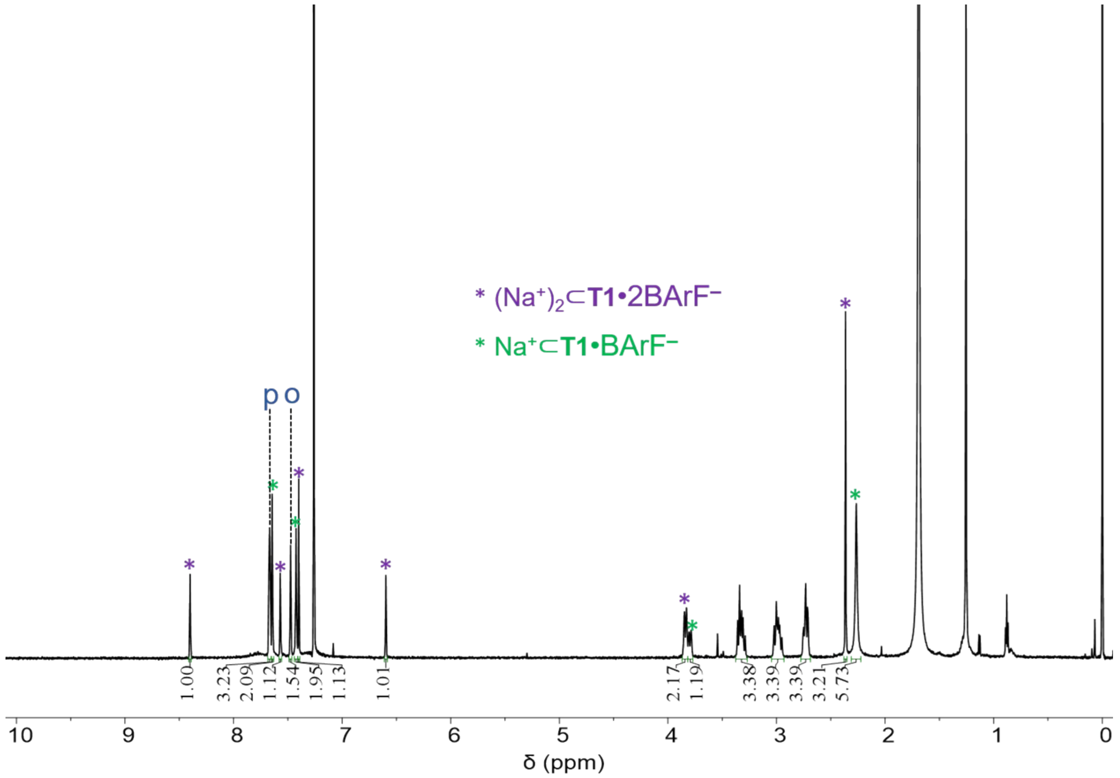

Fig. S33. ^{1}H NMR spectrum of **T1** (6.54×10^{-4} M, $CDCl_3$, 298 K) after adding 1.74 equiv. of Na^+•BArF⁻. The resonances labelled with green and purple stars correspond to Na^+⊂**T1**•BArF⁻ and $(Na^+)_2$⊂**T1**•2BArF⁻, respetively. The binding constant K_2 was calculated to be $(1.73\pm0.47)\times10^3$ M^{-1}, by integrating and comparing the resonances corresponding to Na^+⊂**T1**•BArF⁻ and $(Na^+)_2$⊂**T1**•2BArF⁻. The concentration of "free" Na^+ was calculated by substracting the concentration of the Na^+ trapped in the two complexes from that of Na^+•BArF⁻ added into the system.

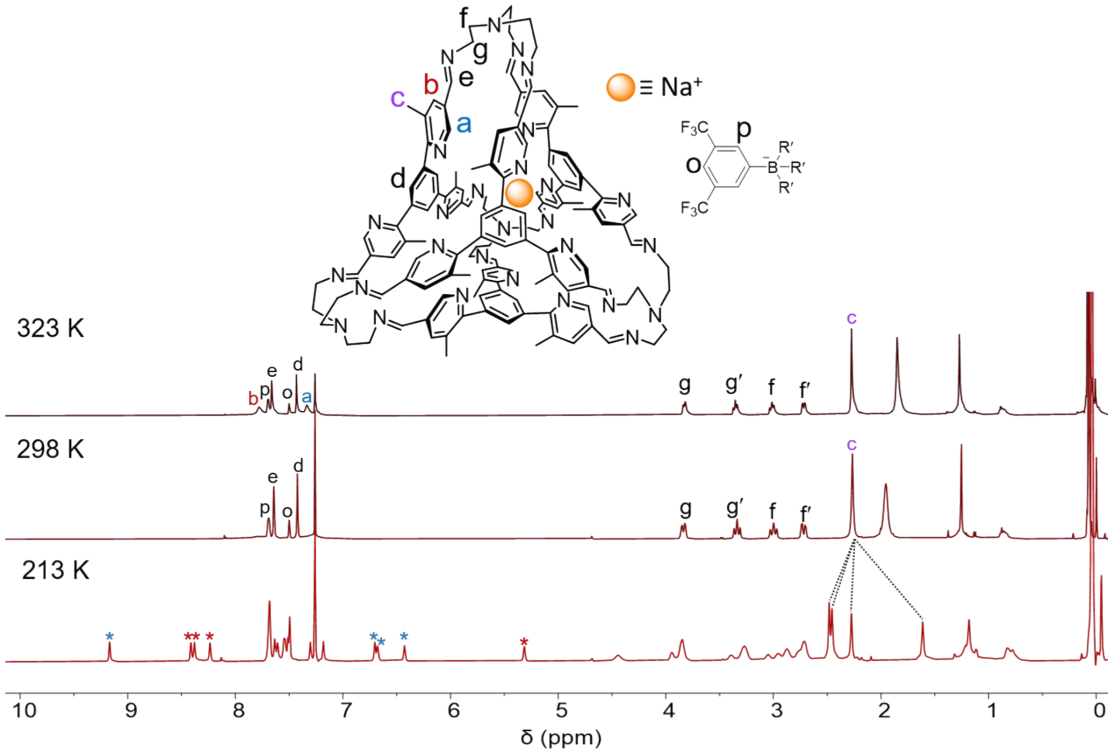

Fig. S34. ^{1}H NMR spectra of Na^+⊂**T1**•BArF$^-$ recorded at 213 K (bottom), 298 K (middle) and 323 K (top).

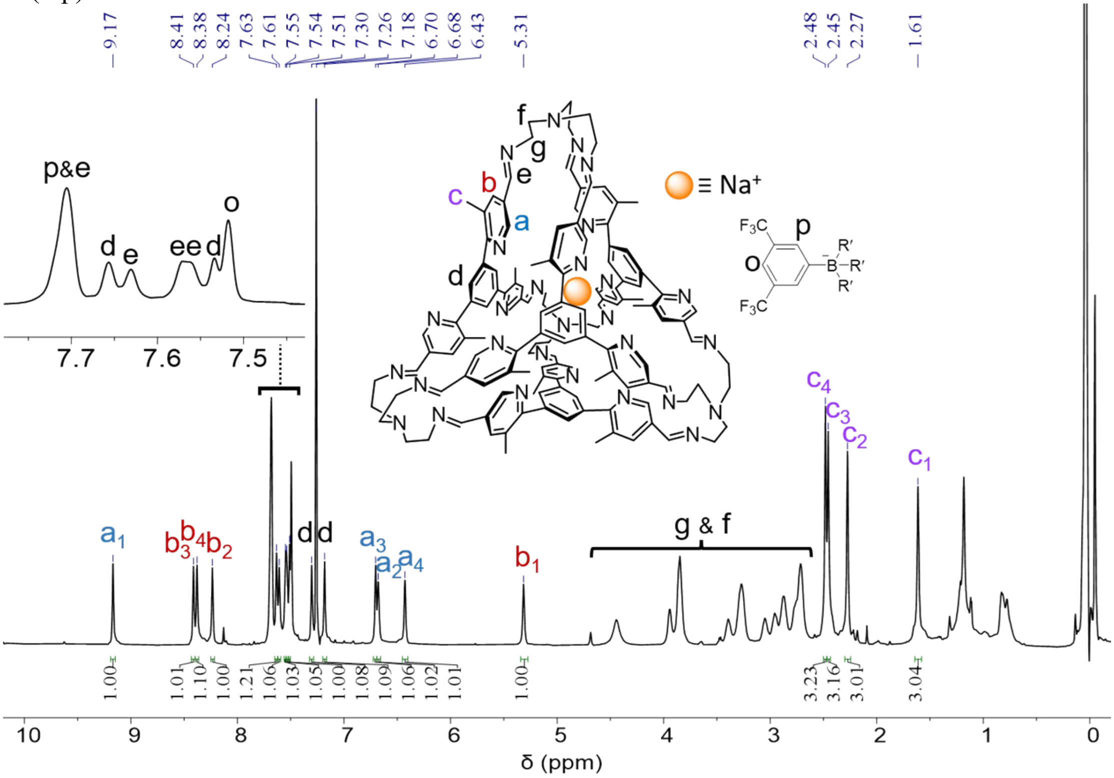

Fig. S35. A full ^{1}H NMR spectrum of Na^+⊂**T1**•BArF$^-$ (600 MHz, $CDCl_3$, 213 K).

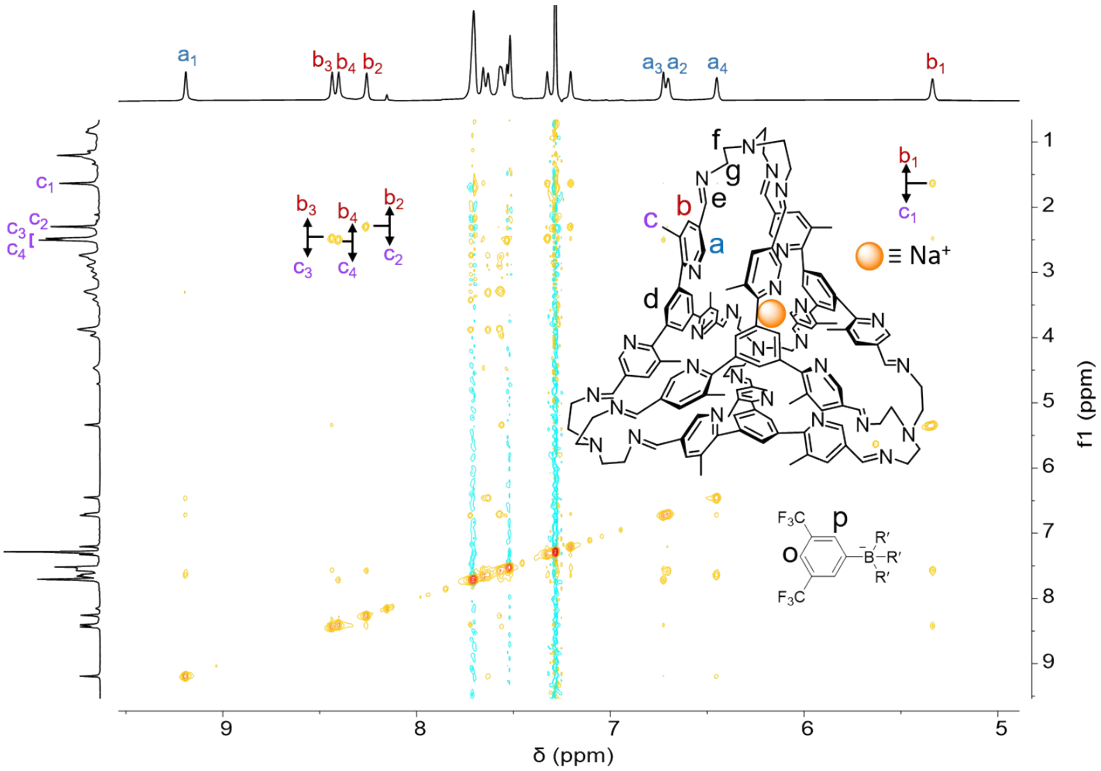

Fig. S36. ^{1}H-^{1}H NOESY spectrum of Na^+⊂**T1**•BArF$^-$ (600 MHz, $CDCl_3$, 213 K). Key correlation peaks are labeled in the spectrum.

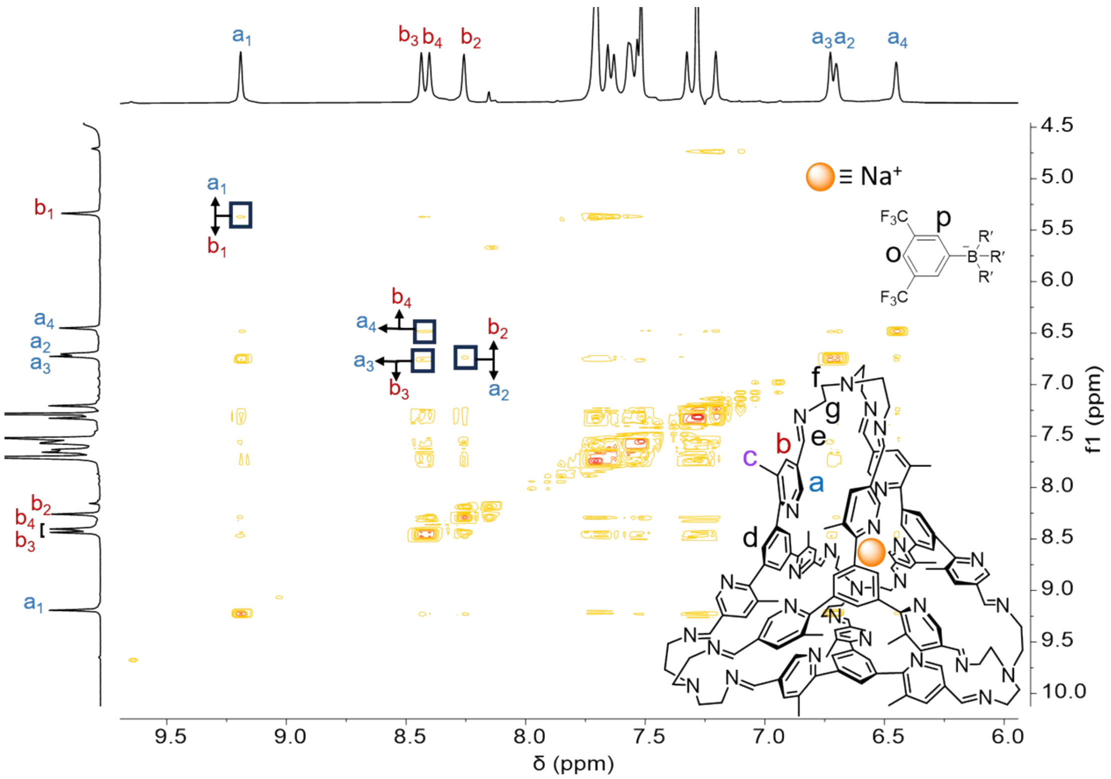

Fig. S37. ^{1}H-^{1}H COSY spectrum of Na^{+}⊂**T1**•BArF^{-} (600 MHz, CDCl$_3$, 213 K). Key correlation peaks are labeled in the spectrum.

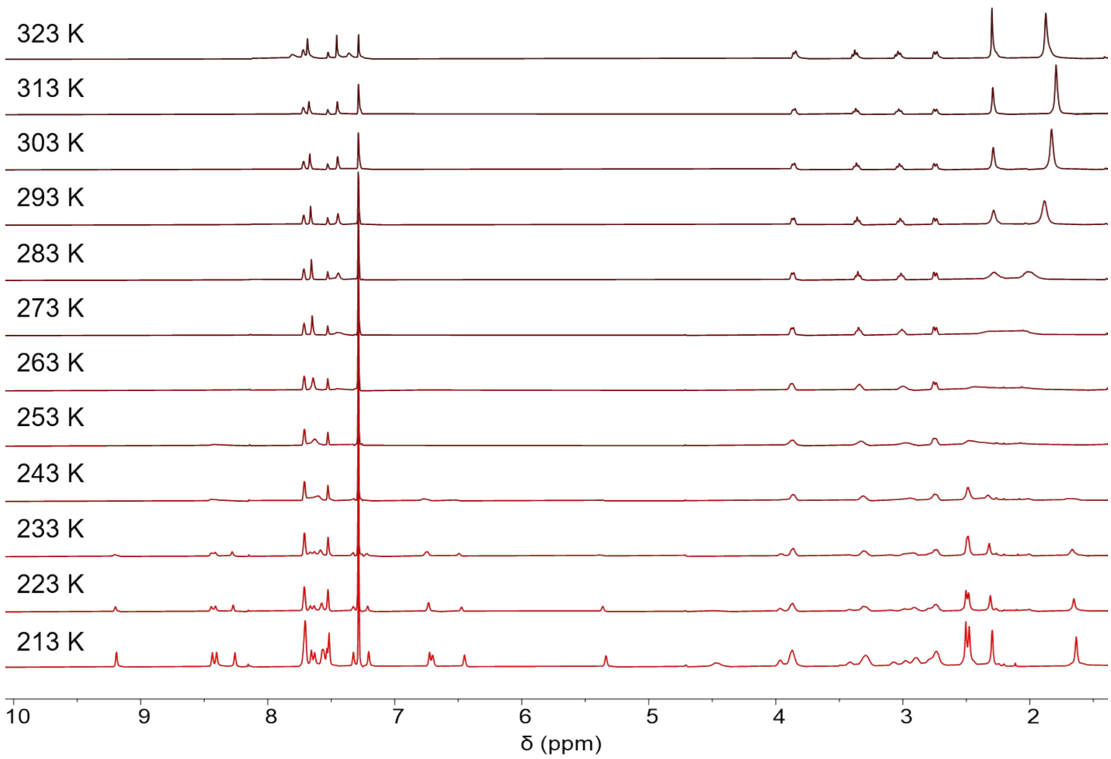

Fig. S38. ^{1}H NMR (600MHz, CDCl$_3$) of Na^{+}⊂**T1**•BArF^{-} recorded at variable temperatures, including 213 K (bottom) and 323 K (top).

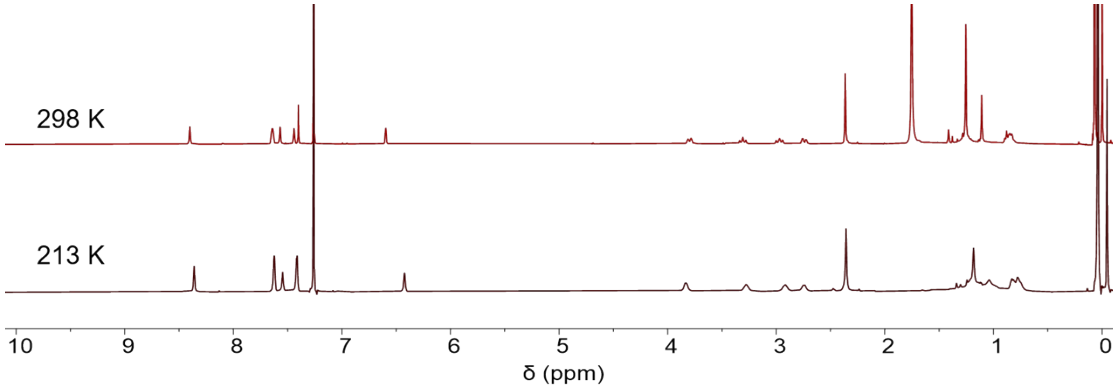

Fig. S39. ^{1}H NMR spectrum (600 MHz, $CDCl_3$) of $(Na^+)_2$⊂**T1**•2BArF$^-$ recorded at 213 K (bottom) and 298 K (top). The two spectra are essentially the same.

(3) Cs^+ in cage **T1**

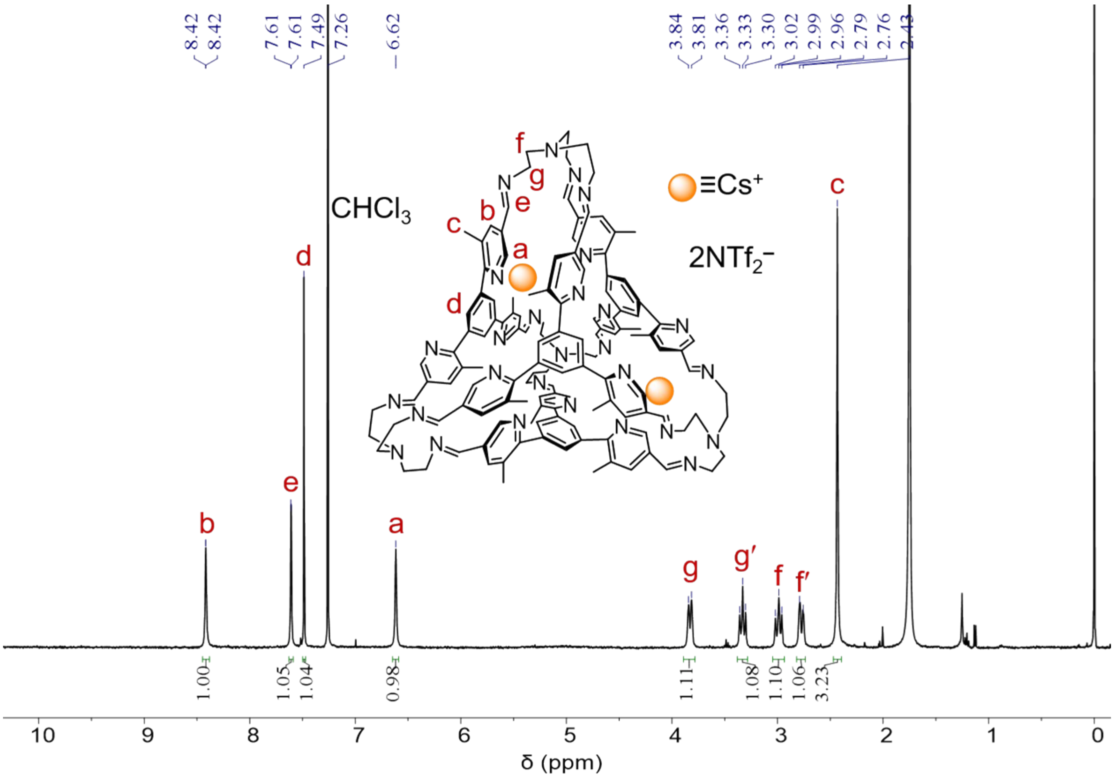

Fig. S40. ^{1}H NMR spectrum of $(Cs^+)_2 \subset$**T1**$\bullet 2NTf_2^-$ (400 MHz, $CDCl_3$, 298 K).

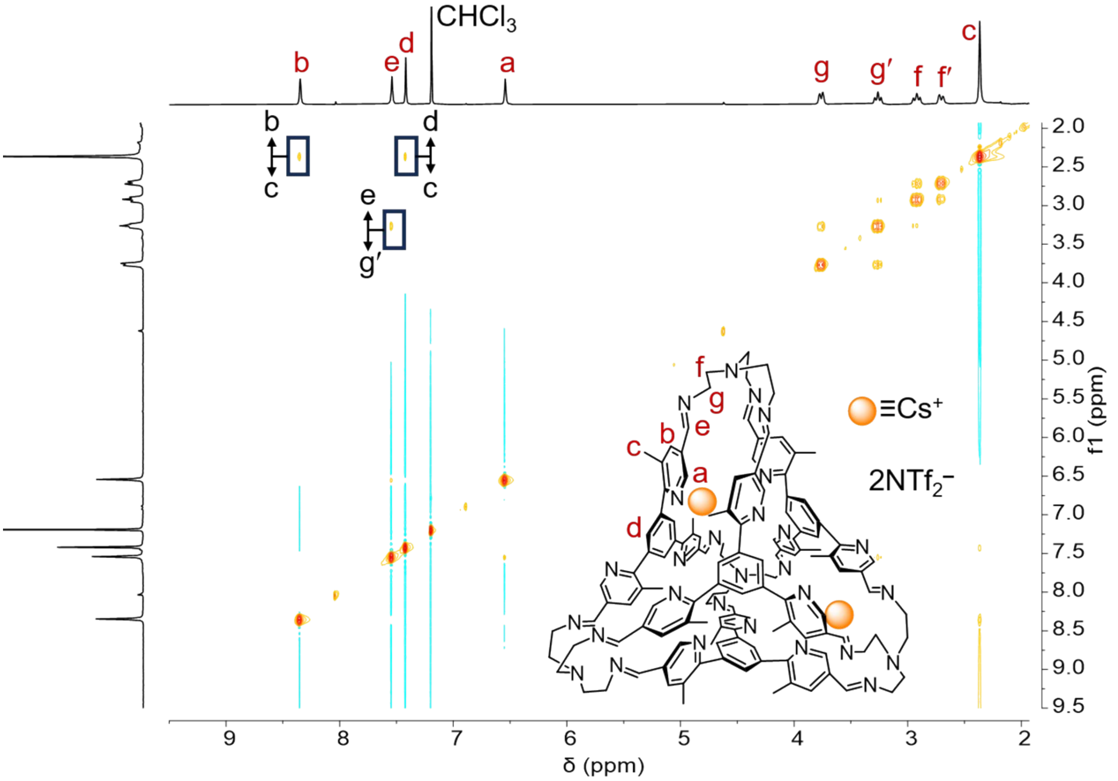

Fig. S41. ^{1}H-^{1}H NOESY spectrum of $(Cs^+)_2 \subset$**T1**$\bullet 2NTf_2^-$ (400 MHz, $CDCl_3$, 298 K). Key correlation peaks are labeled in the spectrum.

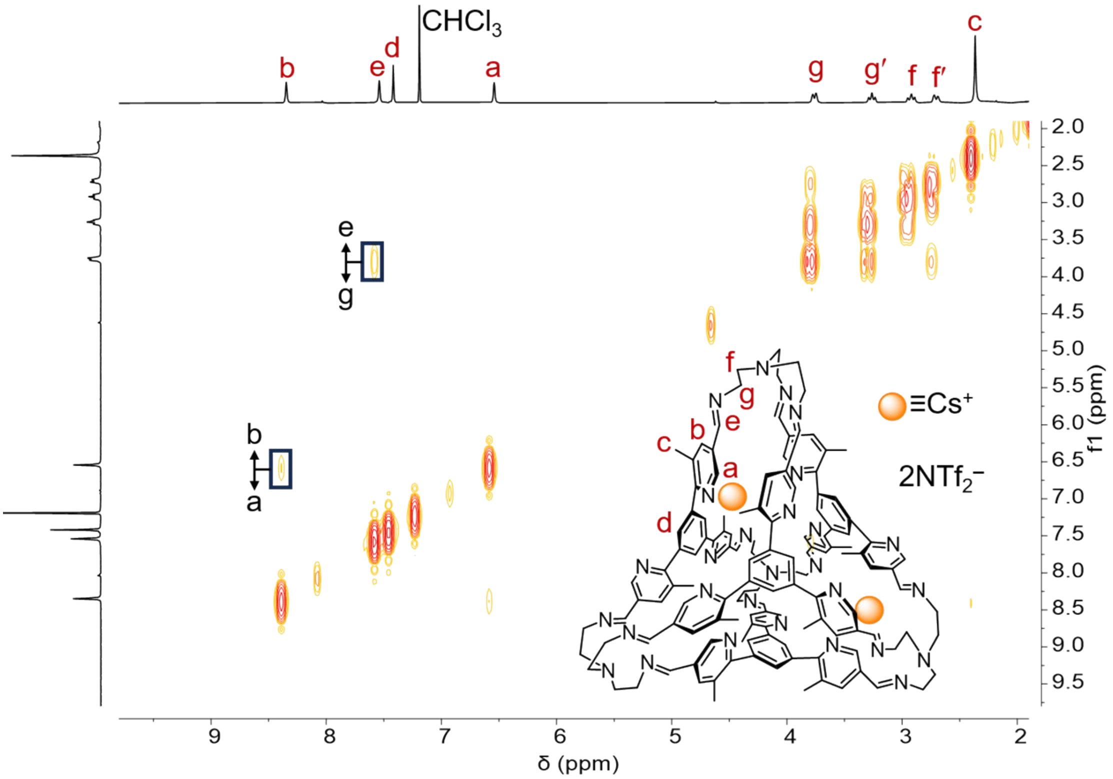

Fig. S42. ^1H-^1H COSY spectrum of $(Cs^+)_2{\subset}$**T1**•$2NTf_2^-$ (400 MHz, $CDCl_3$, 298 K). Key correlation peaks are labeled in the spectrum.

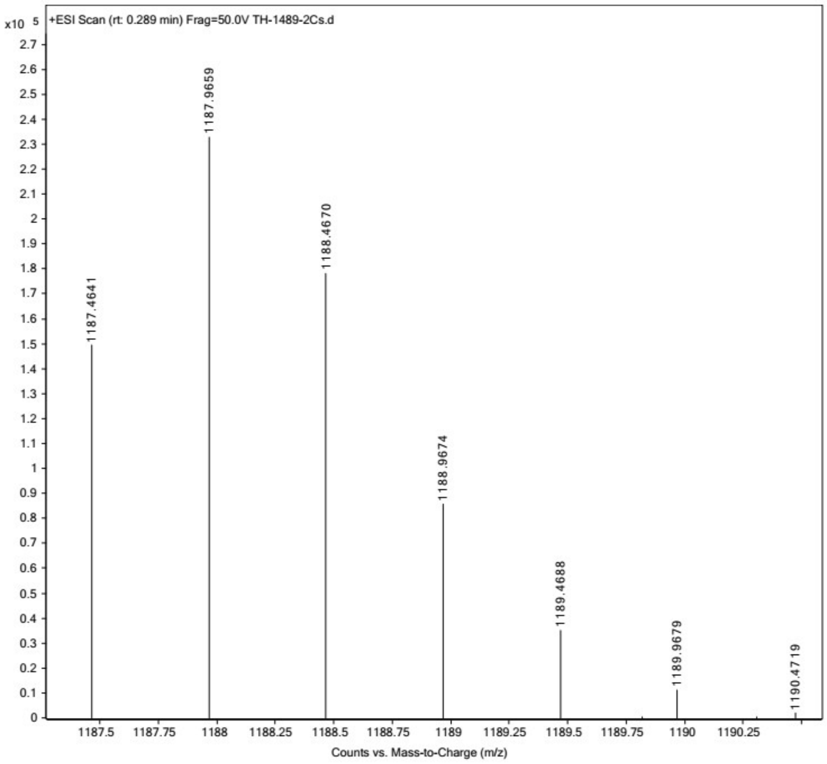

Fig. S43. ESI-HRMS of $(Cs^+)_2{\subset}$**T1**•$2NTf_2^-$. ESI-HRMS *m*/*z* calcd for [**T1**+2Cs]$^{+2}$ $C_{132}H_{132}N_{28}Cs_2^{+2}$, 1187.4644; found 1187.4641.

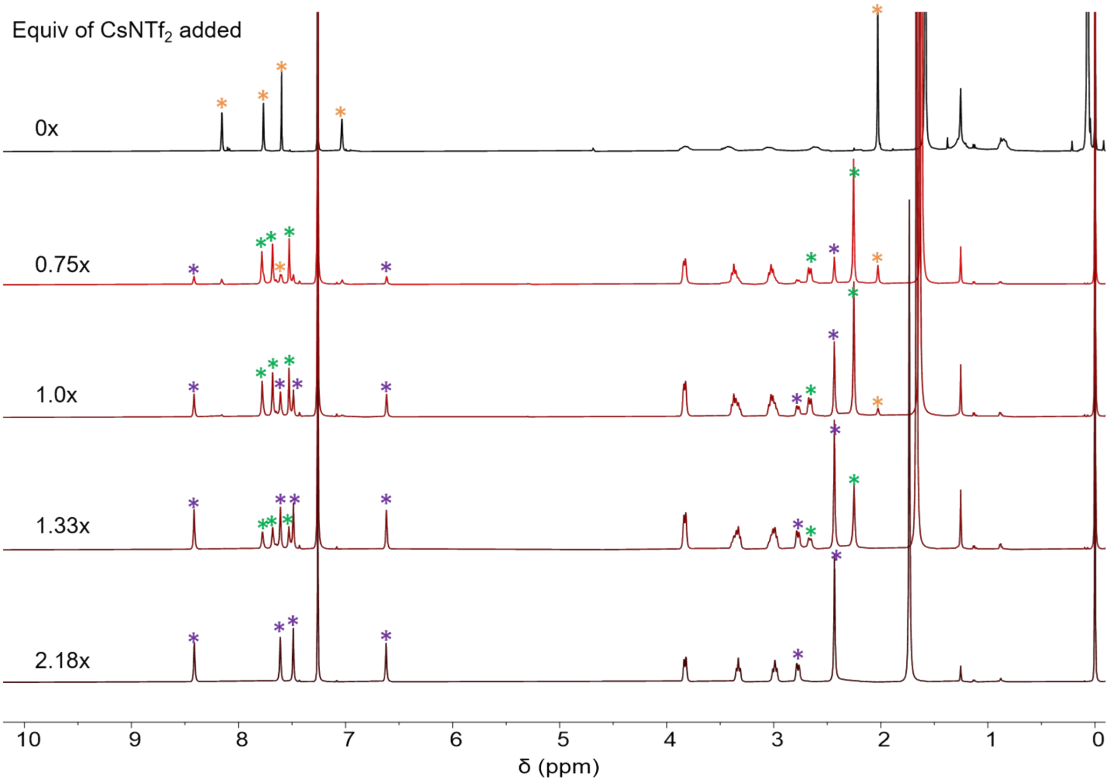

Fig. S44. ^{1}H NMR spectra (600 MHz, $CDCl_3$, 298 K) of **T1** after addition of different amout of Cs^+•NTf_2^-. The resonances that are labelled with orange, green and violet stars correspond the **T1**, Cs^+⊂**T1**•NTf_2^- and $(Cs^+)_2$⊂**T1**•$2NTf_2^-$ respectively. The concentration of the **T1** is 6.4×10^{-4} M for all the NMR samples.

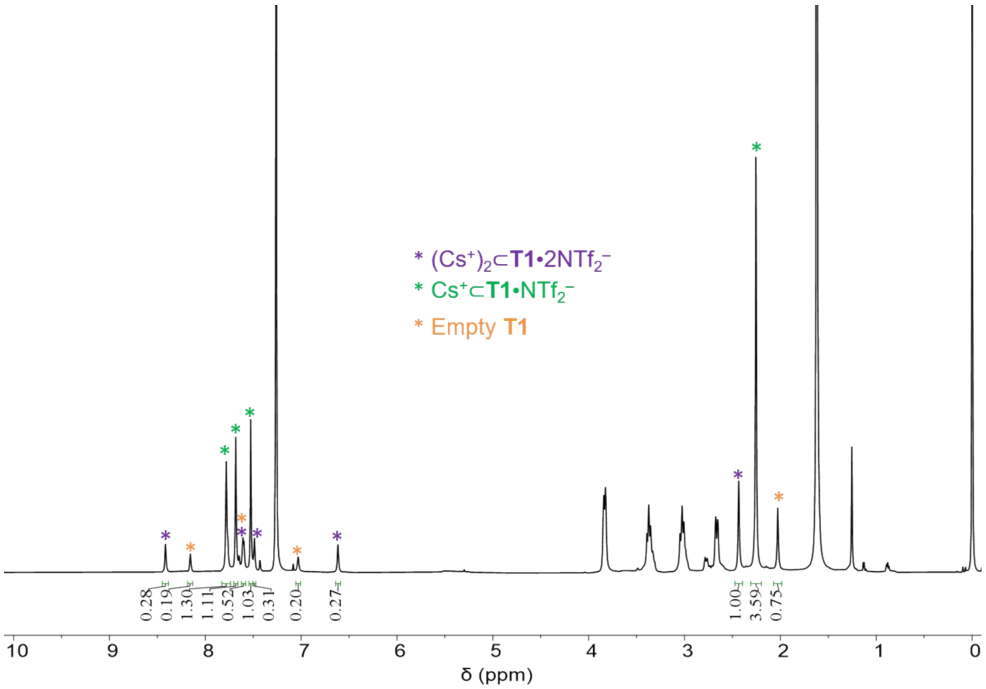

Fig. S45. ^{1}H NMR spectrum of **T1** (6.4×10^{-4} M, $CDCl_3$, 298 K) after adding 0.75 equiv. of Cs^+•NTf_2^-. The resonances labelled with orange, green and violet stars correspond to **T1**, Cs^+⊂**T1**•NTf_2^- and $(Cs^+)_2$⊂**T1**•$2NTf_2^-$ respetively. The binding constant K_1 was calculated to be $(2.94\pm0.29)\times10^3$ M^{-1}, by integrating and comparing the resonances corresponding to **T1**, Cs^+⊂**T1**•NTf_2^- and $(Cs^+)_2$⊂**T1**•$2NTf_2^-$. The concentration of "free" Cs^+ was calculated by substracting the concentration of the complex from that of Cs^+•NTf_2^- added into the system.

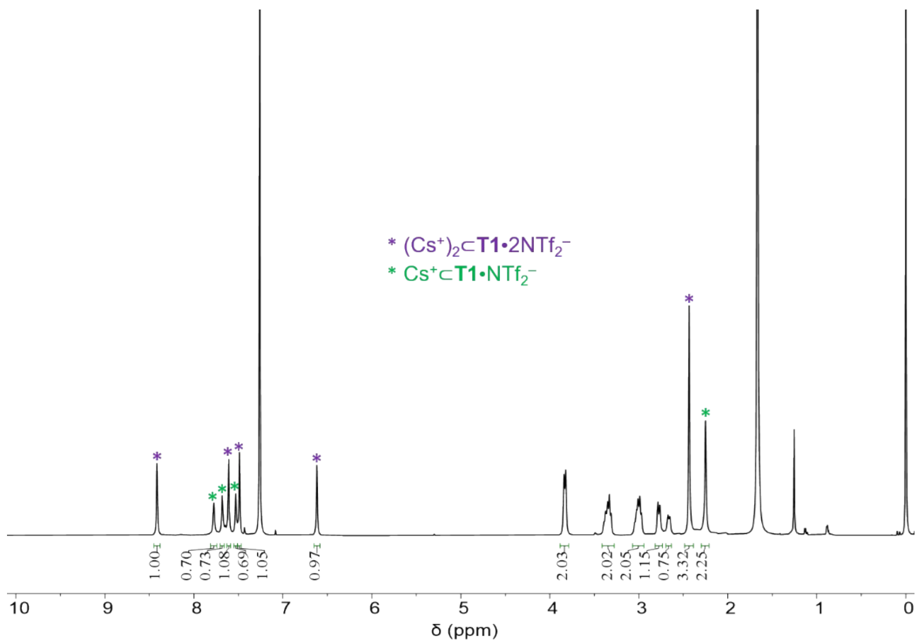

Fig. S46. ^{1}H NMR spectrum of **T1** (6.4×10^{-4} M, $CDCl_3$, 298 K) after adding 1.33 equiv. of Cs^+•NTf_2^-. The resonances labelled with green and purple stars correspond to $Cs^+\subset$**T1**•NTf_2^- and $(Cs^+)_2\subset$**T1**•2NTf_2^-, respetively. The binding constant K_2 was calculated to be $(6.4\pm0.1)\times10^2$ M^{-1}, by integrating and comparing the resonances corresponding to $Cs^+\subset$**T1**•NTf_2^- and $(Cs^+)_2\subset$**T1**•2NTf_2^-. The concentration of "free" Cs^+ was calculated by substracting the concentration of the Cs^+ trapped in the two complexes from that of Cs^+•NTf_2^- added into the system.

(4) K^+ in cage **T1**

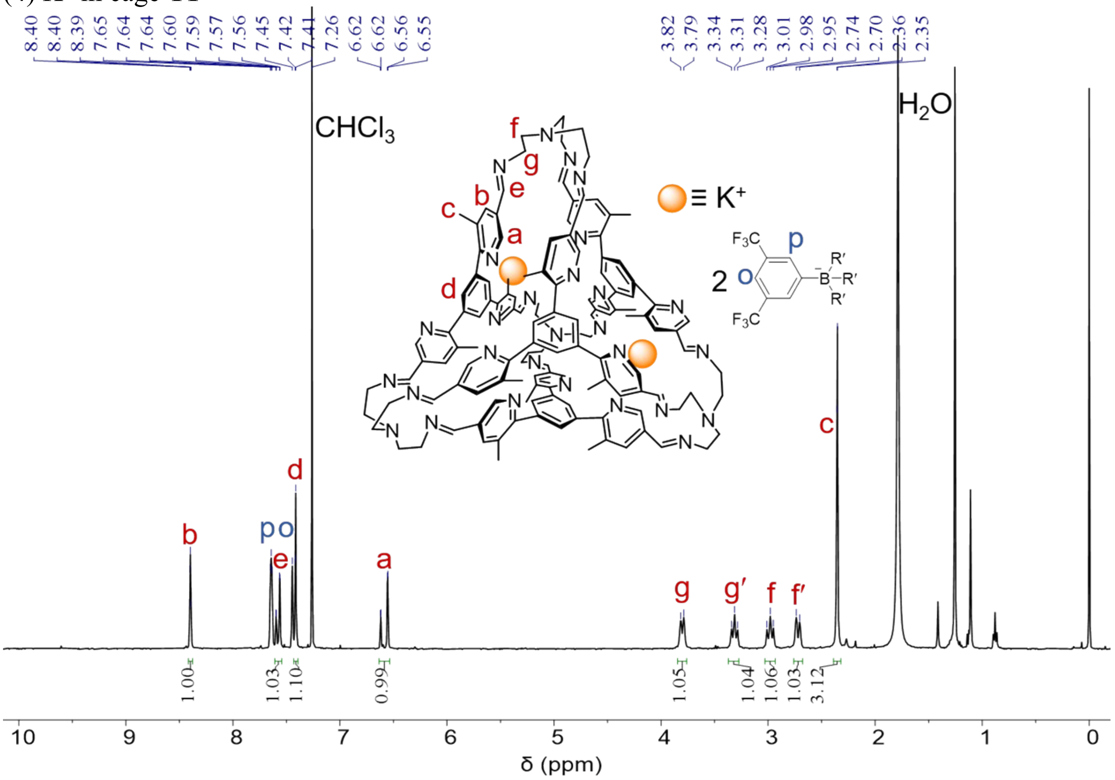

Fig. S47. ^{1}H NMR spectrum of $(K^+)_2$⊂**T1**•2BArF$^-$ (400 MHz, $CDCl_3$, 298 K). The resonances labelled with blue letters *p* and *o* correspond to the BArF$^-$ counterion of K^+.

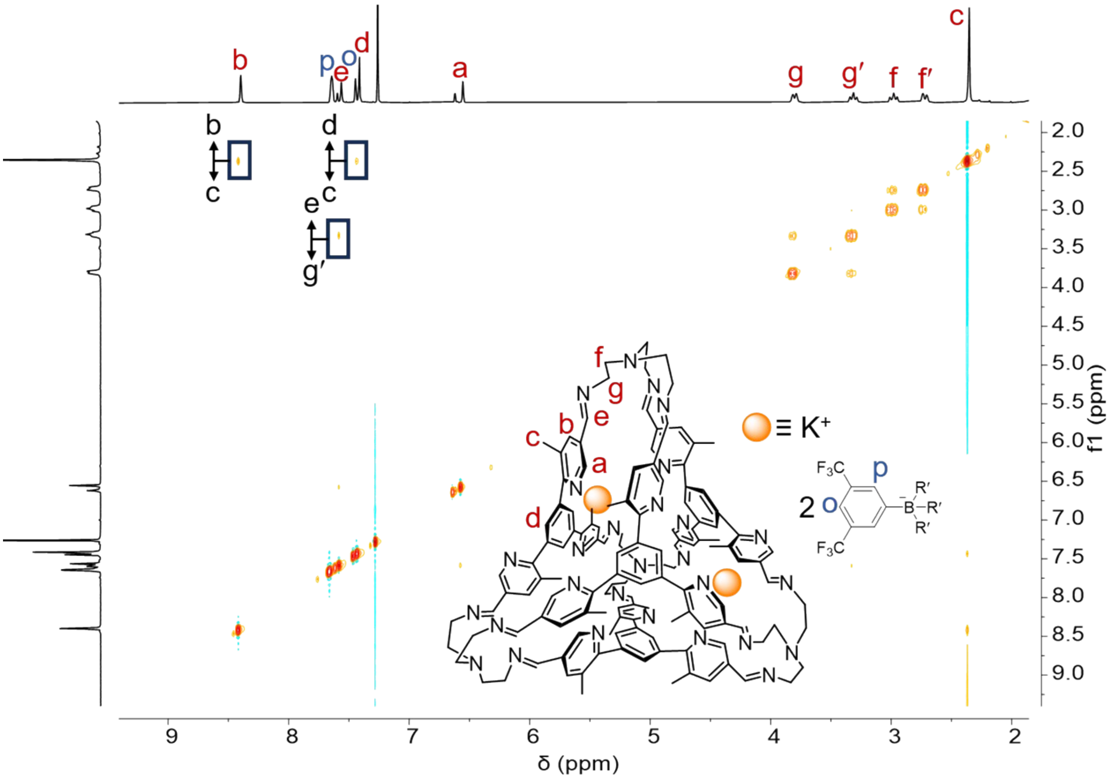

Fig. S48. ^{1}H-^{1}H NOESY spectrum of $(K^+)_2$⊂**T1**•2BArF$^-$ (400 MHz, $CDCl_3$, 298 K). Key correlation peaks are labeled in the spectrum.

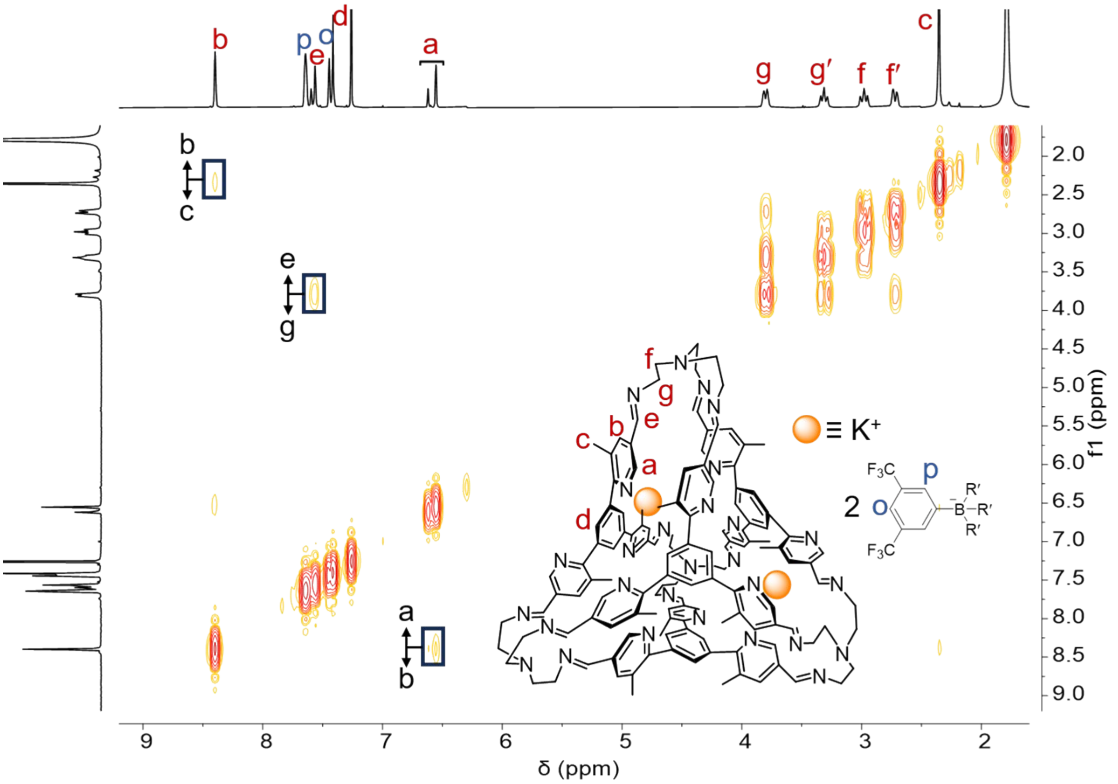

Fig. S49. ^1H-^1H COSY spectrum of $(K^+)_2$⊂**T1**•2BArF⁻ (400 MHz, $CDCl_3$, 298 K). Key correlation peaks are labeled in the spectrum.

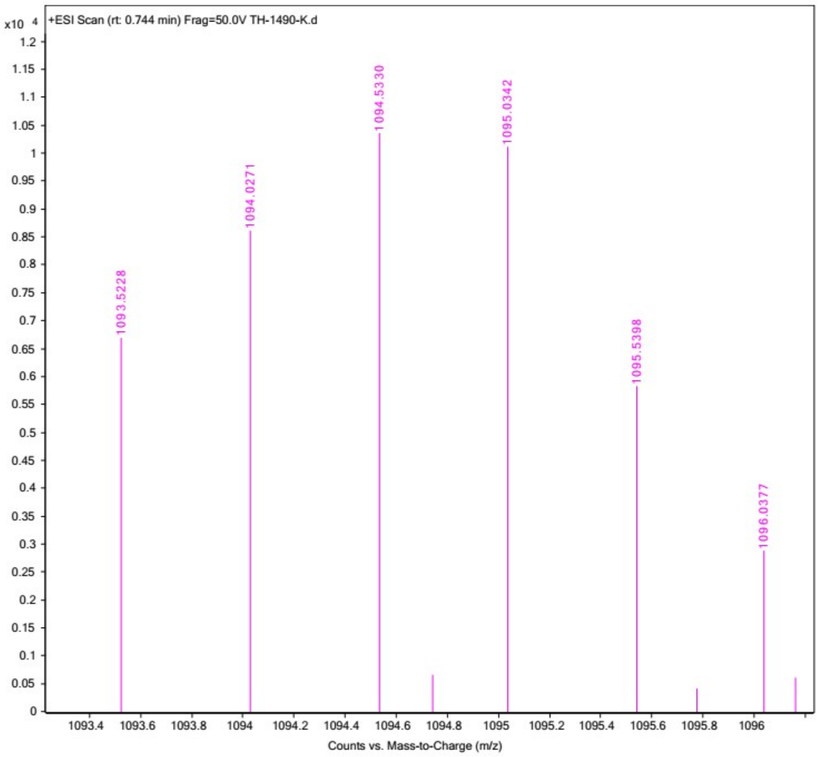

Fig. S50. ESI-HRMS of $(K^+)_2$⊂**T1**•2BArF⁻. ESI-HRMS *m*/*z* calcd for [**T1**+2K]$^{+2}$ $C_{132}H_{132}N_{28}K_2^{+2}$, 1093.5226; found 1093.5228.

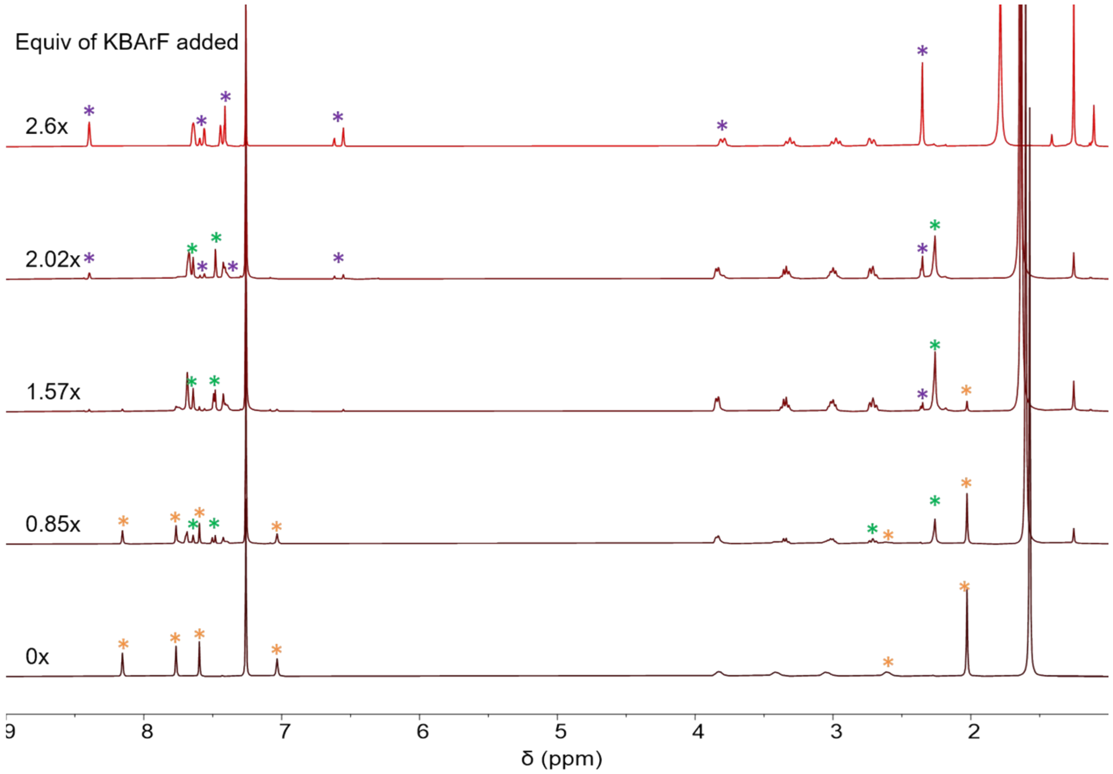

Fig. S51. ^{1}H NMR spectra (600 MHz, $CDCl_3$, 298 K) of **T1** after addition of different amout of K^+•BArF$^-$. The resonances that are labelled with orange, green and violet stars correspond the **T1**, K^+⊂**T1**•BArF$^-$ and $(K^+)_2$⊂**T1**•2BArF$^-$ respectively. The concentration of the **T1** is 4.67×10^{-4} M for all the NMR samples.

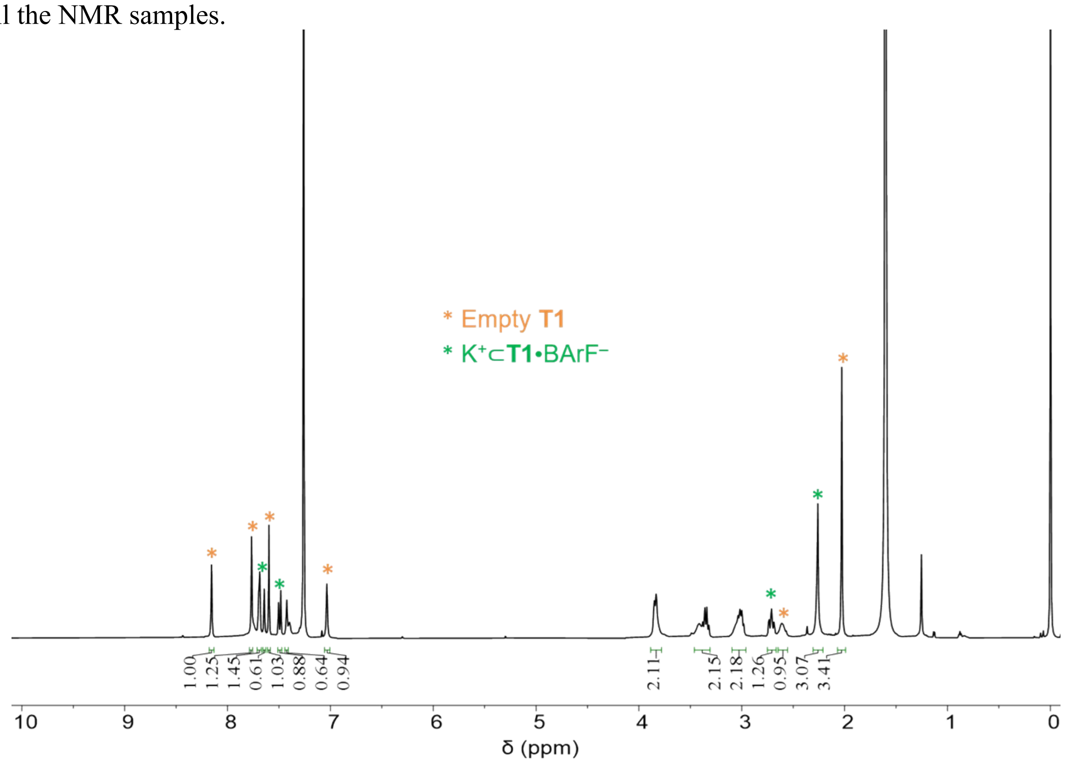

Fig. S52. ^{1}H NMR spectrum of **T1** (4.67×10^{-4} M, $CDCl_3$, 298 K) after adding 0.85 equiv. of K^+•BArF$^-$. The resonances labelled with orange and green stars correspond to **T1** and K^+⊂**T1**•BArF$^-$ respetively. The binding constant K_1 was calculated to be $(2.96\pm0.76)\times10^{3}$ M^{-1}, by integrating and comparing the resonances corresponding to **T1** and K^+⊂**T1**•BArF$^-$. The concentration of "free" K^+ was calculated by substracting the concentration of the complex from that of K^+•BArF$^-$ added into the system.

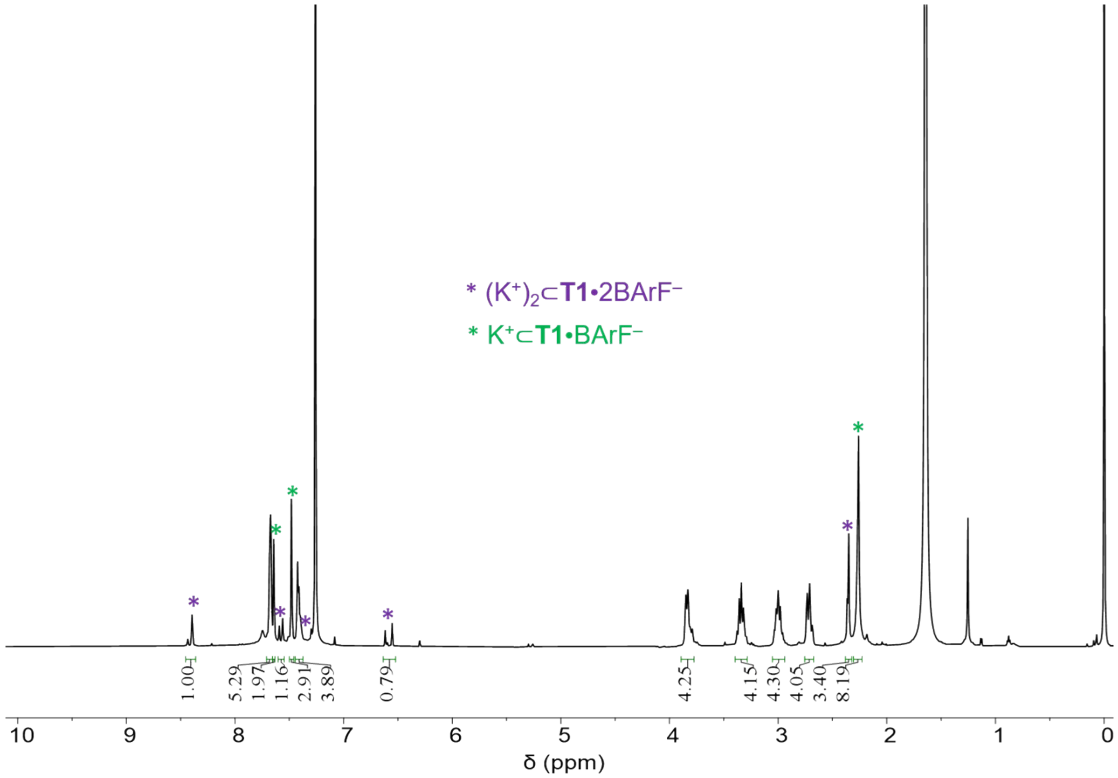

Fig. S53. ^{1}H NMR spectrum of **T1** (4.67×10^{-4} M, $CDCl_3$, 298 K) after adding 2.02 equiv. of KBArF. The resonances labelled with green and purple stars correspond to $K^+\subset$**T1**•$BArF^-$. and $(K^+)_2\subset$**T1**•$2BArF^-$. respetively. The binding constant K_2 was calculated to be $(7.3\pm0.3)\times10^2$ M^{-1}, by integrating and comparing the resonances corresponding to $K^+\subset$**T1**•$BArF^-$ and $(K^+)_2\subset$**T1**•$2BArF^-$. The concentration of "free" K^+ was calculated by substracting the concentration of the K^+ trapped in the two complexes from that of K^+•$BArF^-$ added into the system.

(5) Li^{+} in cage **T1**

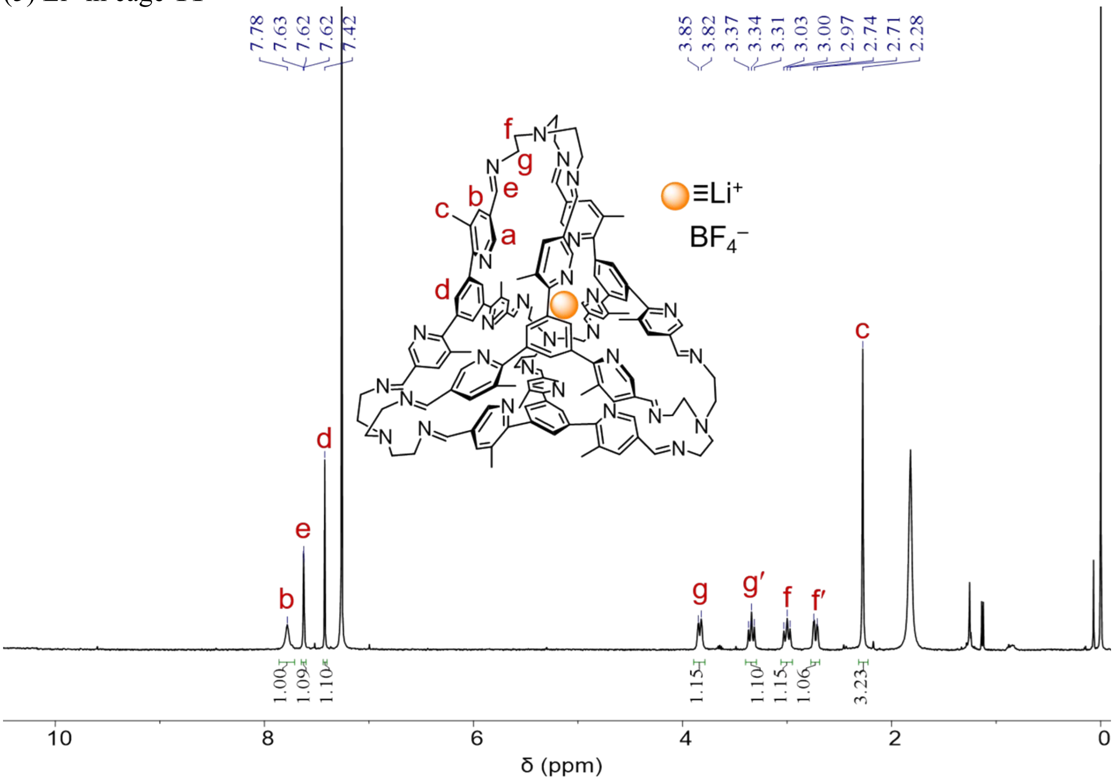

Fig. S54. ^{1}H NMR spectrum of Li^{+}⊂**T1**•BF_4^{-} (400 MHz, $CDCl_3$, 298 K).

Due to the poor solubility of Li^{+}•BF_4^{-} in chloroform, the binding constant could not be calculated in pure deuterated chloroform by ^{1}H NMR spectroscopy. The poor solubility of Li^{+}•BF_4^{-} only allows the cage to recognize one Li^{+} in chloroform. In addition, after accommodation of Li^{+}, **T1** underwent decomposition into **F1** and TREN after a few hours, yielding a yellow precipitate. A mixture of solvent ($CDCl_3$: CD_3CN=1:1) was thus used, in which Li^{+}•BF_4^{-} was soluble.

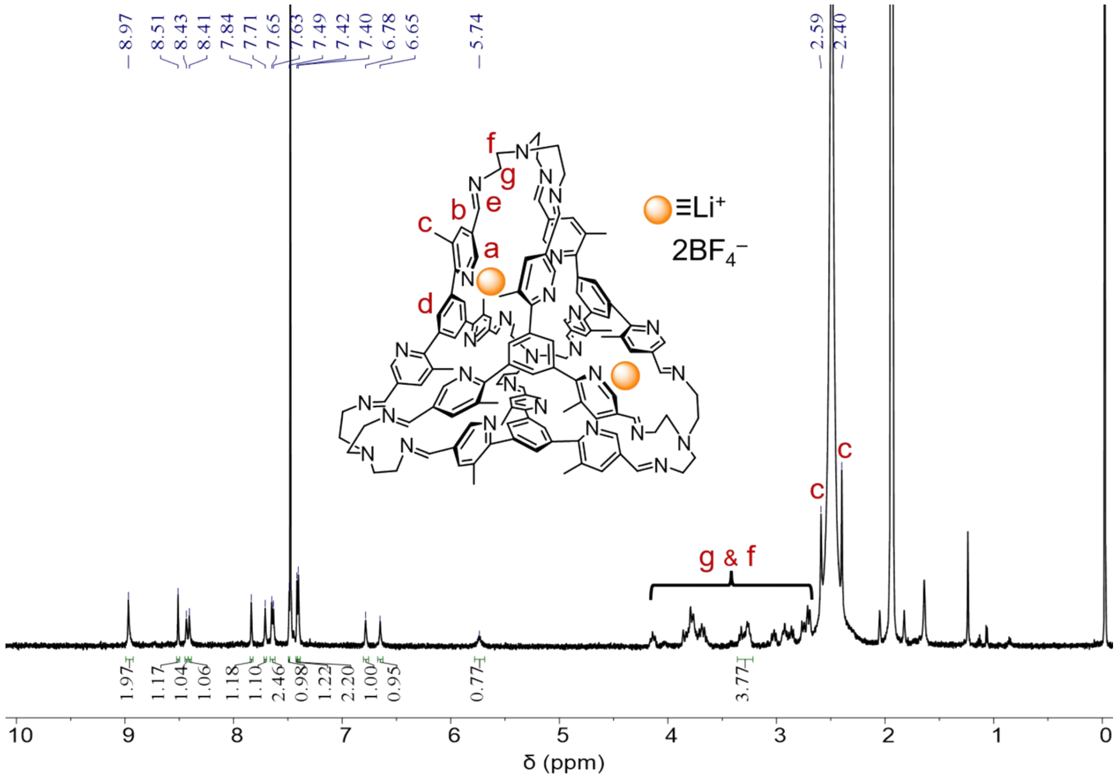

Fig. S55. ^{1}H NMR spectrum of $(Li^+)_2$⊂**T1**•$2BF_4^-$ (400 MHz, $CDCl_3$: CD_3CN=1:1, 298 K).

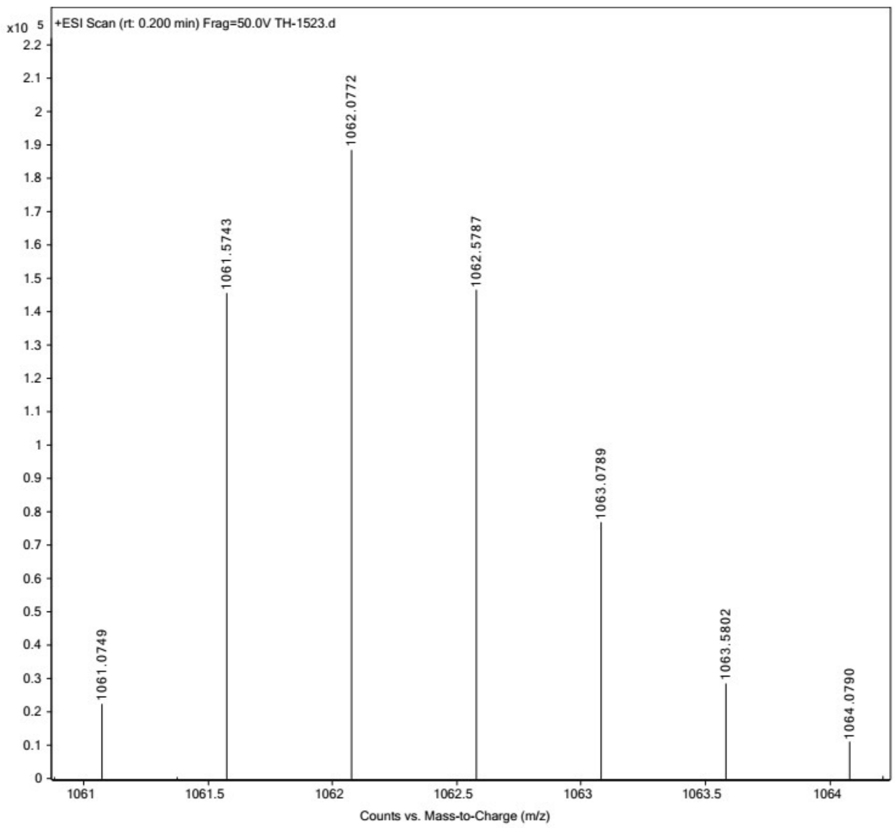

Fig. S56. ESI-HRMS of $(Li^+)_2$⊂**T1**•$2BF_4^-$. ESI-HRMS *m*/*z* calcd for [**T1**+2Li]$^{+2}$ $C_{132}H_{132}N_{28}Li_2^{+2}$, 1061.5749; found 1061.5743.

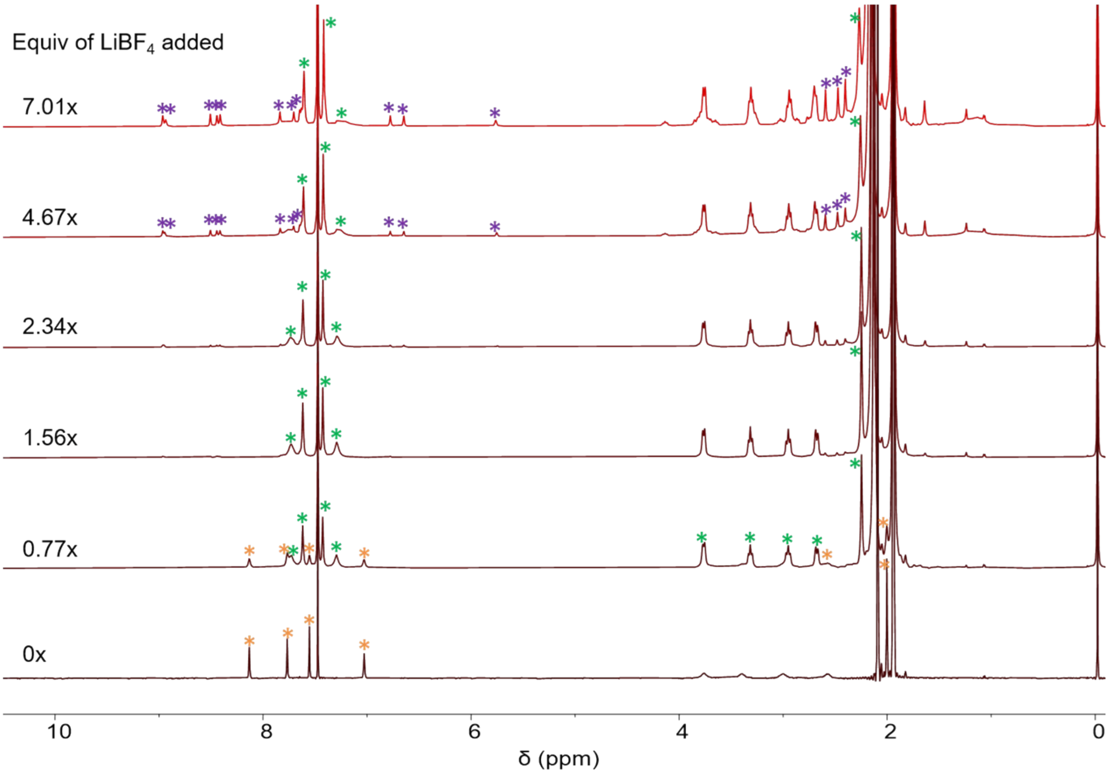

Fig. S57. ^{1}H NMR spectra(600 MHz, $CDCl_3$:CD_3CN=1:1, 298 K) of **T1** after addition of different amout of Li^+•$BF_4^–$. The resonances that are labelled with orange, green and violet stars correspond the **T1**, Li^+⊂**T1**•$BF_4^–$ and $(Li^+)_2$⊂**T1**•2$BF_4^–$ respectively. The concentration of the **T1** is 4.07×10^{-4} M for all the NMR samples.

To 0.4 mL 4.07×10^{-4} M **T1** in $CDCl_3$:CD_3CN=1:1 solution gradually added the Li^+•$BF_4^–$ (0.27 mg, dissolved in 0.6 mL 4.07×10^{-4} M **T1** in $CDCl_3$:CD_3CN=1:1 solution). The host concentration remained unchanged during titration. The binding constant K_1 is $(1.93\pm0.16)\times10^5$ M^{-1} and K_2 is $(2.3\pm0.1)\times10^2$ M^{-1} to determine the concentration of each component by integration.

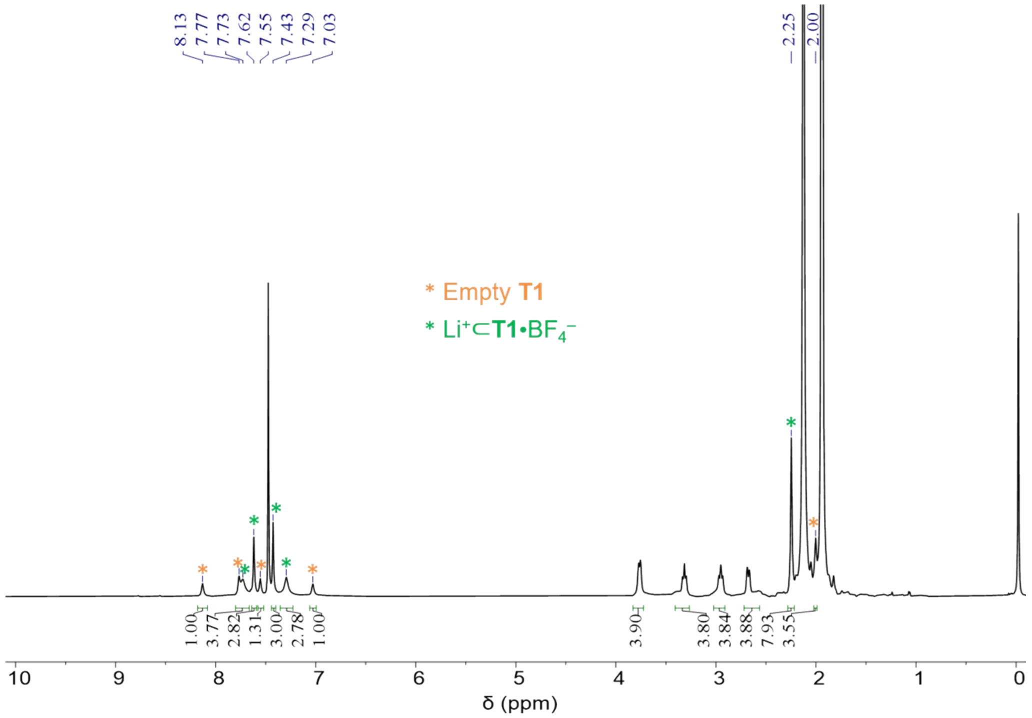

Fig. S58. ^{1}H NMR spectrum of **T1** (4.07×10^{-4} M, $CDCl_3$:CD_3CN=1:1, 298 K) after adding 0.77 equiv. of Li^+•BF_4^-. The resonances labelled with orange and green stars correspond to **T1** and Li^+⊂**T1**•BF_4^- respetively. The binding constant K_1 was calculated to be $(1.93\pm0.16)\times10^5$ M^{-1}, by integrating and comparing the resonances corresponding to **T1** and Li^+⊂**T1**•BF_4^-. The concentration of "free" Li^+ was calculated by substracting the concentration of the complex from that of $LiBF_4$ added into the system.

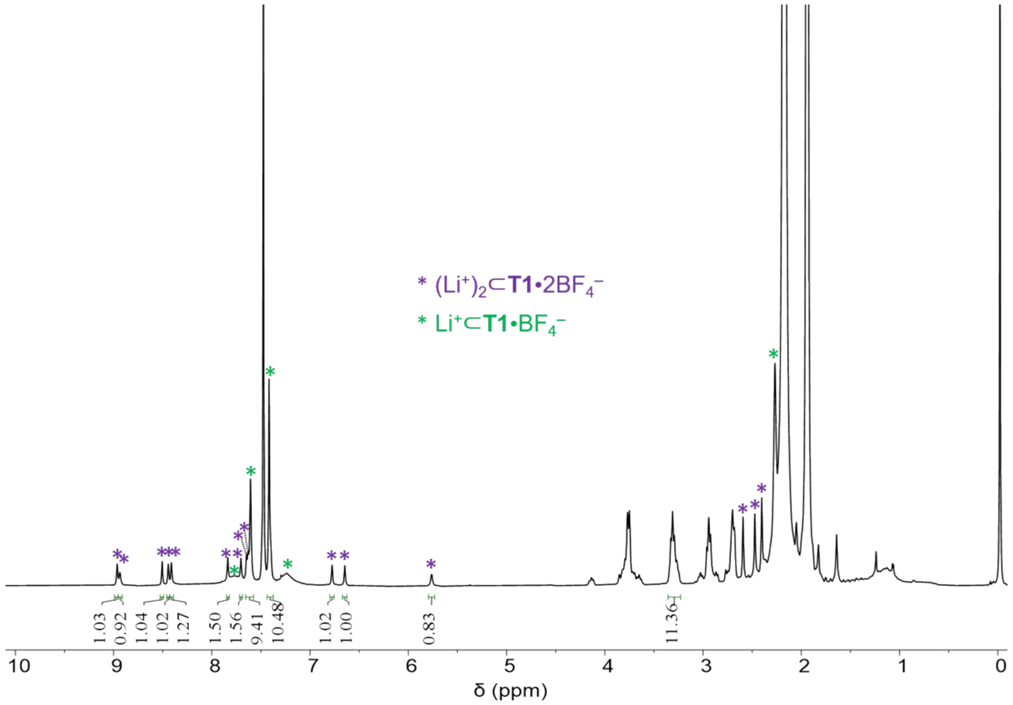

Fig. S59. ^{1}H NMR spectrum of **T1** (4.07×10^{-4} M, $CDCl_3$:CD_3CN=1:1, 298 K) after adding 7.01 equiv. of $LiBF_4$. The resonances labelled with green and purple stars correspond to Li^+⊂**T1**•BArF⁻. and $(Li^+)_2$⊂**T1**•2BArF⁻. respetively. The binding constant K_2 was calculated to be $(2.3\pm0.1)\times10^2$ M^{-1}, by integrating and comparing the resonances corresponding to Li^+⊂**T1**•BF_4^- and $(Li^+)_2$⊂**T1**•2BF_4^-. The concentration of "free" Li^+ was calculated by substracting the concentration of the Li^+ trapped in the two complexes from that of $LiBF_4$ added into the system.

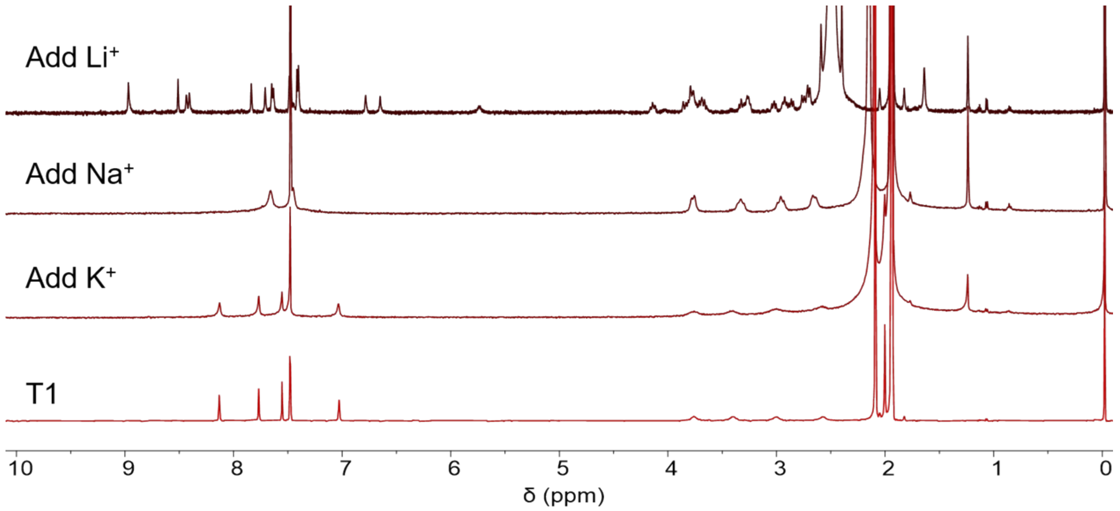

Fig. S60. ^{1}H NMR spectrum (400 MHz, $CDCl_3$:CD_3CN=1:1, 298K) of **T1** with extra amount of M^{+}•BF_4^{-} (M^{+} = Li^{+}, Na^{+}, K^{+}).

After addition of M^{+}•BF_4^{-} (M^{+} = Li^{+}, Na^{+}, K^{+}) into a solution of **T1** in a 1:1 mixture of $CDCl_3$ and CD_3CN, different binding behaviors were observed. In the case of K^{+}•BF_4^{-}, the ^{1}H NMR spectrum of T1 barely changed, indicating that in the presence of CD_3CN, the cage could not recognize K^{+}. In the case of Na^{+}•BF_4^{-}, the ^{1}H NMR spectrum indicated the formation of the binary complex Na^{+}⊂**T1**•BF_4^{-}, in spite of excess amount of Na^{+}•BF_4^{-}. In the case of Li^{+}, a ternary complex $(Li^{+})_2$⊂**T1**•2BF_4^{-} formed.

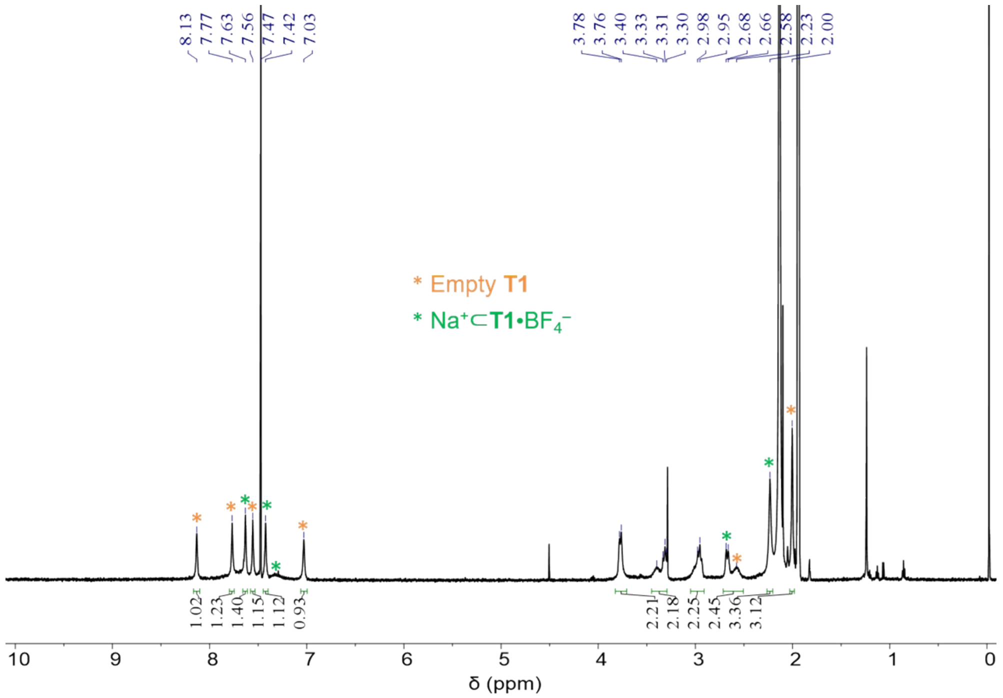

Fig. S61. ^{1}H NMR spectrum of **T1** (8.62×10^{-4} M, $CDCl_3$:CD_3CN=1:1, 298 K) after adding 0.98 equiv. of Na^{+}•BF_4^{-}. The resonances labelled with orange and green stars correspond to **T1** and Na^{+}⊂**T1**•BF_4^{-} respetively. The binding constant K_1 was calculated to be $(2.74\pm0.17)\times10^{3}$, by integrating and comparing the resonances corresponding to **T1** and Na^{+}⊂**T1**•BF_4^{-}. The concentration of "free" Na^{+} was calculated by substracting the concentration of the complex from that of Na^{+}•BF_4^{-} added into the system.

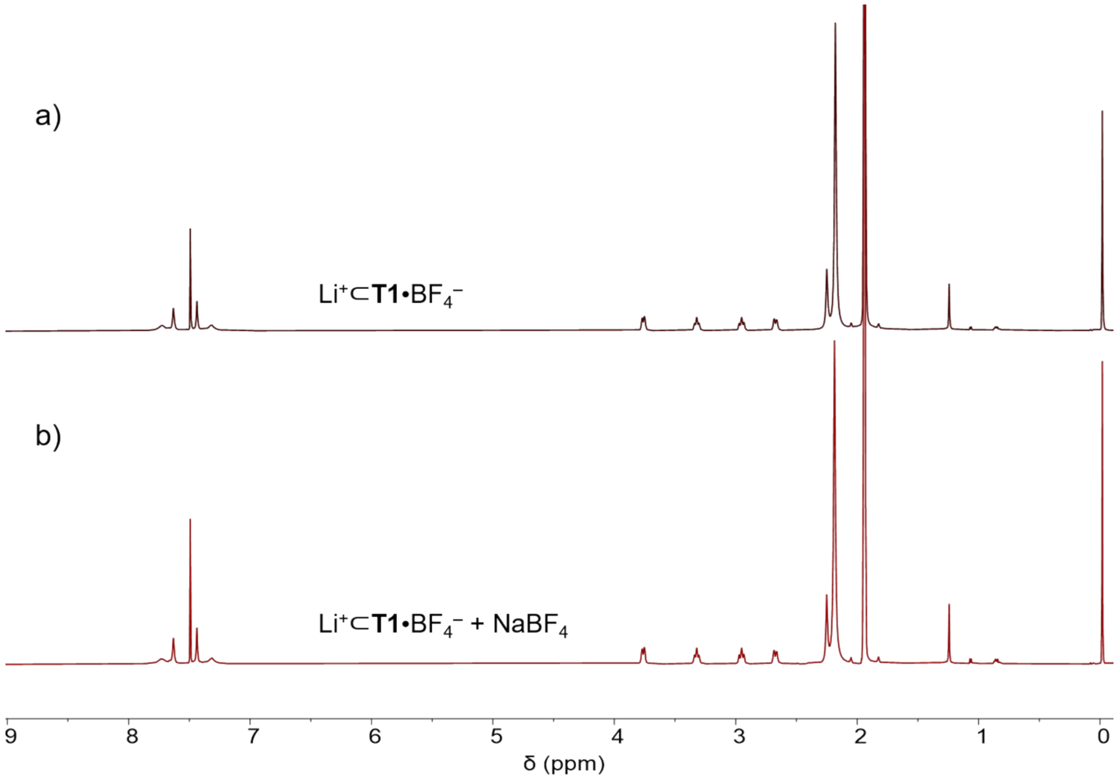

Fig. S62. ^{1}H NMR spectrum (400 MHz, $CDCl_3$:CD_3CN=1:1, 298K) of a 1:1 mixture of Li^+•BF_4^- and **T1**, before a) and after b) adding 5.6 equiv. Na^+•BF_4^- relative to Li^+. All the peaks barely shifted. It indicated that the binding capacity of **T1** to Li^+ is much higher than that of Na^+.

(6) TMEA+ in cage **T1**

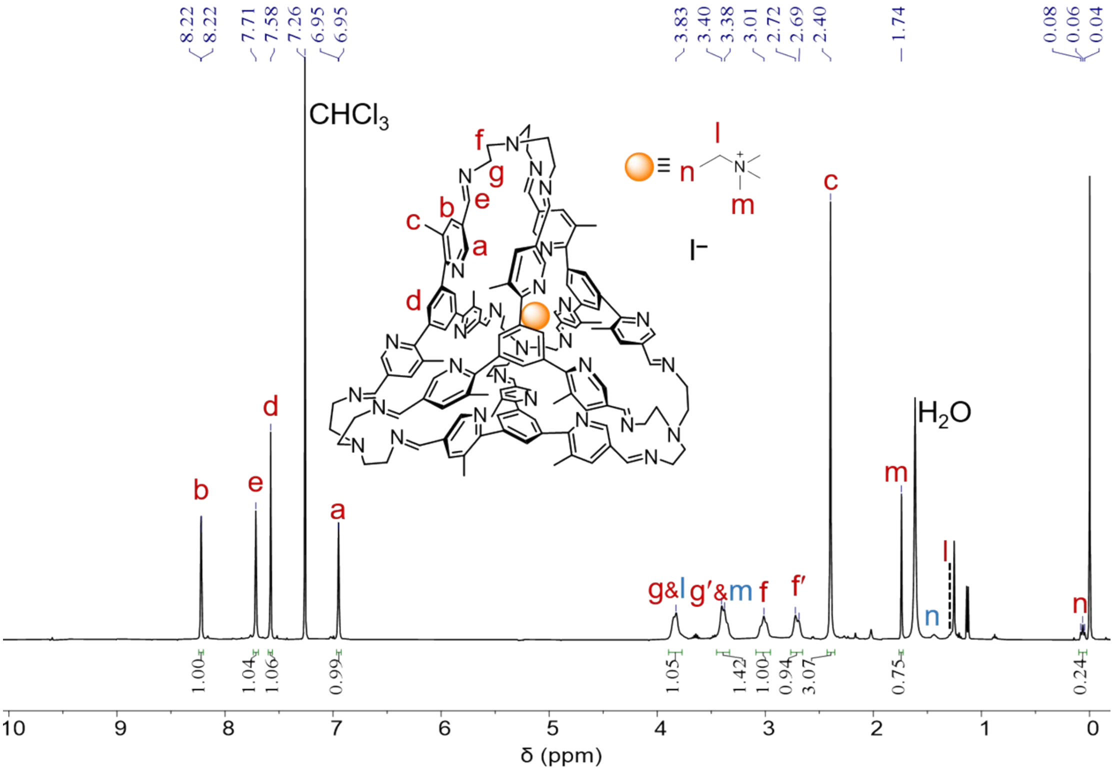

Fig. S63. ^{1}H NMR spectrum of TMEA$^{+}\subset$**T1**•I^{-} (400 MHz, $CDCl_3$, 298 K). The resonances labelled with blue letters *m*, *l* and *n* correspond to the free TMEA^{+}.

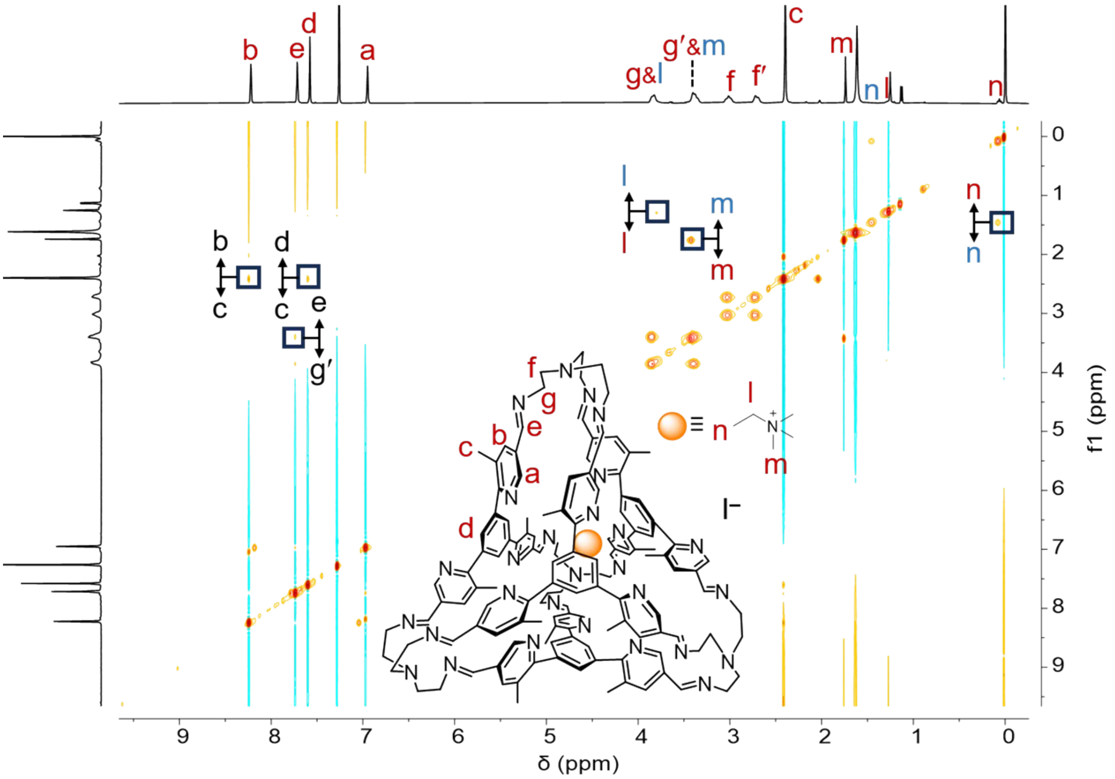

Fig. S64. ^{1}H-^{1}H NOESY spectrum of TMEA$^{+}\subset$**T1**•I^{-} (400 MHz, $CDCl_3$, 298 K). Key correlation peaks are labeled in the spectrum. The resonances labelled with blue letters *m*, *l* and *n* correspond to the free TMEA^{+}.

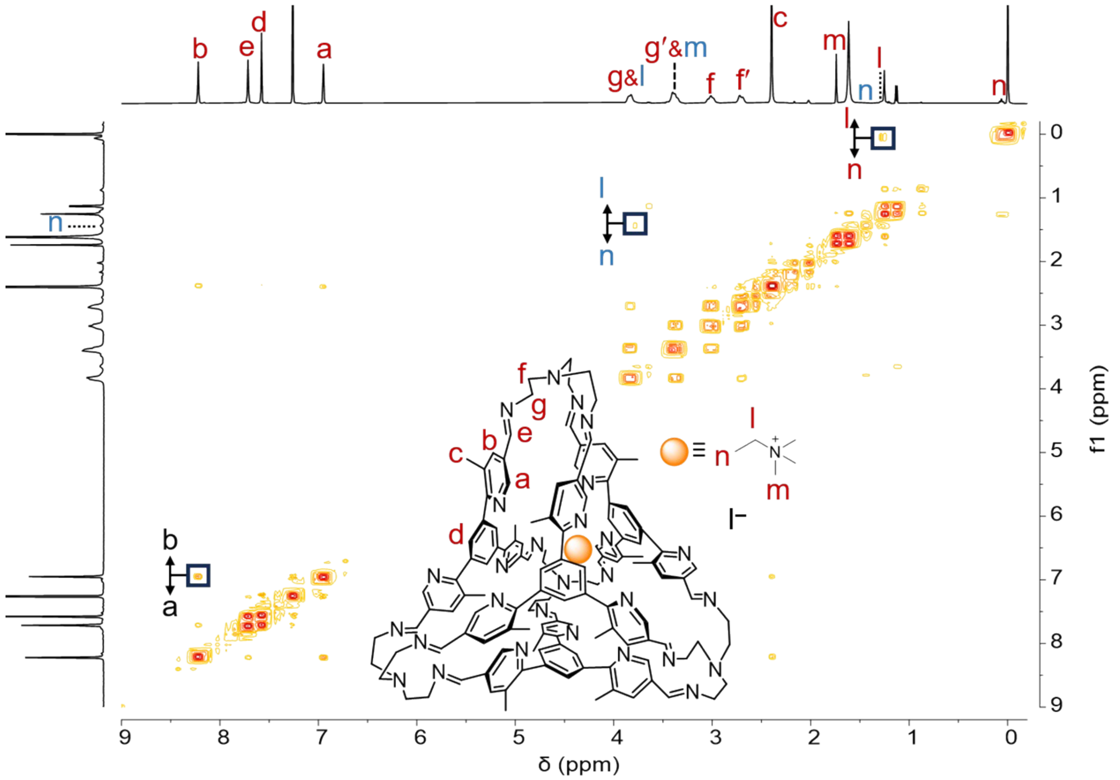

Fig. S65. ^{1}H-^{1}H COSY spectrum of TMEA^{+}⊂**T1**•I^{-} (400 MHz, $CDCl_3$, 298 K). Key correlation peaks are labeled in the spectrum. The resonances labelled with blue letters *m*, *l* and *n* correspond to the free TMEA^{+}.

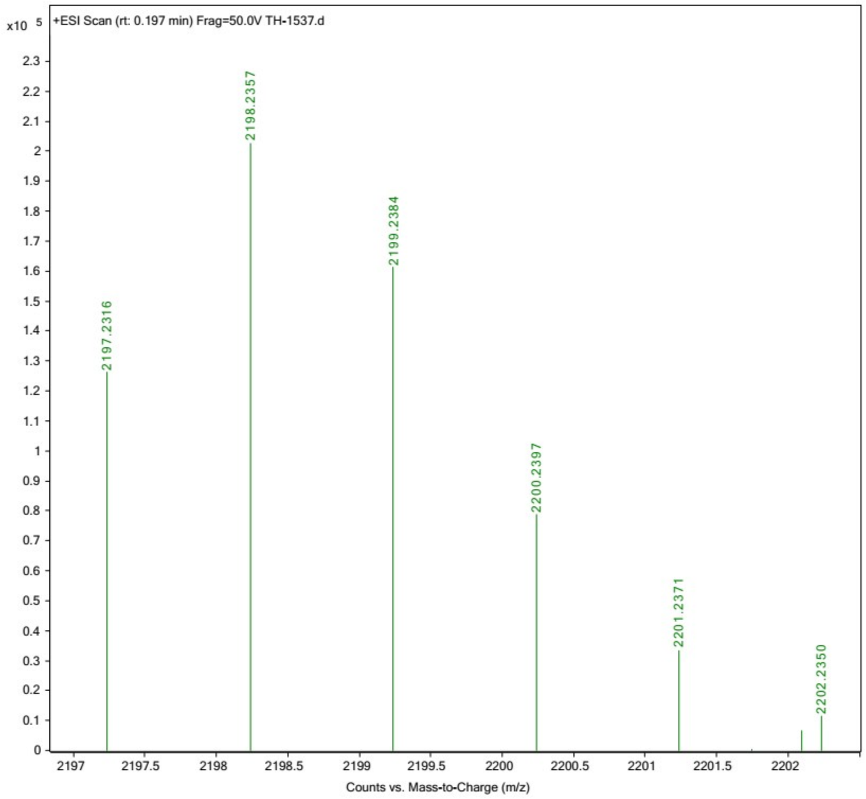

Fig. S66. ESI-HRMS of TMEA^{+}⊂**T1**•I^{-}. ESI-HRMS *m*/*z* calcd for [**T1**+TMEA]$^{+}$ $C_{137}H_{146}N_{29}^{+}$, 2197.2316; found 2197.2316.

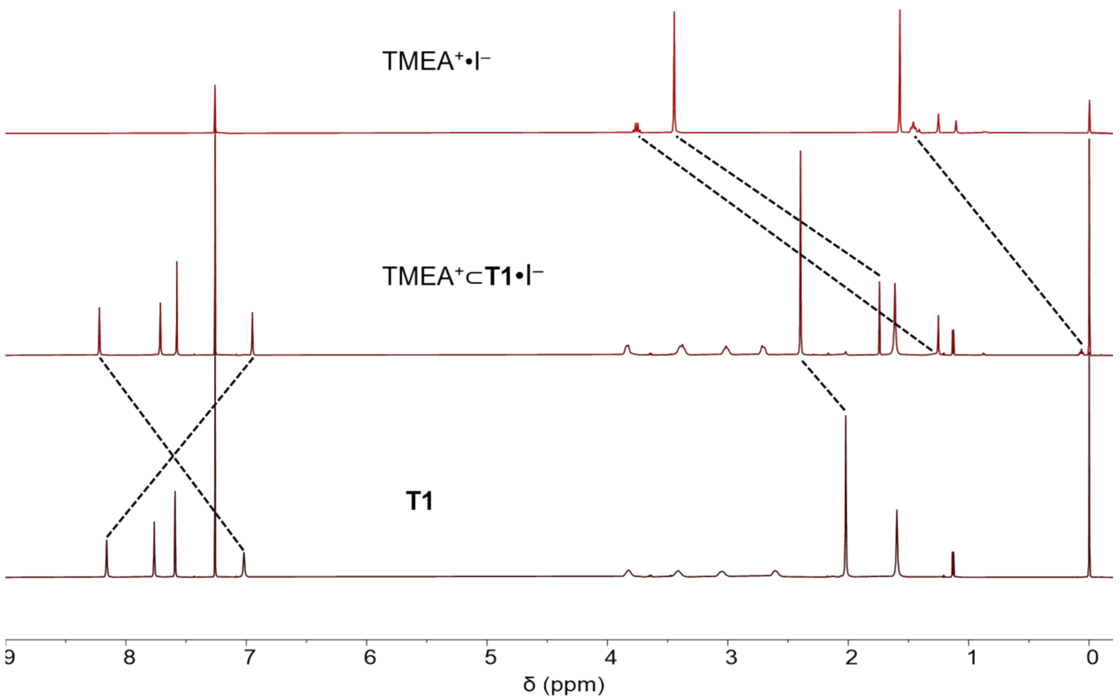

Fig. S67. ^{1}H NMR spectrum (400 MHz, $CDCl_3$, 298 K) of $TMEA^+$•I^- (top), $TMEA^+$⊂**T1**•I^- (middle) and **T1** (bottom).

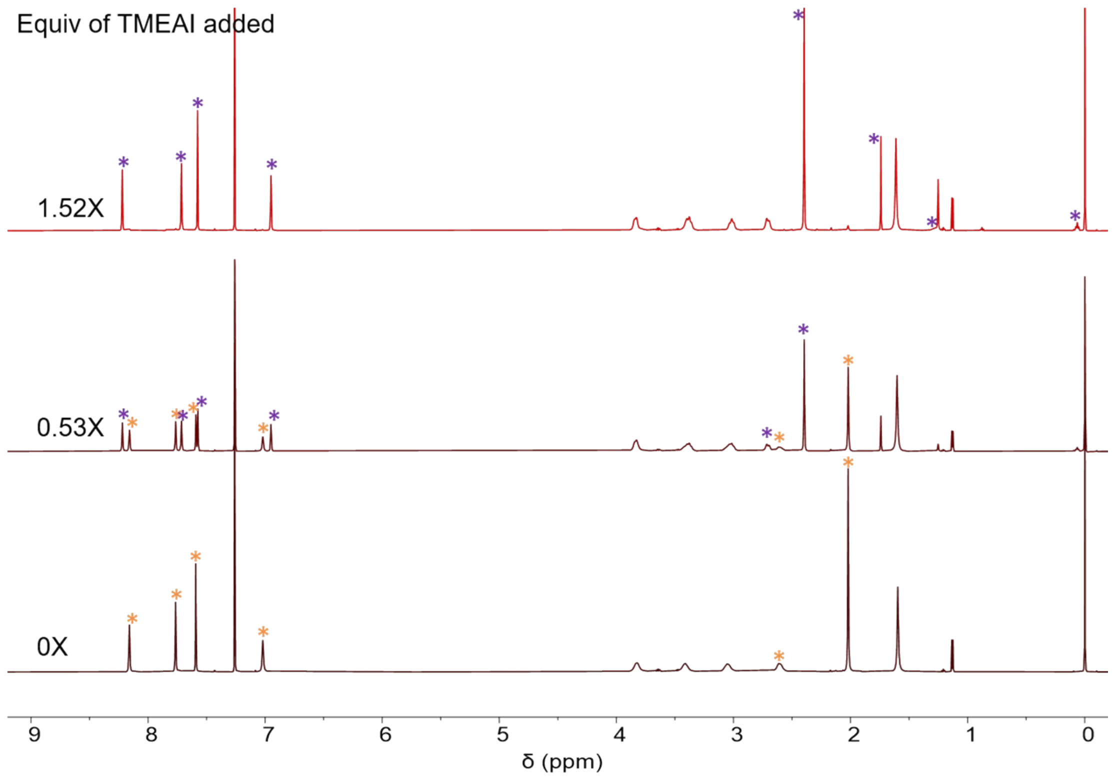

Fig. S68. ^{1}H NMR spectra of **T1** (400 MHz, $CDCl_3$, 298 K) in the presence of 0 (bottom), 0.53 (middle) and 1.52 (top) equiv. of $TMEA^+$•I^-. In the spectra, the resonances labeled with orange and violet stars correspond to the empty cage and the complex, respectively.

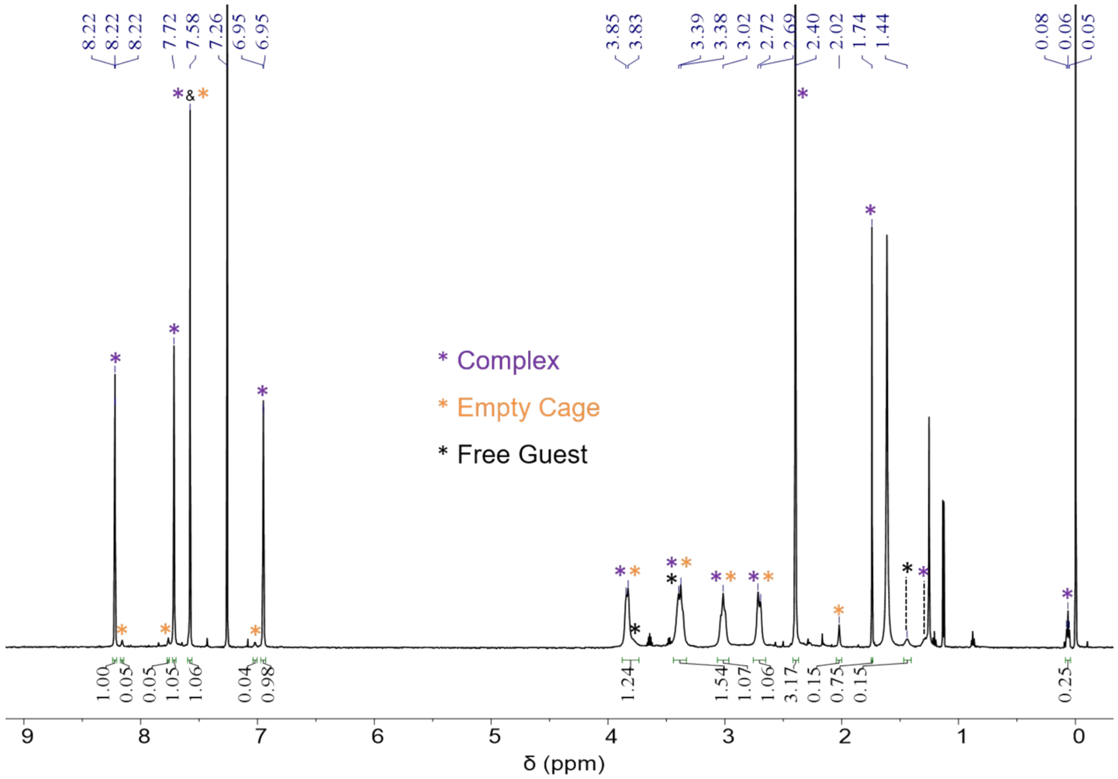

Fig. S69. ^{1}H NMR spectrum of **T1** in the presence of 1.52 equiv. of TMEA$^+$•I$^-$ (400 MHz, $CDCl_3$, 298 K). The resonances labelled with orange, violet and black stars corresponding the empty cage, complex and free guest, respectively. The initial concentration of **T1** is 1.13×10^{-3} M. The binding constant *K*a is calculated to be (3.18±0.12)×10^4 M^{-1}, by integrating and comparing the resonances corresponding to the complex, free guest and empty cage.

(7) choline in cage **T1**

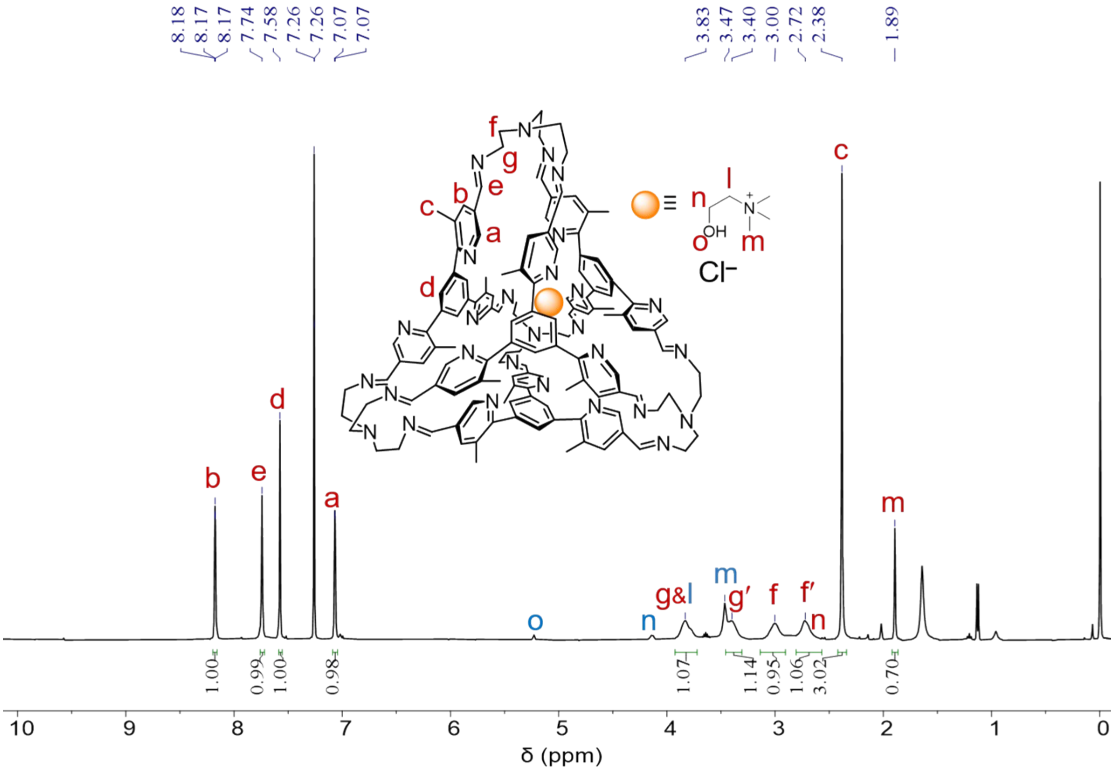

Fig. S70. ^{1}H NMR spectrum of choline$^{+}\subset$**T1**•Cl^{-} (400 MHz, $CDCl_3$, 298 K). The resonances labelled with blue letters *m*, *l*, *n* and *o* correspond to the free choline^{+}.

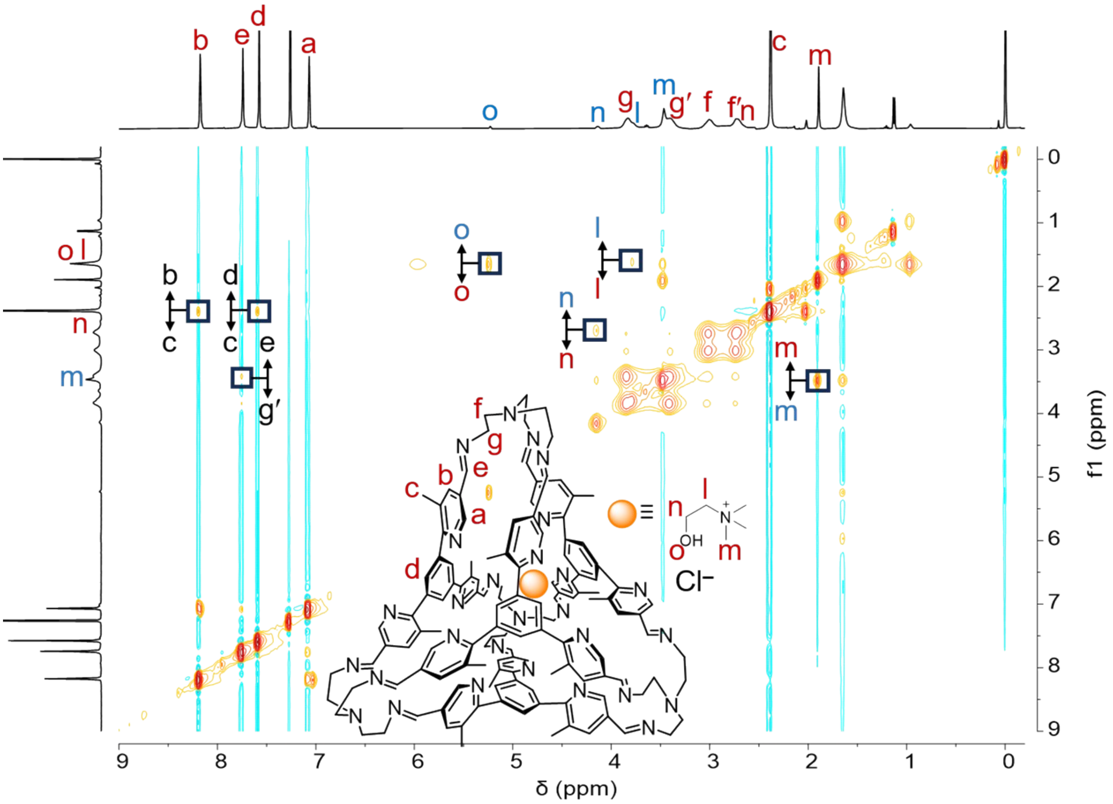

Fig. S71. ^{1}H-^{1}H NOESY spectrum of choline$^{+}\subset$**T1**•Cl^{-} (400 MHz, $CDCl_3$, 298 K). The resonances labelled with blue letters *m*, *l*, *n* and *o* correspond to the free choline^{+}. Key correlation peaks are labeled in the spectrum.

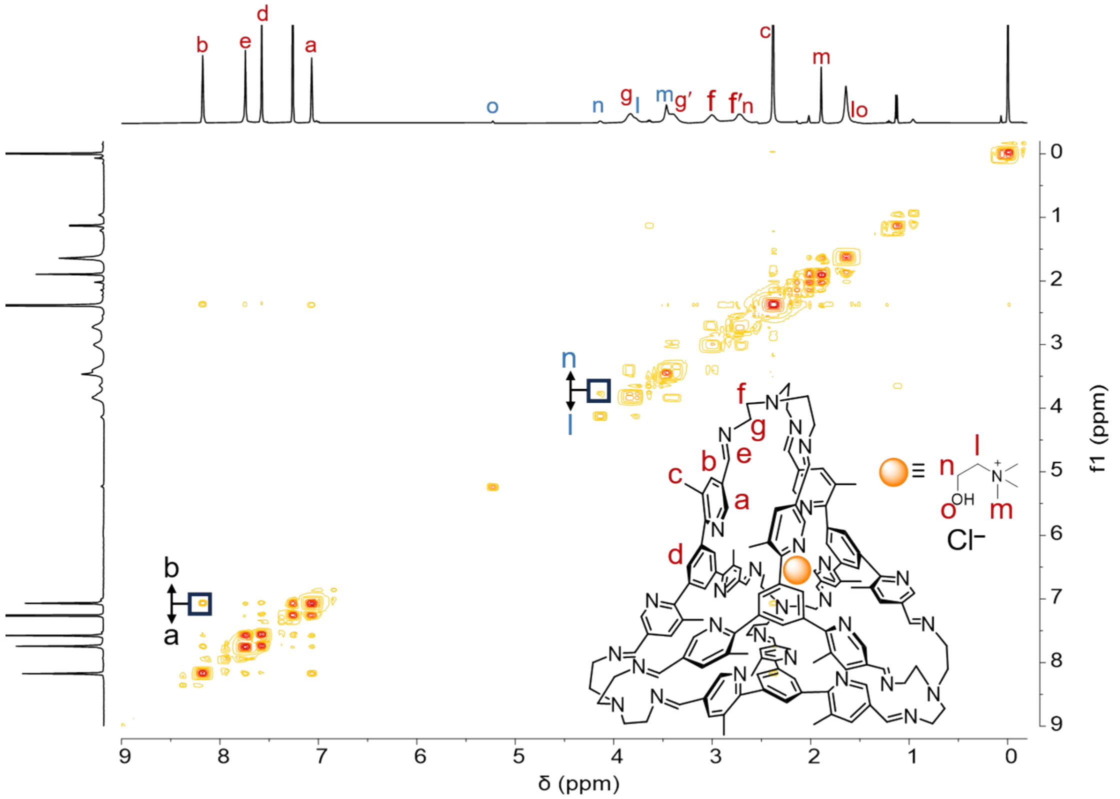

Fig. S72. ^1H-^1H COSY spectrum of choline$^+$⊂**T1**•Cl$^-$ (400 MHz, $CDCl_3$, 298 K). The resonances labelled with blue letters *m*, *l*, *n* and *o* correspond to the free choline$^+$. Key correlation peaks are labeled in the spectrum.

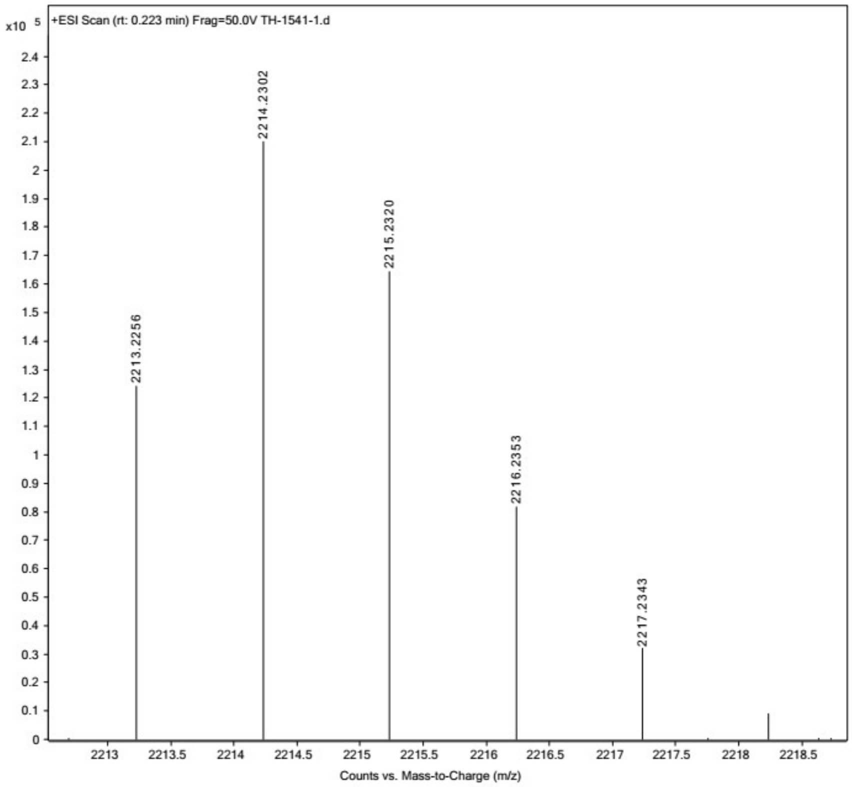

Fig. S73. ESI-HRMS of choline$^+$⊂**T1**•Cl$^-$. ESI-HRMS *m*/*z* calcd for [**T1**+choline]$^+$ $C_{137}H_{146}N_{29}O^+$, 2213.2265; found 2213.2256.

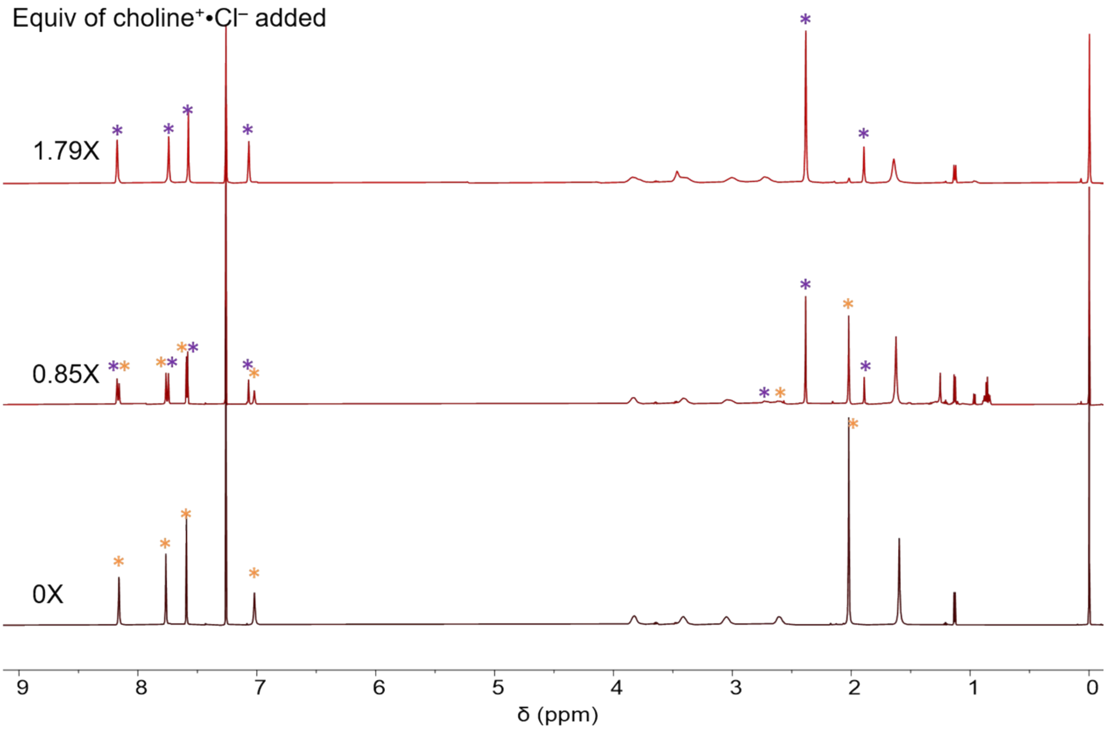

Fig. S74. ^{1}H NMR spectra of **T1** (400 MHz, $CDCl_3$, 298 K) in the presence of 0 (bottom), 0.85 (middle) and 1.79 (top) equiv. of choline^{+}•Cl^{-}. In the spectra, the resonances labeled with orange and violet stars correspond to the empty cage and the complex, respectively.

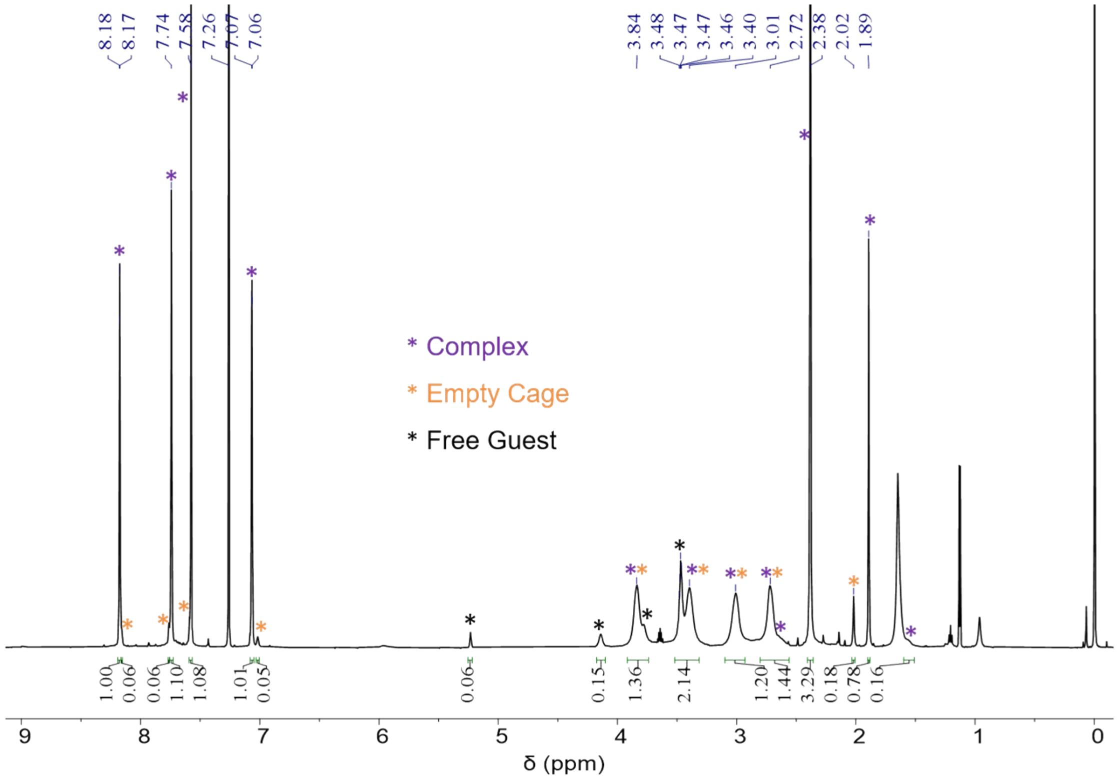

Fig. S75. ^{1}H NMR spectrum of **T1** in the presence of 1.79 equiv. of choline$^+$•Cl$^-$ (400 MHz, $CDCl_3$, 298 K). The resonances labelled with orange, violet and black stars corresponding the empty cage, complex and free guest, respectively. The initial concentration of **T1** is 1.45×10^{-3} M. The binding constant *K*a is calculated to be (1.42±0.08)×10^4 M^{-1}, by integrating and comparing the resonances corresponding to the complex, free guest and empty cage.

(8) chlormequat in cage **T1**

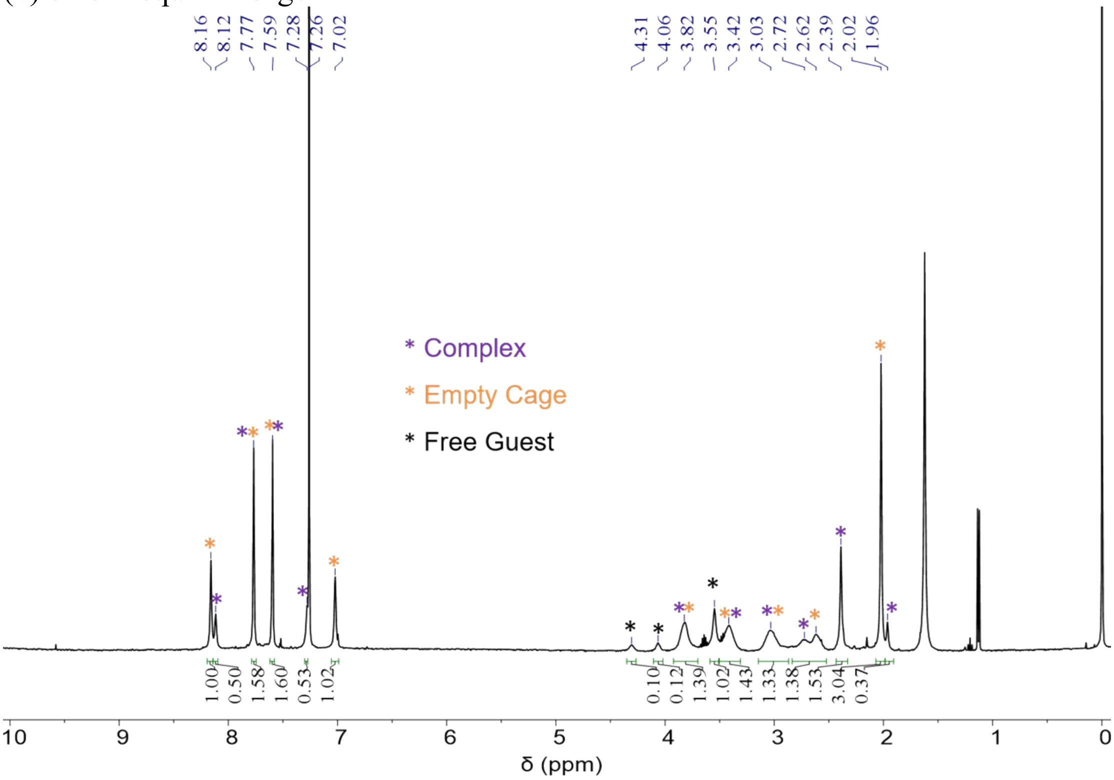

Fig. S76. ^{1}H NMR spectrum of **T1** in the presence of 0.73 equiv. of chlormequat^{+}•Cl^{-} (400 MHz, $CDCl_3$, 298 K). The resonances labelled with orange, violet and black stars corresponding the empty cage, complex and free guest, respectively. The initial concentration of **T1** is 1.12×10^{-3} M. The binding constant *K*a is calculated to be $(1.02\pm0.12)\times10^{3}$ M^{-1}, by integrating and comparing the resonances corresponding to the complex, free guest and empty cage.

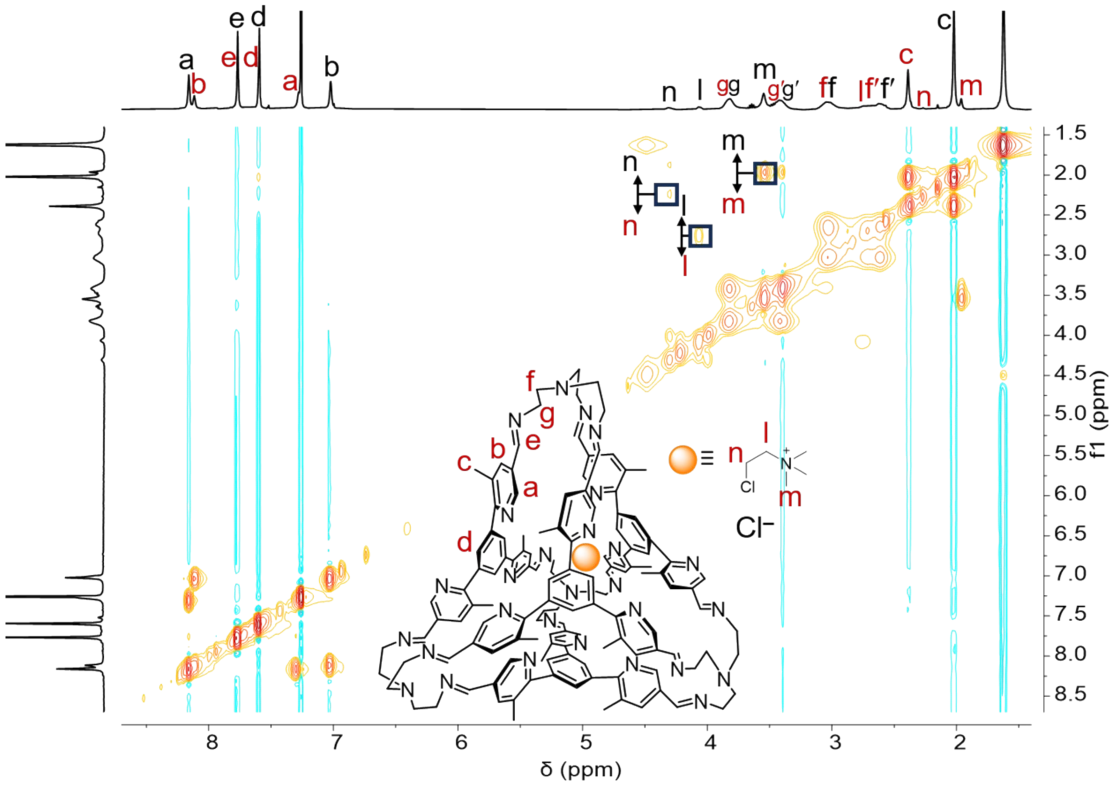

Fig. S77. ^{1}H-^{1}H NOESY spectrum of **T1** in the presence of 0.73 equiv. of chlormequat$^+$•Cl$^-$ (400 MHz, $CDCl_3$, 298 K). The resonances labelled with black letters *m*, *l* and *n* correspond to the free choline$^+$. Key correlation peaks are labeled in the spectrum.

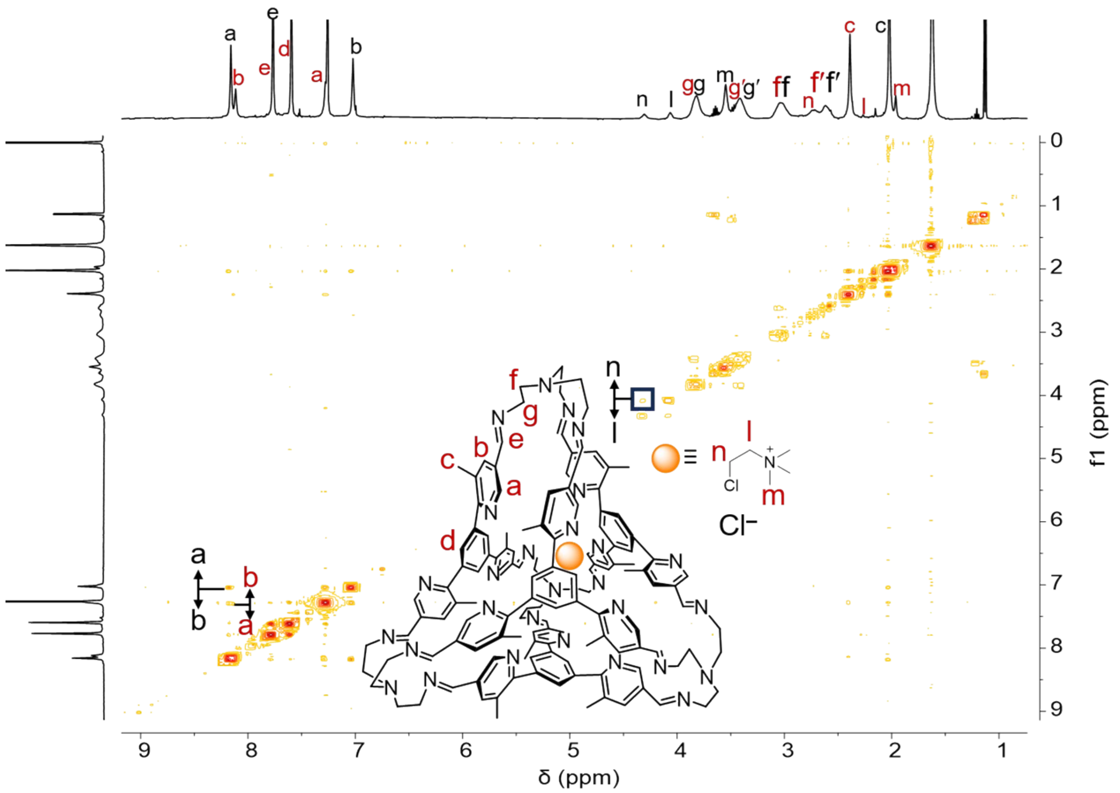

Fig. S78. ^{1}H-^{1}H COSY spectrum of **T1** in the presence of 0.73 equiv. of chlormequat$^+$•Cl$^-$ (400 MHz, $CDCl_3$, 298 K). The resonances labelled with black letters *m*, *l* and *n* correspond to the free choline$^+$. Key correlation peaks are labeled in the spectrum.

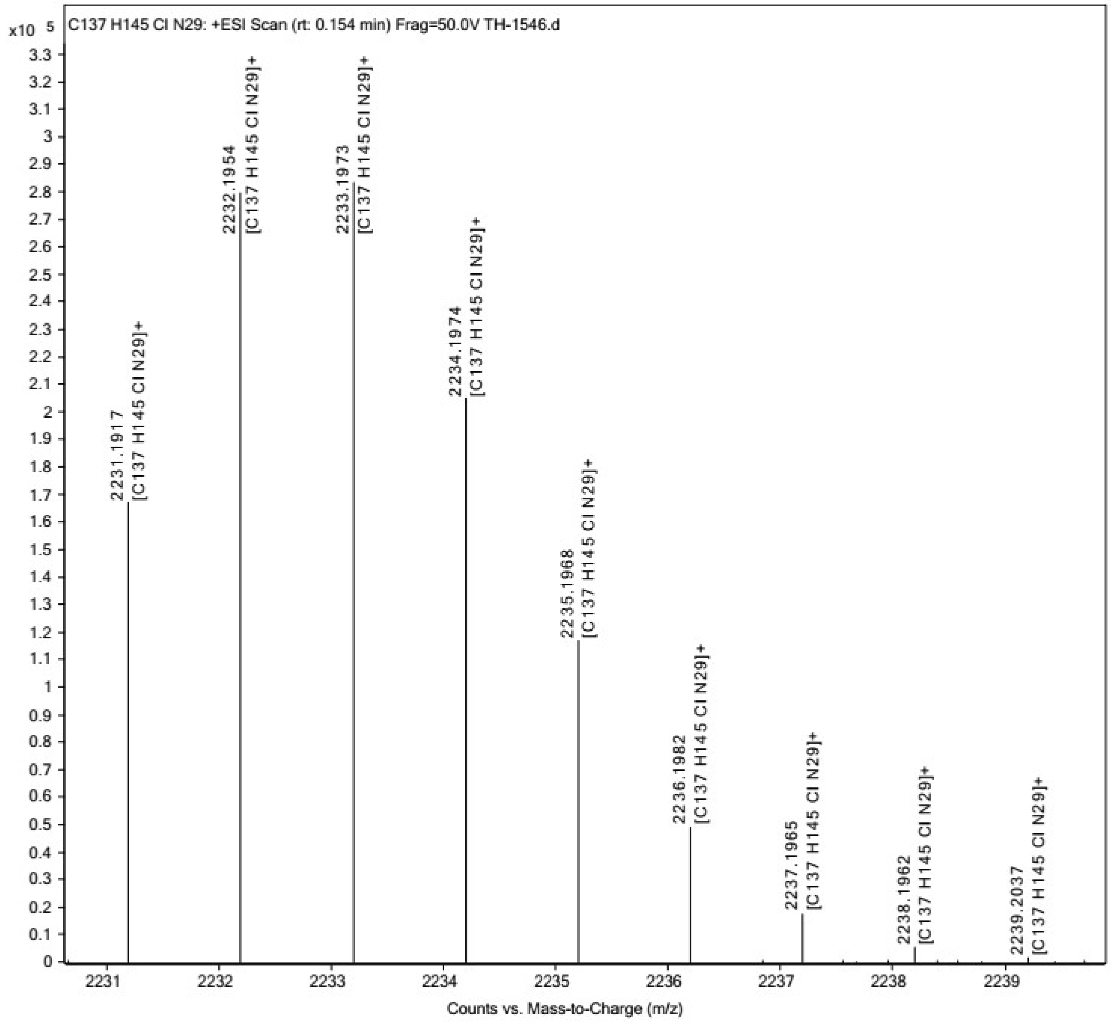

Fig. S79. ESI-HRMS of chlormequat$^+$⊂**T1**•Cl$^-$. ESI-HRMS *m*/*z* calcd for [**T1**+chlormequat]$^+$ $C_{137}H_{145}N_{29}Cl^+$, 2231.1926; found 2231.1917.

(9) trimethylamine N-oxide in cage **T1**

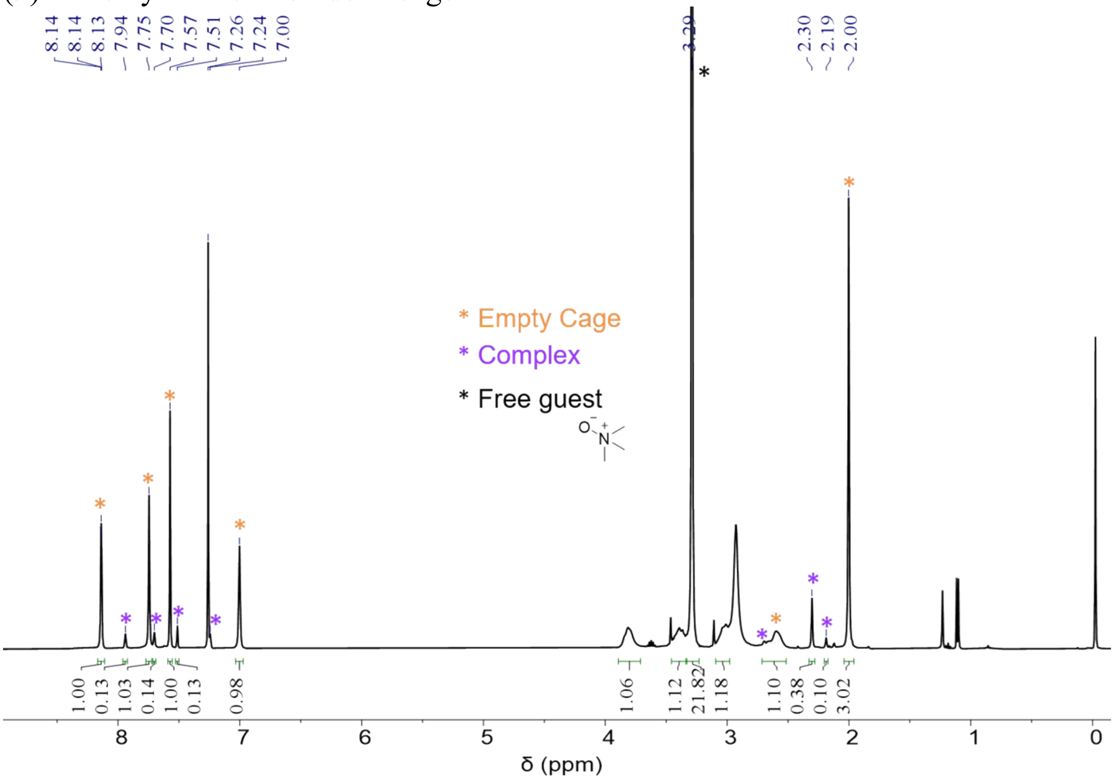

Fig. S80. ^{1}H NMR spectrum of **T1** in the presence of 26 equiv. of trimethylamine N-oxide (400 MHz, $CDCl_3$, 298 K). The resonances labelled with orange, violet and black stars corresponding the empty cage, complex and free guest, respectively. The initial concentration of **T1** is 2.24×10^{-3} M. The binding constant *K*a is calculated to be 2.22±0.06 M^{-1}, by integrating and comparing the resonances corresponding to the complex, free guest and empty cage.

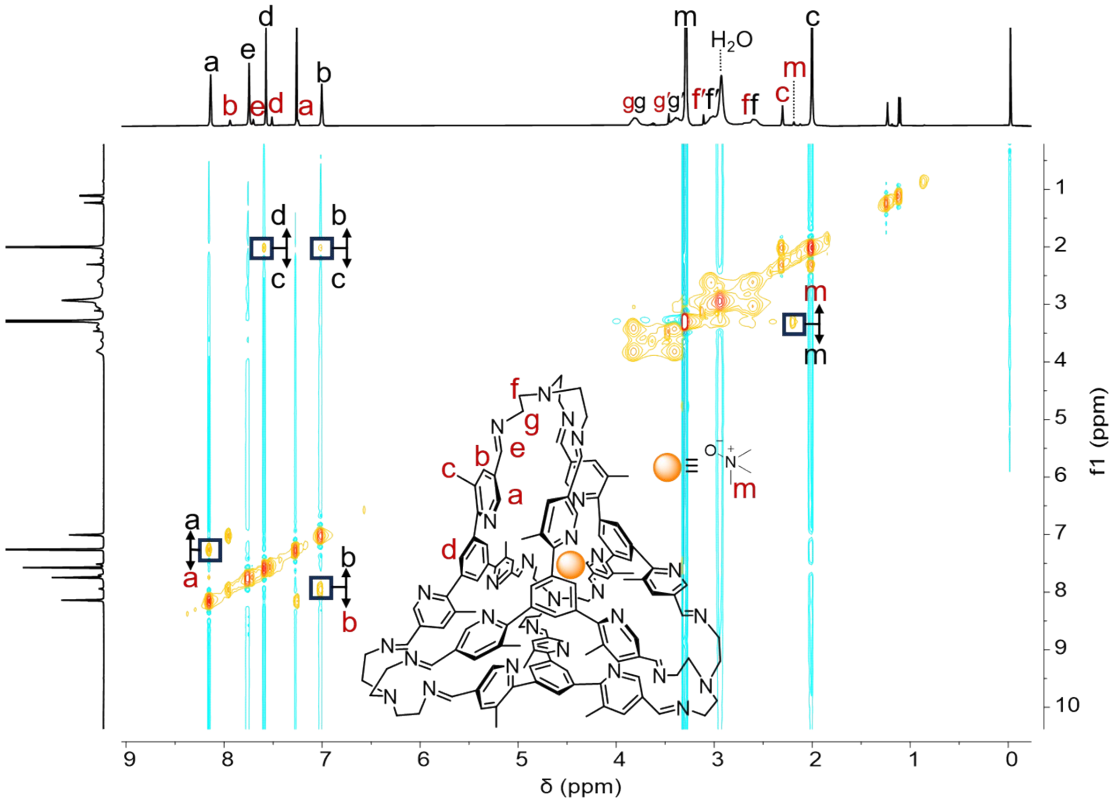

Fig. S81. ^{1}H-^{1}H NOESY spectrum of **T1** in the presence of 26 equiv. of trimethylamine N-oxide (400 MHz, $CDCl_3$, 298 K). The resonances labelled with black letters *a*-*g* and *m* correspond to the empty cage **T1** and free trimethylamine N-oxide. Key correlation peaks are labeled in the spectrum.

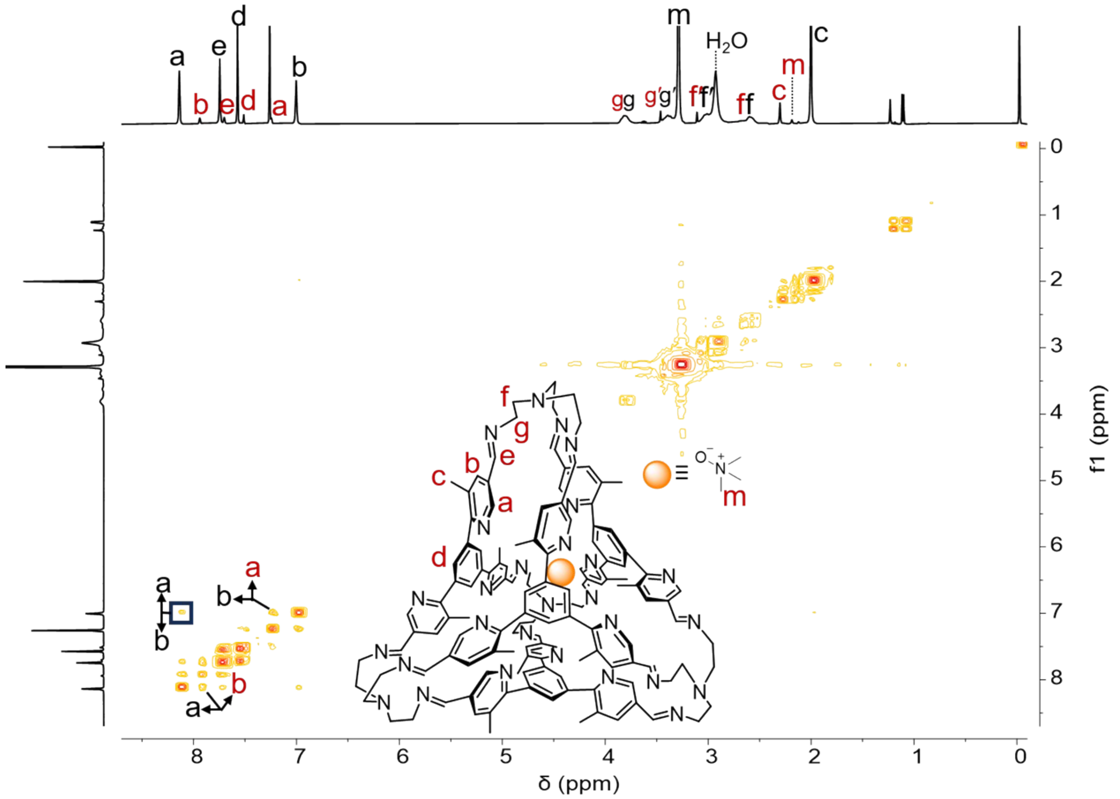

Fig. S82. ^{1}H-^{1}H COSY spectrum of **T1** in the presence of 26 equiv. of trimethylamine N-oxide (400 MHz, $CDCl_3$, 298 K). The resonances labelled with black letters *a*-*g* and *m* correspond to the empty cage **T1** and free trimethylamine N-oxide. Key correlation peaks are labeled in the spectrum.

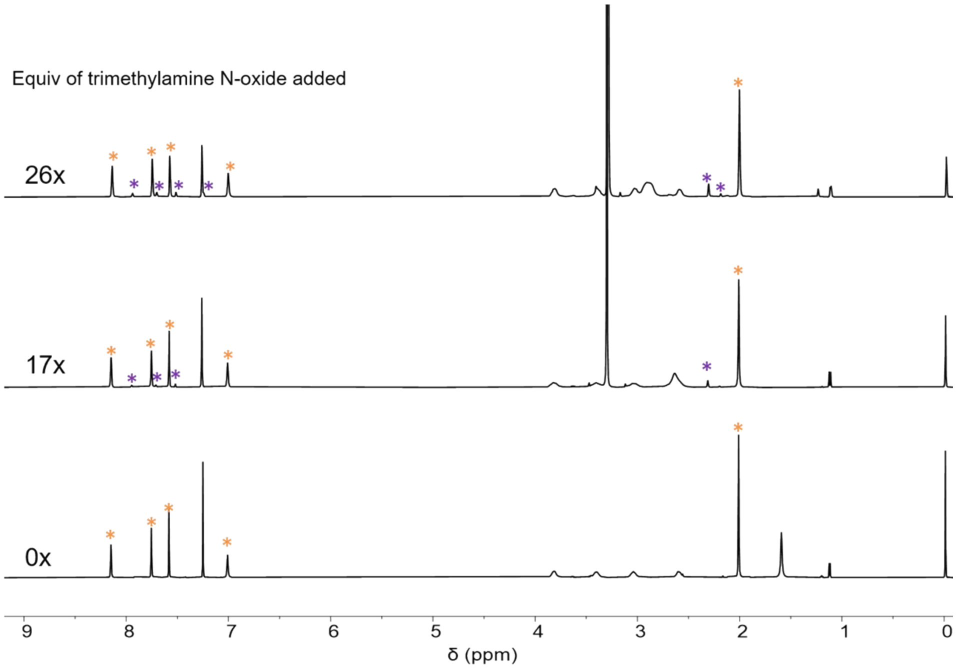

Fig. S83. ^{1}H NMR spectra (600 MHz, $CDCl_3$, 298 K) of **T1** after addition of different amout of trimethylamine N-oxide. The resonances that are labelled with orange and violet stars correspond the **T1** and trimethylamine N-oxide⊂**T1**, respectively. The concentration of the **T1** is 2.24×10^{-3} M for all the NMR samples.

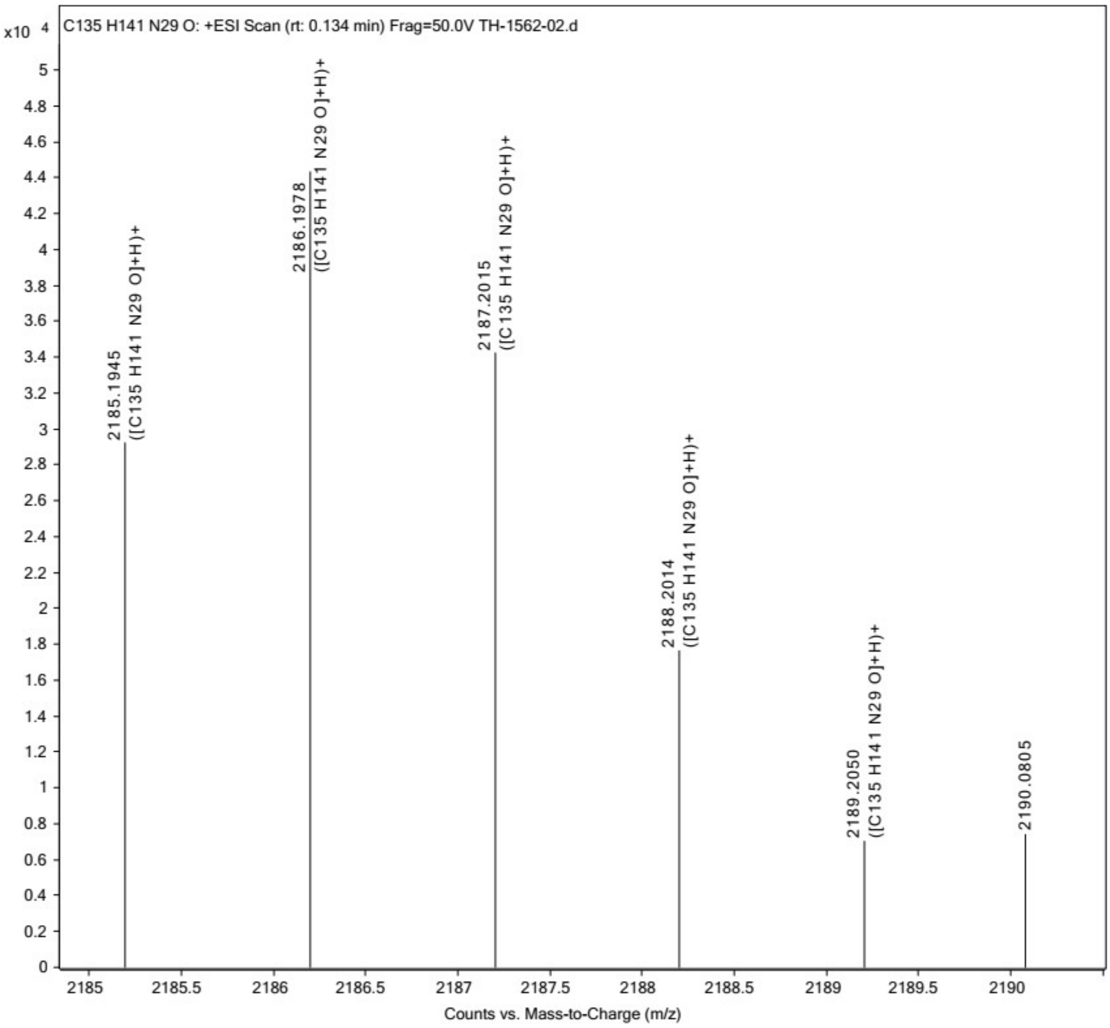

Fig. S84. ESI-HRMS of trimethylamine N-oxide⊂**T1**. ESI-HRMS *m*/*z* calcd for [**T1**+trimethylamine N-oxide+H]$^+$ $C_{135}H_{142}N_{29}O^+$, 2185.1947; found 2185.1945.

(10) Mepiquat chloride in cage **T1**

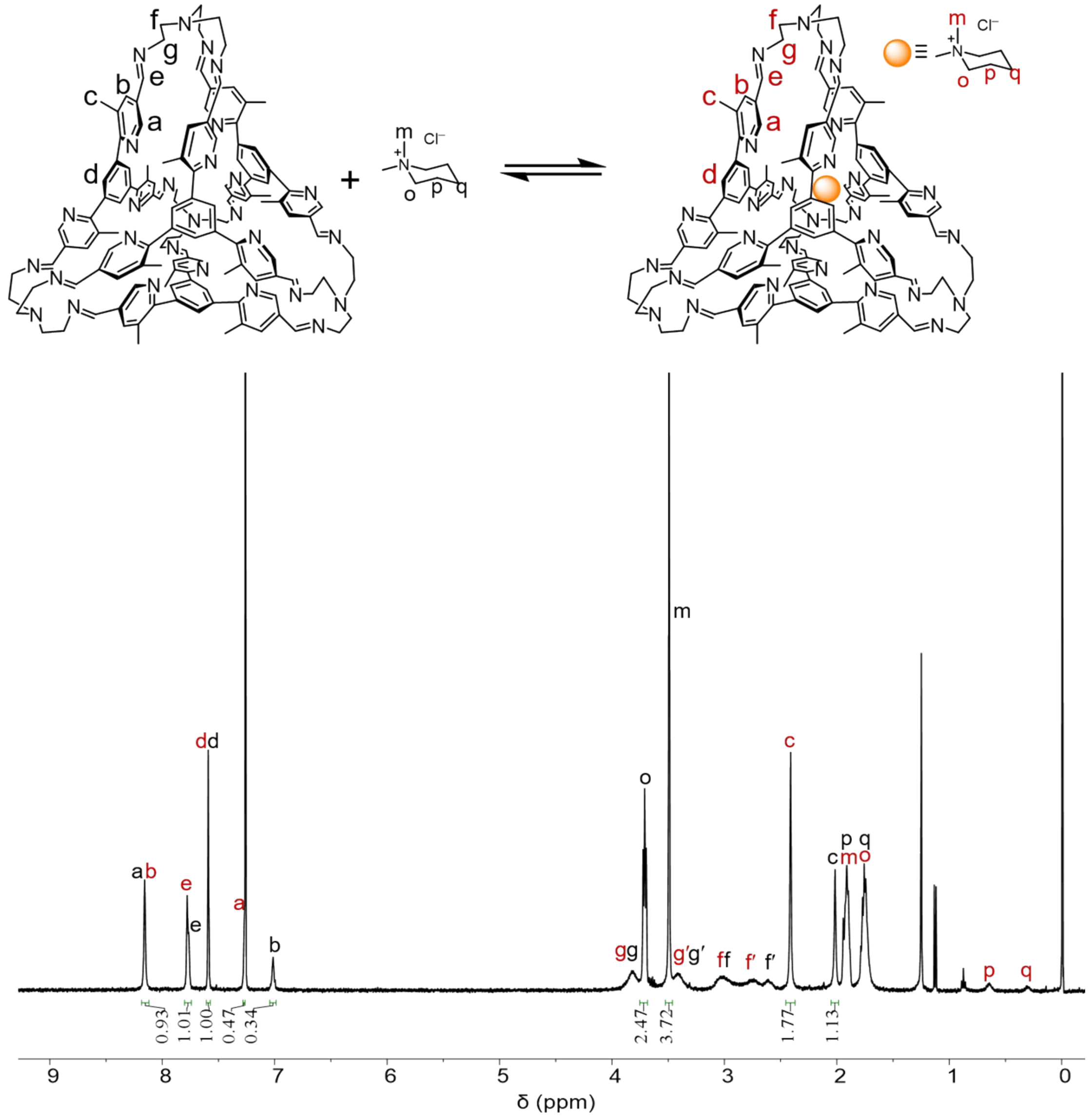

Fig. S85. ^{1}H NMR spectrum of mepiquat$^{+}\subset$**T1**•Cl^{-} (400 MHz, $CDCl_3$, 298 K). The resonances labelled with black letters *a-g* and *m-q* correspond to the empty cage **T1** and free mepiquat.

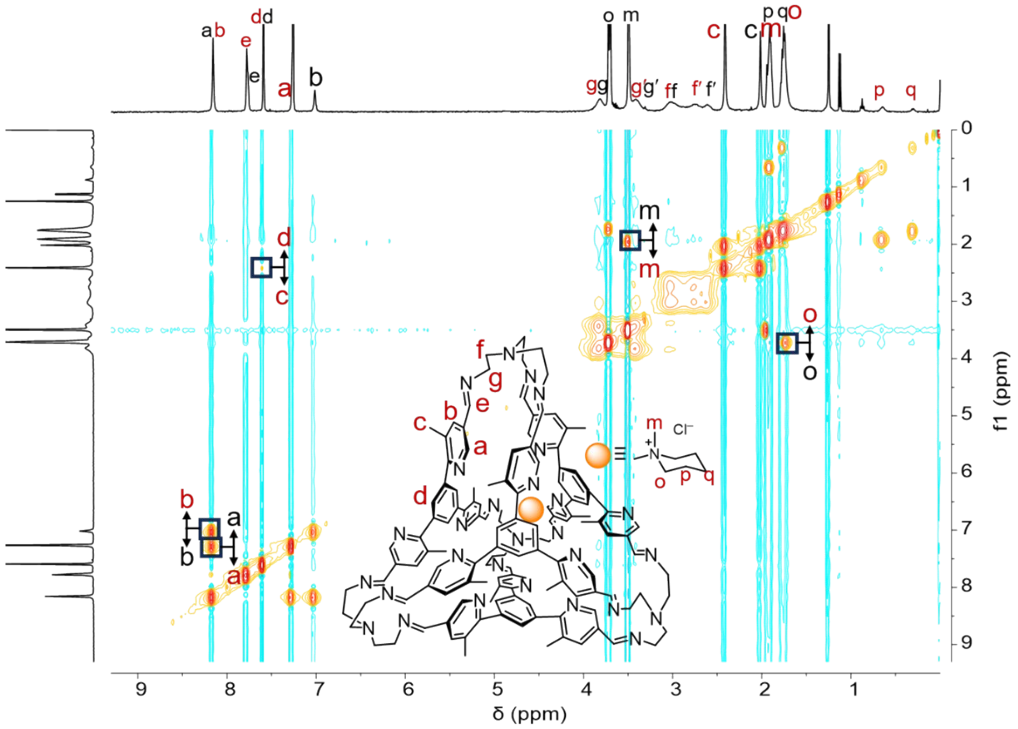

Fig. S86. ^{1}H-^{1}H NOESY spectrum of mepiquat^{+}⊂**T1**•Cl^{-} (400 MHz, $CDCl_3$, 298 K). The resonances labelled with black letters *a-g* and *m-q* correspond to the empty cage **T1** and free mmepiquat. Key correlation peaks are labeled in the spectrum.

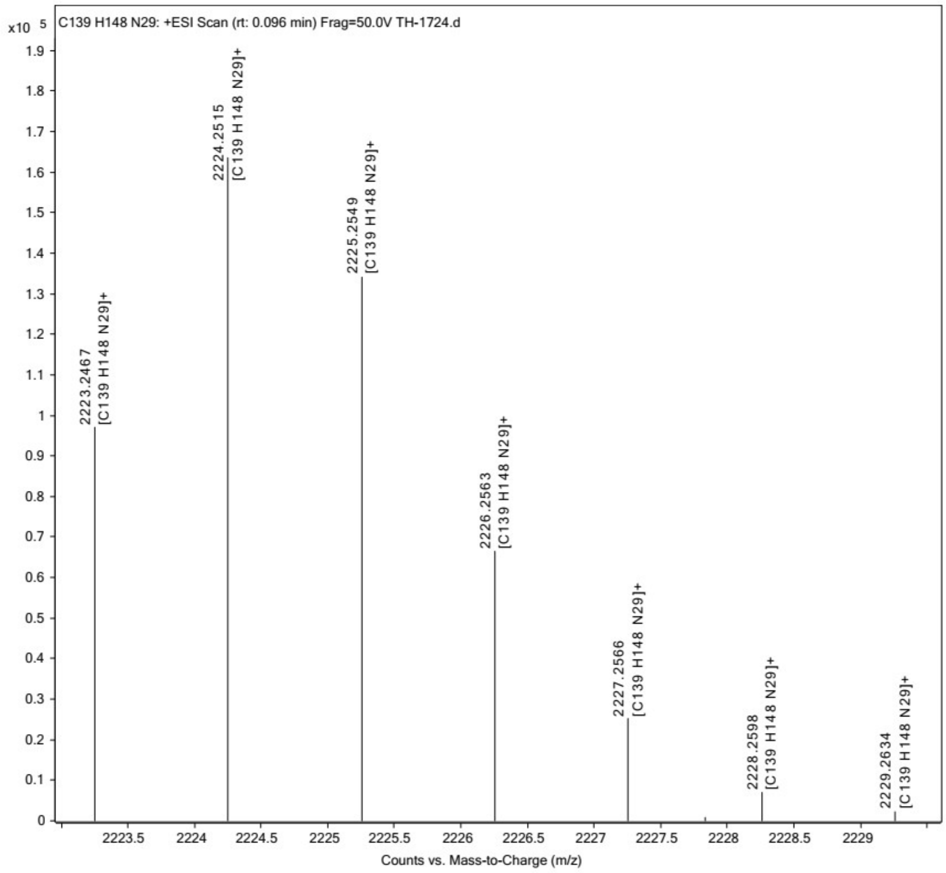

Fig. S87. ESI-HRMS of mepiquat^{+}⊂**T1**•Cl^{-}. ESI-HRMS *m*/*z* calcd for [**T1**+mepiquat]$^{+}$ $C_{139}H_{148}N_{29}^{+}$, 2223.2467; found 2223.2467.

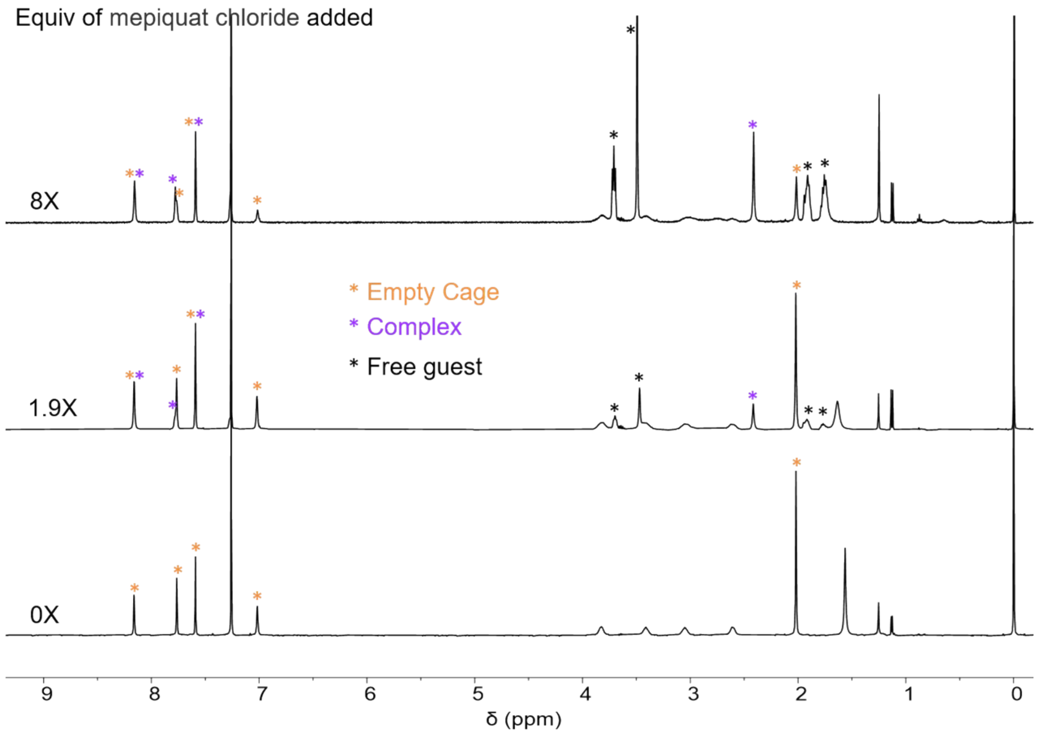

Fig. S88. ^{1}H NMR spectra of **T1** (400 MHz, $CDCl_3$, 298 K) in the presence of 0 (bottom), 1.9 (middle) and 8.0 (top) equiv. of mepiquat^{+}•Cl^{-}. In the spectra, the resonances labeled with orange and violet stars correspond to the "empty" cage and the complex, respectively.

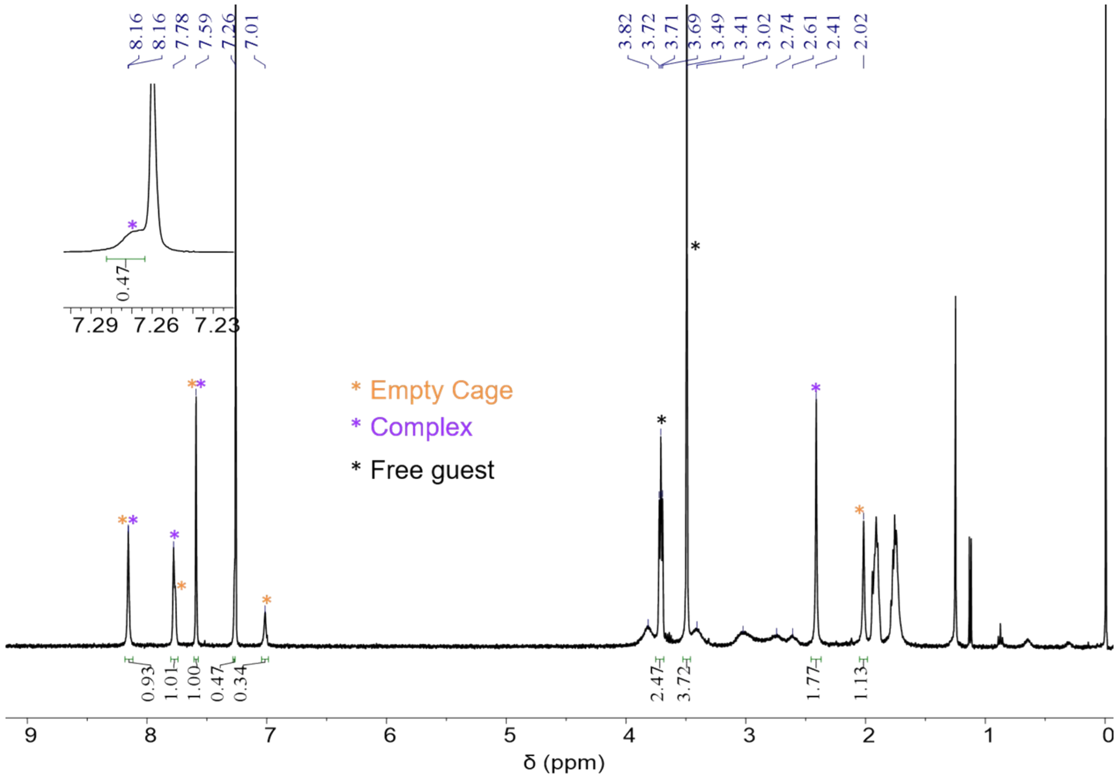

Fig. S89. ^{1}H NMR spectrum of **T1** in the presence of 8.0 equiv. of mepiquat^{+}•Cl^{-}. The resonances labelled with orange, violet and black stars corresponding the empty cage, complex and free guest, respectively. The initial concentration of **T1** is 1.17 $\times 10^{-3}$ M. The binding constant *K*a is calculated to be $(2.04\pm0.27)\times10^{2}$ M^{-1} , by integrating and comparing the resonances corresponding to the complex, free guest and empty cage.

(11) Trimethylamine hydrochloride (TMAH$^+$Cl$^-$) in cage **T1**

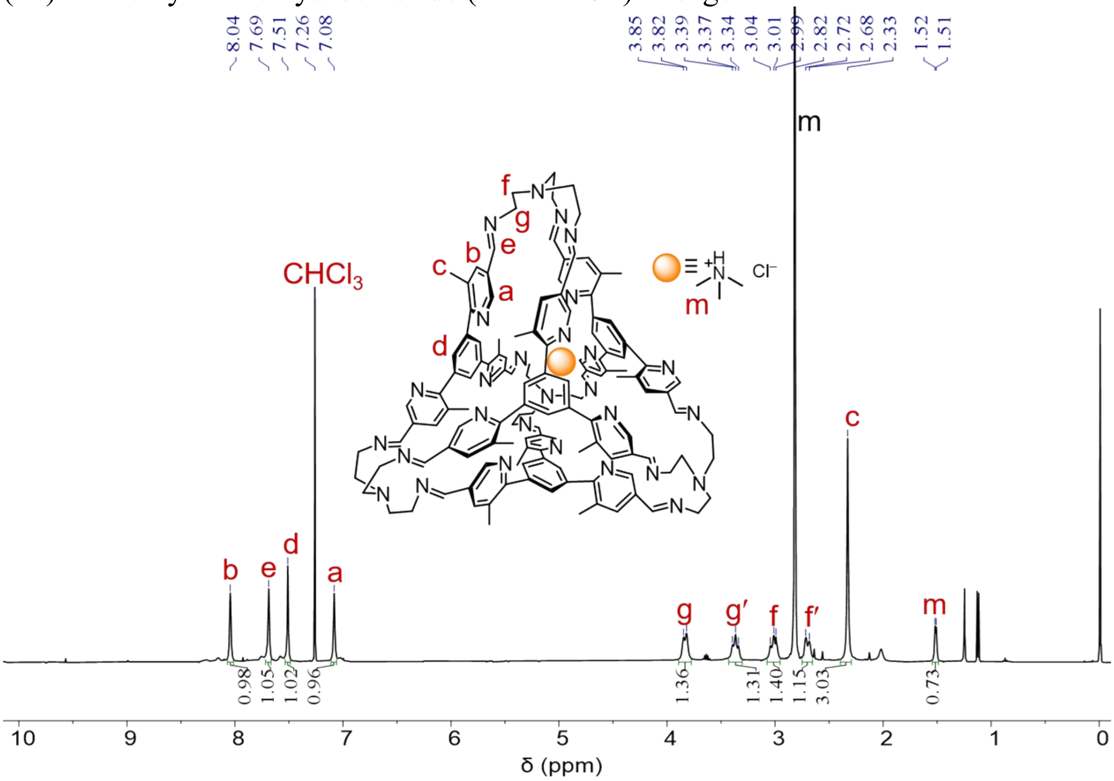

Fig. S90. ^{1}H NMR spectrum of TMAH$^+$⊂**T1**•Cl$^-$ (400 MHz, CDCl$_3$, 298 K). The resonances labelled with black letter *m* correspond to the free TMAH$^+$.

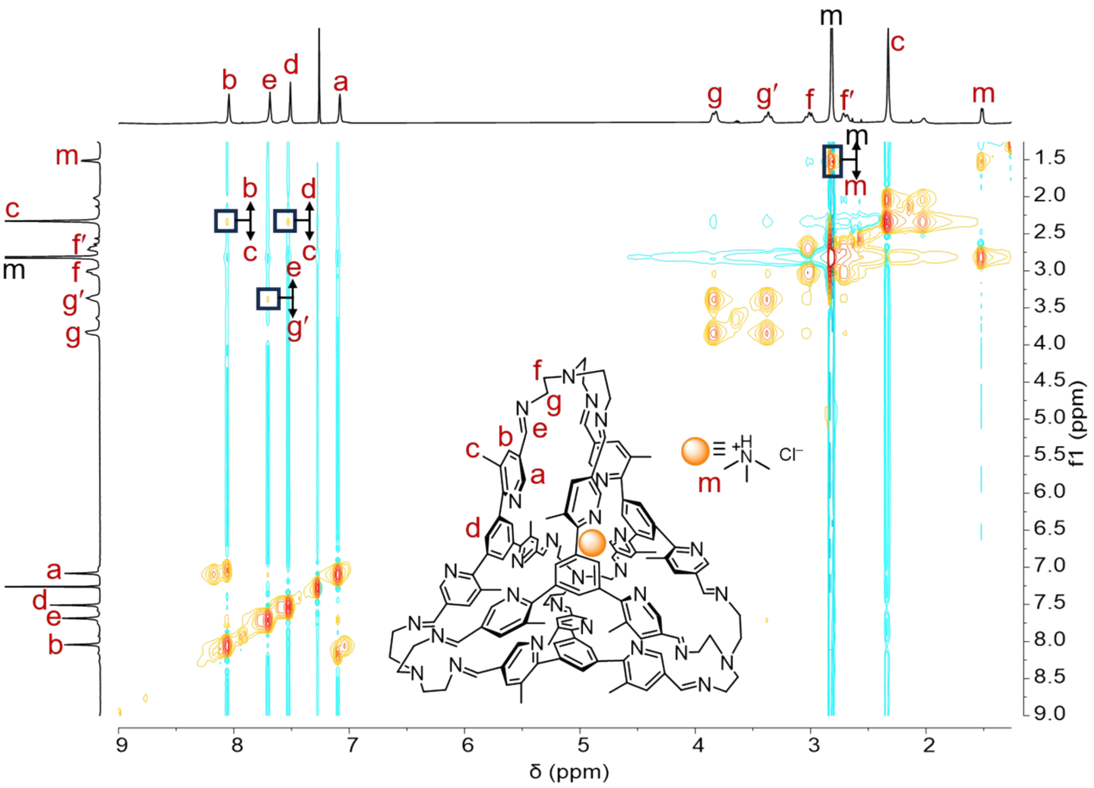

Fig. S91. ^{1}H-^{1}H NOESY spectrum of TMAH$^+$⊂**T1**•Cl$^-$ (400 MHz, CDCl$_3$, 298 K). Key correlation peaks are labeled in the spectrum.

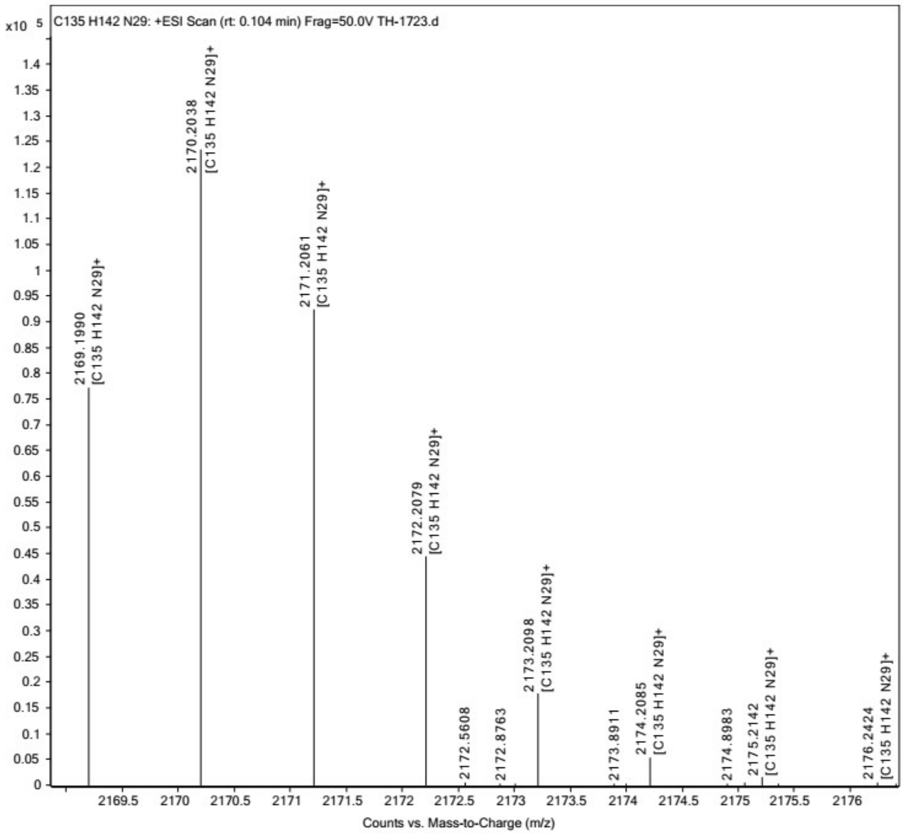

Fig. S92. ESI-HRMS of TMAH$^+$⊂**T1**•Cl$^-$. ESI-HRMS *m*/*z* calcd for [**T1**+TMAH]$^+$ $C_{135}H_{142}N_{29}^+$, 2169.1998; found 2169.1990.

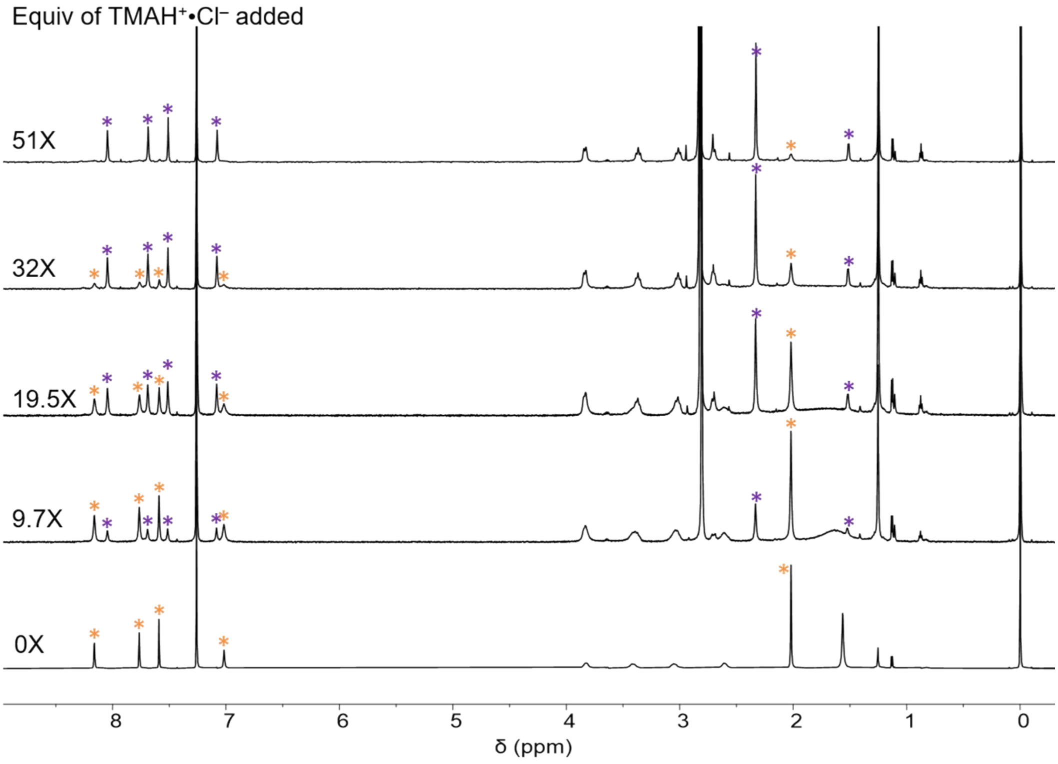

Fig. S93. ^{1}H NMR spectra of **T1** (400 MHz, $CDCl_3$, 298 K) in the presence of 0 (bottom) to 51 (top) equiv. of TMAH$^+$•Cl$^-$. In the spectra, the resonances labeled with orange and violet stars correspond to the "empty" cage and the complex, respectively.

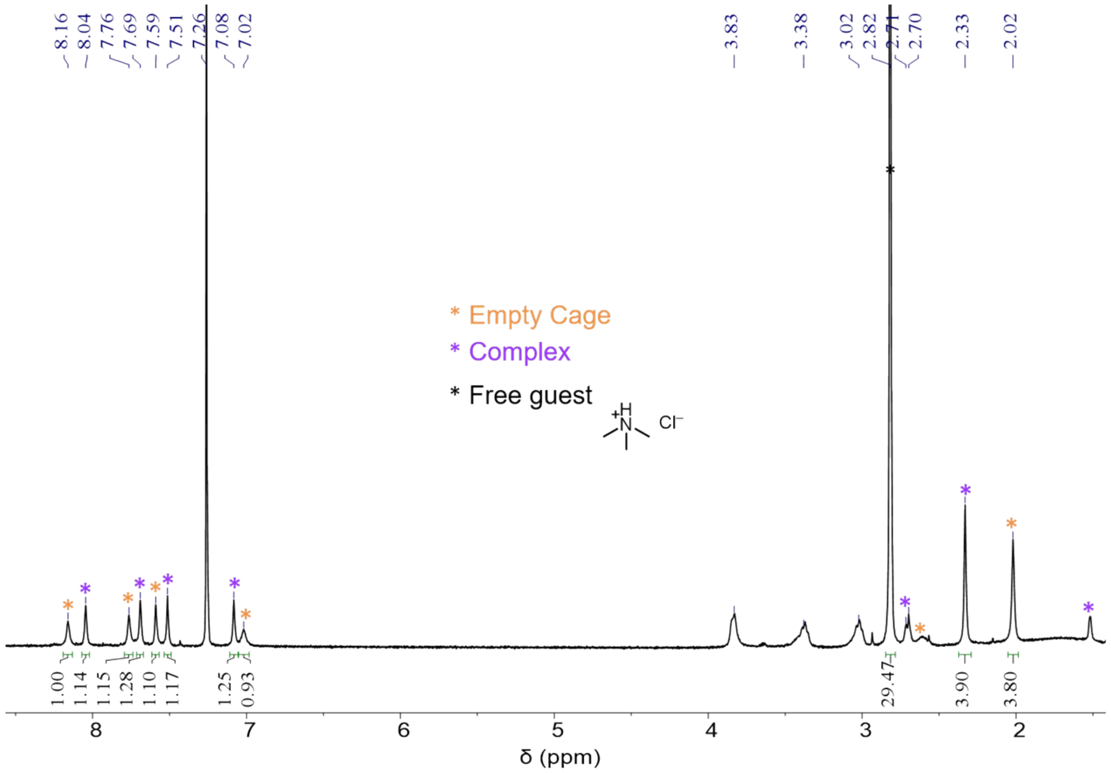

Fig. S94. ^{1}H NMR spectrum of **T1** in the presence of 19.5 equiv. of TMAH^{+}•Cl^{-}. The resonances labelled with orange, violet and black stars corresponding the empty cage, complex and free guest, respectively. The initial concentration of **T1** is 5.8 $\times 10^{-4}$ M. The binding constant *K*a is calculated to be $(1.06\pm0.18)\times10^{2}$ M^{-1} , by integrating and comparing the resonances corresponding to the complex, free guest and empty cage.

(12) Transition metal ions with cage **T1**.

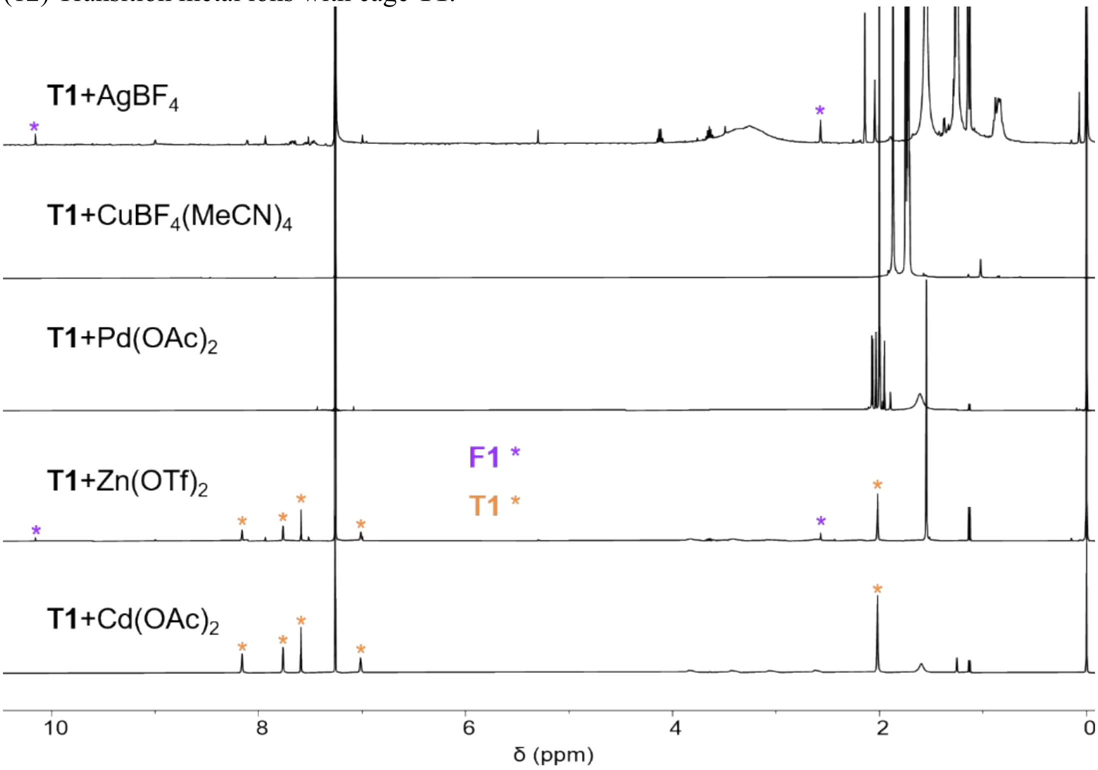

Fig S95. ^{1}H NMR spectra of **T1** after adding different transition metal cations.

The results are summarized as follows:

1. **Addition of Cu^+ and Pd^{2+}**: Upon introducing Cu^+ and Pd^{2+}, rapid precipitation was observed. The ^{1}H NMR spectrum of the resulting mixture exhibited nearly no detectable signals, suggesting that these metal ions likely coordinate with the pyridyl units across multiple cage molecules, leading to the formation of insoluble polymeric aggregates. This behavior is consistent with the structural flexibility of the cage, as the twelve pyridyl units can freely rotate, exposing their nitrogen atoms for potential metal coordination.
2. **Addition of Ag^+ and Zn^{2+}**: In the presence of Ag^+ and Zn^{2+}, the ^{1}H NMR spectrum revealed resonances corresponding to the aldehyde precursor (**F1**), indicating partial cage decomposition. This observation aligns with prior reports demonstrating that these metal ions can bind to the triamine precursor **TREN**[2].
3. **Addition of Cd^{2+}**: When $Cd(OAc)_2$ was introduced into the $CDCl_3$ solution of the cage, only a minimal amount dissolved due to its poor solubility. A slight precipitate formed, suggesting the possible formation of cage-Cd^{2+} coordination polymers. Nevertheless, the ^{1}H NMR spectrum of the soluble fraction retained the characteristic cage signals, indicating that the majority of the cage remained intact under these conditions where only small amount of Cd^{2+} was dissolved.

6. X-ray crystallography

Methods

Single crystals of **T1**, suitable for X-ray crystallography, were grown by slow vapor diffusion of isopropyl ether into a solution of **T1** in chloroform after five days. Data were collected at 170 K on a Bruker D8 Venture Diffractometer equipped with a GaKα IμS source and MX optic.

Crystal parameters of T1

[$C_{134}H_{134}N_{28}Cl_6$] (M =2349.38 g/mol), white needle (0.2 x 0.03 x 0.02 mm^3), triclinic, space group P-1 (no. 2), a = 13.0765(8) Å, b = 17.2821(11) Å, c = 30.4781(19) Å, α = 75.352(2)°, β = 81.600(2)°, γ = 71.934(2)°, V = 6318.2(7) Å^3, Z = 2, T = 170.00 K, μ(GaKα) = 1.145 mm^{-1}, $Dcalc$ = 1.235 g/cm^3, 69675 reflections measured (2.614° ≤ 2Θ ≤ 104.524°), 21558 unique (R_{int} = 0.0975, R_{sigma} = 0.1379) which were used in all calculations. The final R_1 was 0.0922 (I > 2σ(I)) and wR_2 was 0.3302 (all data). The structure was solved by direct method and different Fourier syntheses. Using Olex2, the structure was solved with the ShelXT structure solution program using Intrinsic Phasing and refined with the ShelXL refinement package using Least Squares minimization. The SQUEEZE procedure was done (see details in the cif file). CCDC number: 2395426.

Solid-state structure of T1

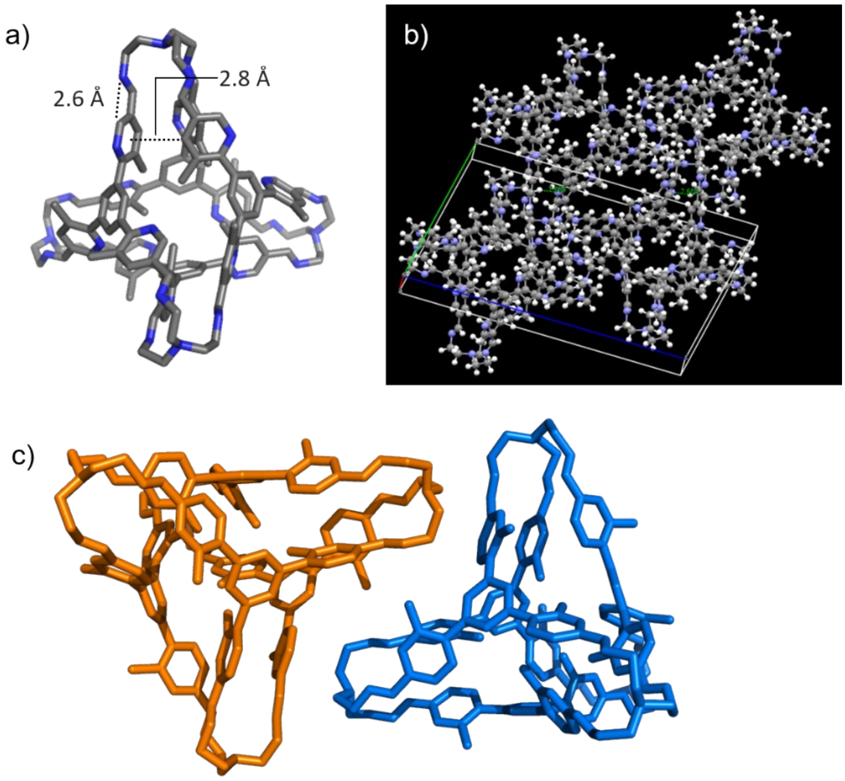

Fig. S96. Single-crystal structure of a) **T1**. Color code: Carbon, grey; nitrogen blue. Disordered solvent molecules and hydrogen atoms are omitted for clarity. b) View of the packing of the cages with intermolecular hydrogen bonding 2.654 Å between pyridine and adjacent cage. c) View of the packing of the cages with enantiomers (4*P*) and (4*M*) configurations are colored orange and blue, respectively.

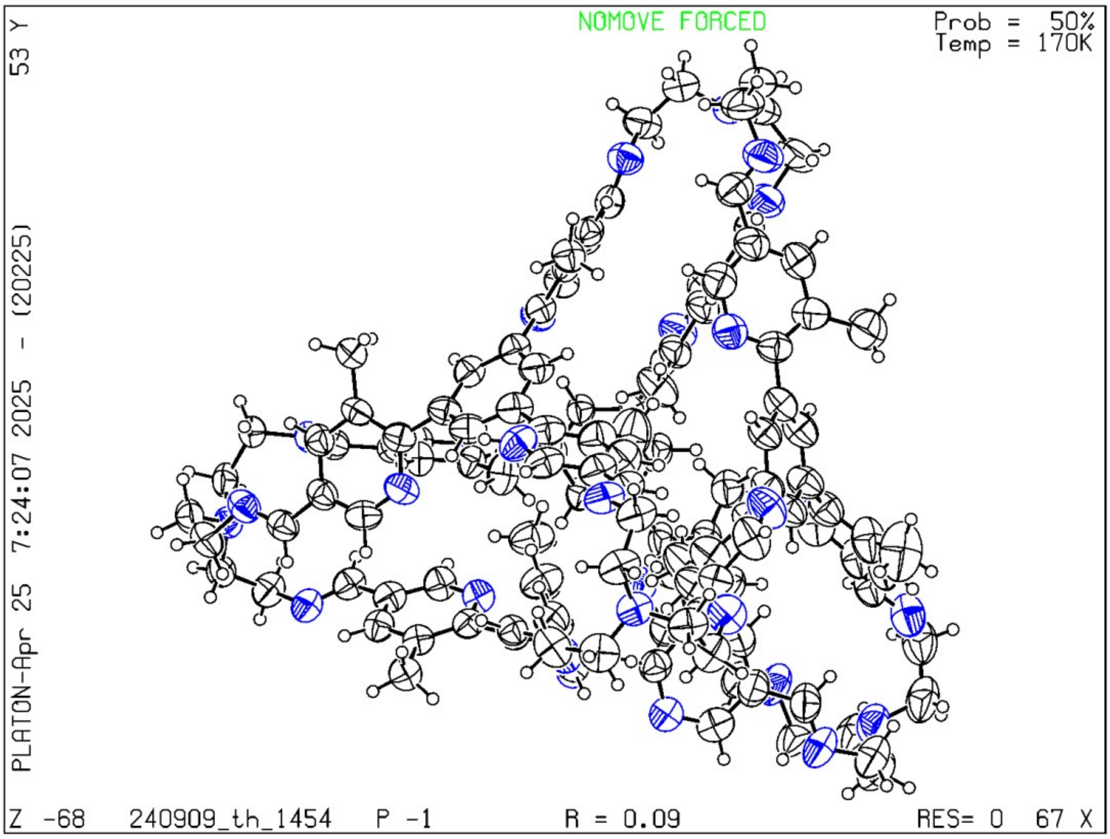

Fig. S97. Thermal ellipsoid plot for **T1** crystal. Color code: Carbon, grey; nitrogen blue. And displacement ellipsoids are drawn at the 50% probability level.

Methods

Single crystals of TMA$^+$⊂**T1**•Cl$^–$, suitable for X-ray crystallography, were grown by slow vapor diffusion of isopropyl ether into a solution of TMA$^+$•Cl$^–$ in **T1** in chloroform after three days. Data were collected at 170 K on a Bruker D8 Venture Diffractometer equipped with a GaKα IμS source and MX optic.

Crystal parameters of TMA$^+$⊂**T1**•Cl$^–$

[$C_{169}H_{217}Cl_{10.02}N_{29}O_5$] ($M$ =3089.77 g/mol), yellow block (0.39 x 0.35 x 0.23 mm^3): cubic, space group Fd-3 (no. 203), a = 32.4774(7) Å, V = 34257(2) Å^3, Z = 8, T = 170.00 K, μ(GaKα) = 1.327 mm^{-1}, $Dcalc$ = 1.198 g/cm^3, 42087 reflections measured (4.1° ≤ 2Θ ≤ 120.758°), 3283 unique (R_{int} = 0.0559, R_{sigma} = 0.0474) which were used in all calculations. The final R_1 was 0.0888 (I > 2σ(I)) and wR_2 was 0.3190 (all data). The structure was solved by direct method and different Fourier syntheses. Using Olex2, the structure was solved with the ShelXT structure solution program using Intrinsic Phasing and refined with the ShelXL refinement package using Least Squares minimization. The SQUEEZE procedure was done (see details in the cif file). CCDC number: 2441591.

Solid-state structure of TMA$^+$⊂T1•Cl$^–$

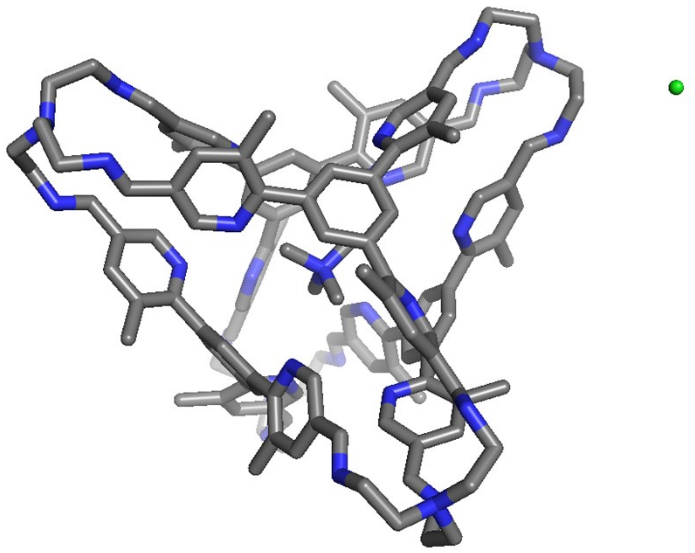

Fig. S98. Single-crystal structure of TMA$^+$⊂**T1**•Cl$^–$. Color code: Carbon, grey; nitrogen blue; chlorine, green. Disordered solvent molecules and hydrogen atoms are omitted for clarity.

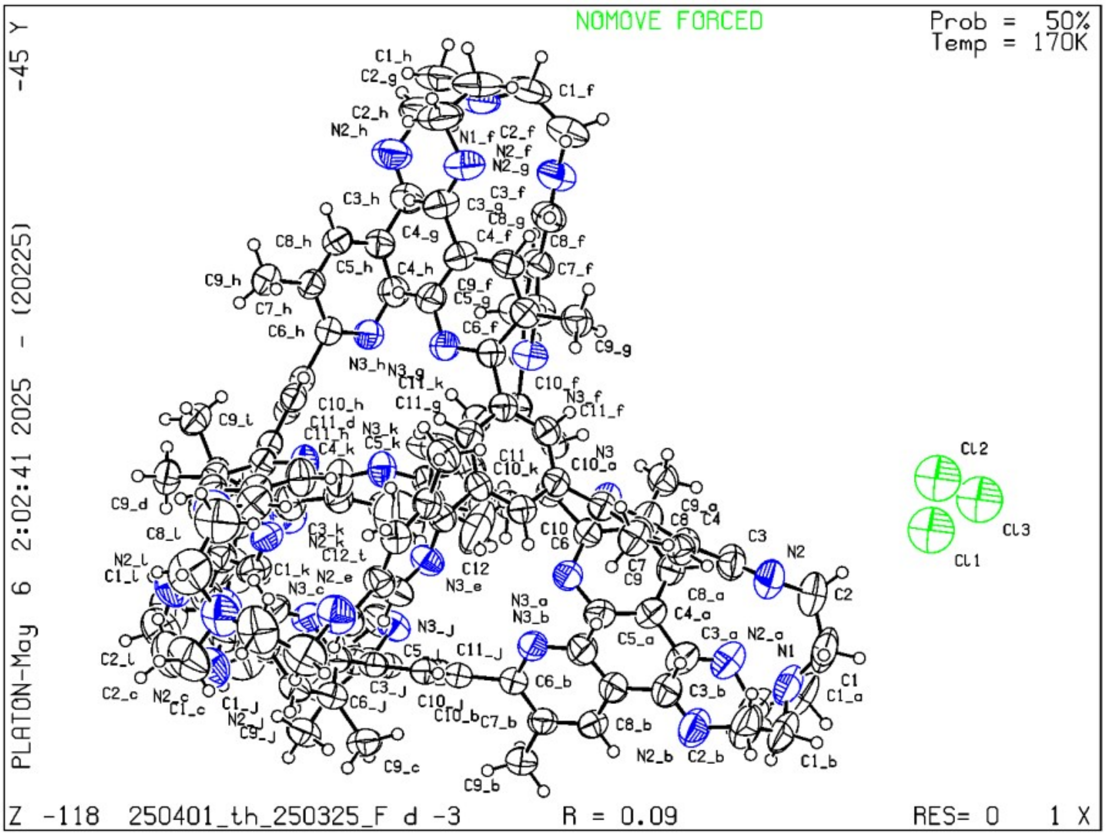

Fig. S99. Thermal ellipsoid plot for TMA$^+$⊂**T1**•Cl$^-$ crystal. Color code: Carbon, grey; nitrogen blue; chlorine, green. And displacement ellipsoids are drawn at the 50% probability level.

Methods

Single crystals of $(Cs^+)_2$⊂**T1**•$2NTf_2^-$, suitable for X-ray crystallography, were grown by slow vapor diffusion of isopropyl ether into a solution of $CsNTf_2$ in **T1** in chloroform after a week. Data were collected at 170 K on a Bruker D8 Venture Diffractometer equipped with a GaKα IμS source and MX optic.

Crystal parameters of $(Cs^+)_2$⊂T1•$2NTf_2^-$

$C_{155}H_{183}Cl_3Cs_2F_{12}N_{30}O_{15}S_4$ (M =3434.71 g/mol), colorless block (0.16 × 0.13 × 0.08 mm^3): triclinic, space group P-1 (no. 2), a = 15.9658(10) Å, b = 16.1176(10) Å, c = 33.451(2) Å, α = 81.578(2)°, β = 82.615(2)°, γ = 84.009(2)°, V = 8412.2(9) Å^3, Z = 2, T = 170.00 K, μ(GaKα) = 3.315 mm^{-1}, $Dcalc$ = 1.356 g/cm^3, 137621 reflections measured (4.678° ≤ 2Θ ≤ 114.402°), 34409 unique (R_{int} = 0.0626, R_{sigma} = 0.0683) which were used in all calculations. The final R_1 was 0.0680 (I > 2σ(I)) and wR_2 was 0.2232 (all data). The structure was solved by direct method and different Fourier syntheses. Using Olex2, the structure was solved with the ShelXT structure solution program using Intrinsic Phasing and refined with the ShelXL refinement package using Least Squares minimization. The SQUEEZE procedure was done (see details in the cif file). CCDC number: 2396911.

Solid-state structure of $(Cs^+)_2$⊂T1•$2NTf_2^-$

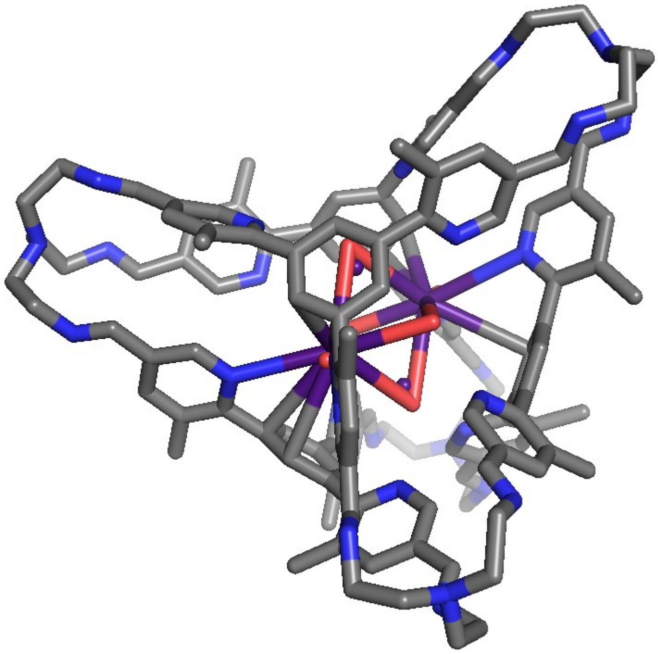

Fig. S100. Single-crystal structure of $(Cs^+)_2$⊂**T1**•$2NTf_2^-$. Color code: Carbon, grey; nitrogen, blue; caesium, cyan; oxygen, red. Disordered solvent molecules and hydrogen atoms are omitted for clarity.

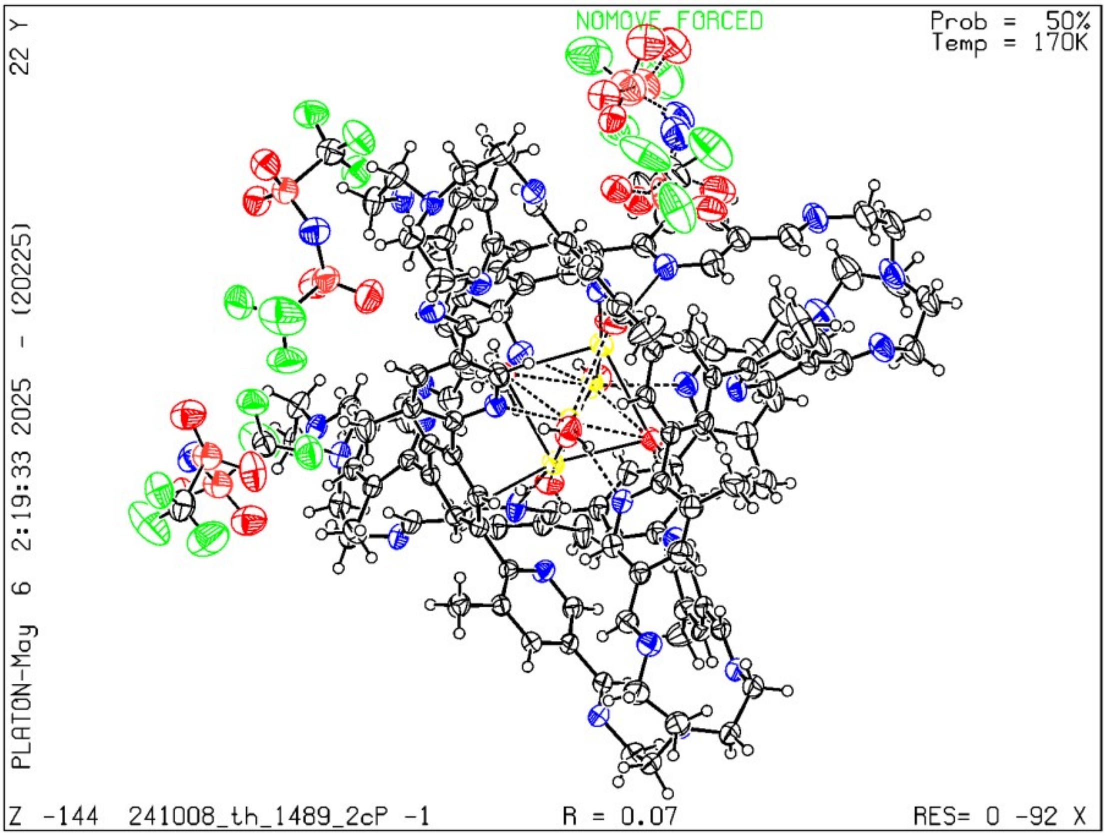

Fig. S101. Thermal ellipsoid plot for $(Cs^{+})_2$⊂**T1**•$2NTf_2^{-}$ crystal. Color code: Carbon, grey; nitrogen blue; caesium, yellow; oxygen, red; phosphorus, red; fluorine, green. And displacement ellipsoids are drawn at the 50% probability level.

7. References

1. Y. Peng, S. Tong, Y. Zhang and M.-X. Wang, Functionalized Hydrocarbon Belts: Synthesis, Structure and Properties, *Angew. Chem. Int. Ed.*, 2023, **62**, e202302646.
2. B. Doistau, J.-L. Cantin, L.-M. Chamoreau, V. Marvaud, B. Hasenknopf and G. Vives, Mechanical switching of magnetic interaction by tweezers-type complex, *Chem. Commun.*, 2015, **51**, 12916-12919.
